# Phase II study of talazoparib in advanced cancers with BRCA1/2, DNA repair, and PTEN alterations

**DOI:** 10.1038/s41698-024-00634-6

**Published:** 2024-07-31

**Authors:** Sarina A. Piha-Paul, Chieh Tseng, Cheuk Hong Leung, Ying Yuan, Daniel D. Karp, Vivek Subbiah, David Hong, Siqing Fu, Aung Naing, Jordi Rodon, Milind Javle, Jaffer A. Ajani, Kanwal P. Raghav, Neeta Somaiah, Gordon B. Mills, Apostolia M. Tsimberidou, Xiaofeng Zheng, Ken Chen, Funda Meric-Bernstam

**Affiliations:** 1 Department of Investigational Cancer Therapeutics (A Phase I Clinical Trials Program), University of Texas MD Anderson Cancer Center, Houston, TX USA; 2 Department of Biostatistics, University of Texas MD Anderson Cancer Center, Houston, TX USA; 3 Department of Gastrointestinal Medical Oncology, University of Texas MD Anderson Cancer Center, Houston, TX USA; 4 Department of Sarcoma Medical Oncology, University of Texas MD Anderson Cancer Center, Houston, TX USA; 5 Knight Cancer Institute, Oregon Health Sciences University, Portland, OR USA; 6 Department of Breast Surgical Oncology, University of Texas MD Anderson Cancer Center, Houston, TX USA; 7 Department of Bioinformatics and Computational Biology, University of Texas MD Anderson Cancer Center, Houston, TX USA; 8 The Sheikh Khalifa Bin Zayed Al Nahyan Institute for Personalized Cancer Therapy, University of Texas MD Anderson Cancer Center, Houston, TX USA

**Keywords:** Targeted therapies, Targeted therapies

## Abstract

Cancer cells with *BRCA1/2* deficiencies are sensitive to poly (ADP-ribose) polymerase (PARP) inhibitors. We evaluated the efficacy of talazoparib in DNA-Damage Repair (DDR)-altered patients. In this phase II trial, patients were enrolled onto one of four cohorts based on molecular alterations: (1) somatic *BRCA*1/2, (2) other homologous recombination repair pathway, (3) *PTEN* and (4) germline *BRCA1/2*. The primary endpoint was a clinical benefit rate (CBR): complete response, partial response or stable disease ≥24 weeks. 79 patients with a median of 4 lines of therapy were enrolled. CBR for cohorts 1–4 were: 32.5%, 19.7%, 9.4% and 30.6%, respectively. *PTEN* mutations correlated with reduced survival and a trend towards shorter time to progression.Talazoparib demonstrated clinical benefit in selected DDR-altered patients. *PTEN* mutations/loss patients derived limited clinical benefit. Further study is needed to determine whether PTEN is prognostic or predictive of response to PARP inhibitors.

## Introduction

Cancer cells frequently have defects in deoxyribose nucleic acid (DNA) repair pathways^1,2^. Cells deficient in breast cancer gene 1 or 2 (*BRCA1* or *BRCA2)* function have a high level of chromosomal instability^3–5^. Poly (ADP-ribose) polymerase (PARP) inhibitors have been shown to be selectively lethal to cells lacking functional *BRCA1* or *BRCA2* with minimal toxicity to normal cells^6^. Further, tumors with germline *BRCA* mutations demonstrated defects in homologous recombination (HR) and have been shown in several trials to respond to PARP inhibitors^7,8^.

Preclinical studies suggest that cancer cells with alterations in other homologous recombination (HR) repair pathway genes (e.g., ataxia telangiectasia mutated (*ATM*) and partner and localizer of *BRCA2* (*PALB2*) may also be sensitive to PARP inhibition^9–12^. ATM is recruited and activated by DNA double-strand breaks where it phosphorylates several proteins that are important in activating the DNA damage checkpoint leading to cell cycle arrest, DNA repair or apoptosis. Heterozygous germline mutation of *ATM* is a moderate-risk factor for developing breast cancer^11^. Montani and colleagues found that ATM-depletion can sensitize breast cancer cells to PARP inhibition thus PARP inhibition could be of benefit in patients with low ATM protein expression/activity such as would be seen in heterozygous carriers of germline *ATM* mutation^11^. Also of interest, the *PALB2* gene encodes for a protein that functions in repair of double stranded DNA breaks. Patients with variants of the *PALB2* gene have an increased risk of developing breast cancer^10^. Further, cells deficient in *PALB2* have been found to be sensitive to PARP inhibitors^9^.

Additional genes such as *Fanconi Anemia (FA)* genes were of interest for this study as identification of the *FANCD1* gene as *BRCA2* provided the first direct link between FA proteins and DNA repair^13,14^. Because *BRCA2* deficient cells are sensitive to PARP inhibition, we wished to determine whether or not tumors that have deleterious alterations of the *FA* gene family would share this trait.

Phosphatase and tensin homolog (*PTEN*) acts as a tumor suppressor and mutations of this gene are a step in the development of many cancers. *PTEN* is important in the regulation of the cell cycle thus preventing the rapid growth and division of cells^15^. Studies have shown that *PTEN* deficiency caused a homologous repair deficiency, potentially due to downregulation of *Rad51*, a critical HR component^16,17^. Further, *PTEN* loss has been shown to be synthetic lethal with PARP, with *PTEN* loss sensitizing to PARP inhibitors in vitro and in vivo^16^. However, the role of *PTEN* remains controversial, as some data suggest that it may not regulate *Rad51* expression or HR function. Additionally, *PTEN* inactivation has been found to render *BRCA1/2* aberrant cells resistant to PARP^18,19^.

Talazoparib exerts its antineoplastic effects through the selective inhibition of PARP enzymes, which are pivotal in the single-strand DNA break repair mechanism^20^. The pharmacological blockade of PARP by talazoparib leads to an accumulation of DNA lesions, precipitating the formation of double-strand breaks. In neoplastic cells characterized by mutations in *BRCA1/2*, the intrinsic defect in the homologous recombination repair pathway renders these double-strand breaks irreparable. This culminates in heightened genomic instability and triggers apoptosis, a process termed synthetic lethality^20,21^. This mechanism is particularly effective against cancer cells harboring specific genetic aberrations in DNA repair pathways. Notably, talazoparib exhibits a distinct pharmacodynamic property of forming PARP-DNA complexes, further potentiating its cytotoxic profile^22^. This attribute of talazoparib underscores its clinical utility, leading to its approval by the U.S. Food and Drug Administration (FDA) in October 2018 for the management of HER2-negative advanced breast cancers with deleterious or suspected pathogenic mutations in the *BRCA* genes^23^. Furthermore, talazoparib is more potent at lower concentrations than earlier generation PARP1/2 inhibitors (such as olaparib, rucaparib and veliparib)^24,25^.

In recent years, mounting clinical evidence has substantiated the rationale for extending the application of talazoparib beyond the confines of *BRCA1/2* mutation-positive malignancies, aligning with our trial’s molecularly driven strategy. This extension is predicated on the paradigm of ‘BRCAness’, where tumors share similar vulnerabilities to *BRCA* mutant cancers due to deficiencies in the DNA damage repair mechanisms^20,26^. For instance, a meta-analytic survey encompassing 21 diverse tumor types identified that a significant subset (17.4%) exhibits mutations in homologous recombination repair (HRR)-associated genes, thus providing compelling rationale for exploring talazoparib in these broader oncogenic contexts^27^. Notably, primary prostate cancers exhibit a HR deficiency mutation prevalence of approximately 20%, with higher incidence observed in metastatic lesions, ranging up to 23%^28^. These findings underscore the need to re-evaluate and expand current oncological paradigms for the clinical deployment of PARP inhibitors, reflecting our study’s commitment to a molecularly centered therapeutic approach^22–25,29,30^.

Based on this scientific background, we conducted a phase II study to formally evaluate whether talazoparib achieves clinical benefit (complete response (CR), partial response (PR) or stable disease (SD) ≥ 24 weeks) in metastatic or inoperable locally advanced or locally recurrent cancer patients who have germline mutations in *BRCA1* or *BRCA2* with cancers other than breast or ovarian cancer, *PTEN* mutation/deletion or loss by IHC and in patients with somatic mutations or homologous deletions in *BRCA1* or *BRCA2*, or alterations in other HR repair pathway genes.

## Results

### Demographic and clinical characteristics

A total of 94 patients consented to participate in the study. However, 15 were excluded: 12 patients screen-failed and 3 withdrew their consent prior to enrollment. Seventy-nine patients with advanced, metastatic malignancies were enrolled and treated on study between December 2014 and July 2021 (Supplementary Fig. 1). Demographic and clinical characteristics are summarized in Table 1. The median age of patients was 61 (range, 22–84 years). The median number of prior systemic therapies was 4 (range, 1–13). No patients had received prior PARP inhibitor therapies. Fifty-seven patients (72.2%) had received prior platinum therapy. The most common cancer types enrolled were sarcoma followed by colorectal cancer, breast, and cholangiocarcinoma. The median number of cycles (cycle = 28 days) completed for all patients was 2 (range, 1–47 + ). Thirty-five patients (44%) received more than two cycles. For patients with SD or better, the median number of cycles completed was 6 (range, 2–47 + ). Pre-identified pathogenic or activating molecular aberration that were used for study enrollment are summarized in Table 1.

**Table 1:** Baseline demographics and clinical characteristics

Characteristic	Somatic *BRCA1/2*	Other HR pathways	*PTEN* mutation/loss	Germline *BRCA1/2*	All
	*N* = 18	*N* = 31	*N* = 14	*N* = 16	*N* = 79
Gender, *n* (%)					
Male	5 (28)	15 (48)	3 (21)	5 (31)	28 (35)
Female	13 (72)	16 (52)	11 (79)	11 (69)	51 (65)
Median age	59.5 (32–81)	59 (32–75)	63.5 (22–84)	61 (53–72)	61 (22–84)
ECOG					
0	3 (17)	6 (19)	4 (29)	2 (13)	15 (19)
1	15 (83)	25 (81)	10 (71)	14 (88)	64 (81)
Tumor Type					
Sarcoma^a^	3 (16.7)	5 (16.1)	3 (21.4)	0	11 (13.9)
Colorectal	2 (11.1)	4 (12.9)	1 (7.1)	3 (18.8)	10 (12.7)
Breast	3 (16.7)	2 (6.5)	2 (14.3)	0	7 (8.9)
Cholangiocarcinoma	1 (5.6)	4 (12.9)	1 (7.1)	1 (6.3)	7 (8.9)
Ovarian	2 (11.1)	3 (9.7)	1 (7.1)	0	6 (7.6)
Pancreas	0	1 (3.2)	1 (7.1)	4 (25)	6 (7.6)
Head and neck	1 (5.6)	2 (6.5)	1 (7.1)	1 (6.3)	5 (6.3)
Ampulla of Vater	0	2 (6.5)	0	1 (6.3)	3 (3.8)
Bladder	0	1 (3.2)	0	2 (12.5)	3 (3.8)
Gastric	1 (5.6)	1 (3.2)	0	1 (6.3)	3 (3.8)
Renal	1 (5.6)	1 (3.2)	0	1 (6.3)	3 (3.8)
Endometrial	0	1 (3.2)	1 (7.1)	0	2 (2.5)
Gall bladder	0	1 (3.2)	1 (7.1)	0	2 (2.5)
Lung	2 (11.1)	0	0	0	2 (2.5)
Melanoma	1 (5.6)	0	0	0	1 (1.3)
Salivary gland	1 (5.6)	0	0	0	1 (1.3)
Other	0	3 (9.7)^b^	2 (14.3)^c^	2 (12.5)^d^	7 (8.9)
Number of prior systemic therapies					
Median	4 (1–13)	3 (1–13)	5 (2–11)	5 (2–11)	4 (1–13)
Prior treatment					
Surgery	16 (89)	26 (84)	12 (86)	11 (69)	65 (82.3)
Chemotherapy	18 (100)	31 (100)	14 (100)	16 (100)	79 (100)
Radiation	14 (78)	19 (61)	10 (71)	7 (44)	50 (63.3)
Prior Platinum	13 (72)	23 (74)	9 (64)	12 (75)	57 (72.2)
Molecular Aberration for enrollment					
Somatic *BRCA1*	9 (50)	0	0	0	9 (11.4)
Somatic *BRCA2*	9 (50)	0	0	0	9 (11.4)
Germline *BRCA1*	0	0	0	8 (50)	8 (10.1)
Germline *BRCA2*	0	0	0	8 (50)	8 (10.1)
*ATM*	0	10 (32)	0	0	10 (12.7)
*FA genes*	0	5 (16)	0	0	5 (6.3)
*ARID1A*	0	5 (16)	0	0	5 (6.3)
*PALB2*	0	4 (13)	0	0	4 (5.1)
*ATR*	0	2 (6.5)	0	0	2 (2.5)
*PTEN* mutation	0	0	8 (57)	0	8 (10.1)
*PTEN* gene loss			2 (14)	0	2 (2.5)
PTEN loss by IHC			4 (29)^f^	0	4 (5.1)
Other^e^	0	5 (16)	0	0	5 (6.3)

*ARID1A* AT-Rich Interaction Domain 1A, *ATM* Ataxia-Telangiectasia Mutated, *ATR* Ataxia Telangiectasia and Rad3 related, *BRCA* Breast Cancer gene, *FA* Fanconi Anemia, *PALB2* Partner and Localizer of BRCA2, *PTEN* Phosphatase and Tensin homolog, *IHC* immunohistochemistry, *ECOG* Eastern Cooperative Oncology Group.

^a^includes angiosarcoma (2), spindle cell (1), leiomyosarcoma (4), pleomorphic liposarcoma (1), desmoplastic small round cell (1), soft tissue sarcoma (1) and synovial sarcoma (1).

^b^includes urachal (1), appendiceal (1) and unknown primary (1).

^c^ includes squamous cell carcinoma of skin (1) and cervical (1).

^d^includes sebaceous adenocarcinoma on upper eyelid (1) and esophageal (1).

^e^includes BRIP1, BRCA1-Interacting Protein 1 (1); *EMSY*, EMSY Transcriptional Repressor (1): RAD51, RAD51 recombinase (1); BARD1, BRCA1-associated ring domain protein 1 (1); and BAP1, BRCA 1-associated protein 1 (1).

^f^one patient had co-occurring PTEN_Q245*/ PTEN loss by IHC.

### Efficacy

Table 2 summarizes mean clinical benefit rate (CBR) which is the primary end point of study. Among the 79 patients treated across the study, there was one (1.3%) CR, seven (8.9%) PRs and ten (12.7%) SD ≥ 24 weeks. A total of 18/79 (22.8%) patients derived clinical benefit (CR/PR/SD ≥ 24 weeks). The overall response rate (ORR) was 10.1% (8/79; 95% CI: 4.5%, 19.0%). Table 3 shows detail on molecular alterations, cohort assignment, duration of treatment and best response by RECIST v1.1 in patients that demonstrated clinical benefit. Supplementary Table 4 shows a breakdown of ORR and CBR by tumor type.

**Table 2:** Treatment response per cohort as assessed by RECIST v1.1

Response category	Somatic *BRCA1/2* Mutation (*N* = 18)	Other HR repair pathway gene aberrations^b^ (*N* = 31)	*PTEN* Mutation or Loss by IHC (*N* = 14)	Germline *BRCA1/2* Mutation (N = 16)	All (*N* = 79)
Mean CBR^a^ - *n* (%)	6 (32.5) (90% credible interval: 16.7, 50.3)	6 (19.7) (90% credible interval: 9.6, 31.9)	1 (9.4) (90% credible interval: 1.2, 23.3)	5 (30.6) (90% credible interval: 14.5, 49.2)	18 (22.8)
ORR - *n* (%) (95% CI)	4 (22.2) (6.4, 47.6)	3 (9.7) (2.0, 25.8)	0	1 (6.3) (0.2, 30.2)	8 (10.1) (4.5, 19.0)
CR - *n* (%)	1 (5.6)	0	0	0	1 (1.3)
PR - *n* (%)	3 (16.7)	3 (9.7)	0	1 (6.3)	7 (8.9)
SD ≥ 24 weeks - *n* (%)	2 (11.1)	3 (9.7)	1 (7.1)	4 (25.0)	10 (12.7)
SD < 24 weeks - *n* (%)	4 (22.2)	5 (16.1)	2 (14.3)	6 (37.5)	17 (21.5)
PD - *n* (%)	8 (44.4)	17 (54.8)	10 (71.4)	3 (18.8)	38 (48.1)
NE - *n* (%)	0	3 (9.7)	1 (7.1)	2 (12.5)	6 (7.6)
Median PFS (weeks) (95% CI)	11.5 (7.9, 23.3)	7.9 (7.7, 21.1)	7.7 (7.6, 9.9)	23.6 (7.4, 33.9)	9.6 (7.7, 15.9)
PFS at 4 weeks (%) (95% CI)	94 (84, 100)	90 (80, 100)	93 (80, 100)	88 (73, 100)	91 (85, 98)
PFS at 8 weeks (%) (95% CI)	61 (42, 88)	43 (29, 65)	29 (12, 65)	69 (49, 96)	50 (40, 62)
Median OS (months) (95% CI)	14.6 (5.4, 36.4)	8.9 (6.0, 11.8)	8.5 (5.1, 102)	12.3 (7.6, 21.1)	10.3 (8.5, 12.9)
OS at 6 months (%) (95% CI)	71 (52, 96)	67 (52, 86)	69 (48, 100)	81 (64, 100)	71 (62, 82)
OS at 12 months (%) (95% CI)	65 (46, 92)	32 (19, 54)	23 (9, 62)	50 (31, 82)	42 (32, 55)

*RECIST v1.1* response evaluation criteria in solid tumors guideline version 1.1, *HR* Homologous Recombination, *IHC* immunohistochemistry, *CBR* clinical benefit rate, *ORR* objective response rate, *PFS* progression-free survival, *OS* overall survival, *CR* complete response, *PR* partial response, *SD* stable disease, *PD* progressive disease, *NE* non-evaluable, *BRCA Breast Cancer gene*, *PTEN* Phosphatase and Tensin homolog, *CI* confident interval, *n* number.

^a^The mean clinical benefit rate in each cohort was assessed using posterior probabilities with corresponding credible interval.

^b^Includes mutations, deletions and amplifications.

**Table 3:** Patients with SD ≥ 24 weeks, CR or PR by RECIST v1.1

Subject ID	Cancer Type	Cohort Assignment	Cohort Specific Molecular Alteration	Best Response by RECIST v1.1	# of Prior Systemic Therapies	Duration of Treatment (weeks)	Response length (weeks)
01-040	Leiomyosarcoma of uterus	1	BRCA2 S2186fs*3	−1% (SD)	4	32	24.1
01-048	Soft tissue sarcoma	1	BRCA1 V788fs*10	−54% (PR)	6	23	7.4
01-061	Ovarian	1	*BRCA2 R3052W*	−100% (CR)	3	182+	31+
01-073	Breast	1	BRCA1 Q356*	−14% (SD)	7	40	32.6
01-079	Salivary Gland	1	*BRCA1_M1I*	−73% (PR)	2	31	23.4
01-082	Breast Cancer	1	BRCA1_Q380*	−81% (PR)	3	112+	88.6+
02-054	Appendiceal	2	ATM S1599*	−33% (PR)	2	61	12
02-007	Ovarian	2	*BRIP1* (FANCJ) A402Vfs*21	3% (SD)	4	34	26.1
02-032	Cholangiocarcinoma	2	*ATM_ G1676fs*	−38% (PR)	2	60	16
02-033	Myoxid Spindle Cell sarcoma	2	*FANCC Loss of exon 28*	−10% (SD)	3	25	24.6
02-035	Urachal adenocarcinoma	2	*PALB2 R170fs14*	−35% (PR)	3	25	17
02-062	GE Junction	2	ATM deletion ATM E2052*	8% (SD)	3	31	24
03-008	Gallbladder	3	*PTEN L70F*	5% (SD)	6	32	24
04-034	Cholangiocarcinoma	4	*BRCA2 2041insA*	−14% (SD)	6	34	25.7
04-029	Ampulla of Vater	4	*BRCA2_c.8633-1* *G* > *C*	−20% (SD)	2	124	116.3
04-052	Adenocarcinoma of cecum	4	BRCA2 p.K944*	−100% (PR)^a^	2	62	25.1
04-065	Gastric adenocarcinoma	4	*BRCA2 c.7878* *G* > *A p.Trp2628Ter*	−11% (SD)	4	87	81
04-075	Pancreatic	4	BRCA 2 D252Vfs*24	−11% (SD)	4	44	36.4

*ARID1A* AT-Rich Interaction Domain 1A, *ARFRP1* ADP Ribosylation factor related protein 1, *ATM* Ataxia-Telangiectasia Mutated, *BRCA* Breast Cancer gene, *BRIP1* BRCA1-Interacting Protein 1, *FANCC* Fanconi Anemia Complementation group C, *FANCJ* Fanconi Anemia Complementation group J, *PALB2* Partner and Localizer of BRCA2, *Trp* tryptophan, *Ter* termination, *ID* identifier, *CR* complete response, *PR* partial response, *SD* stable disease.

^a^Subject ID 04-052 was considered PR given persistence of ascites.

**Fig. 1:**
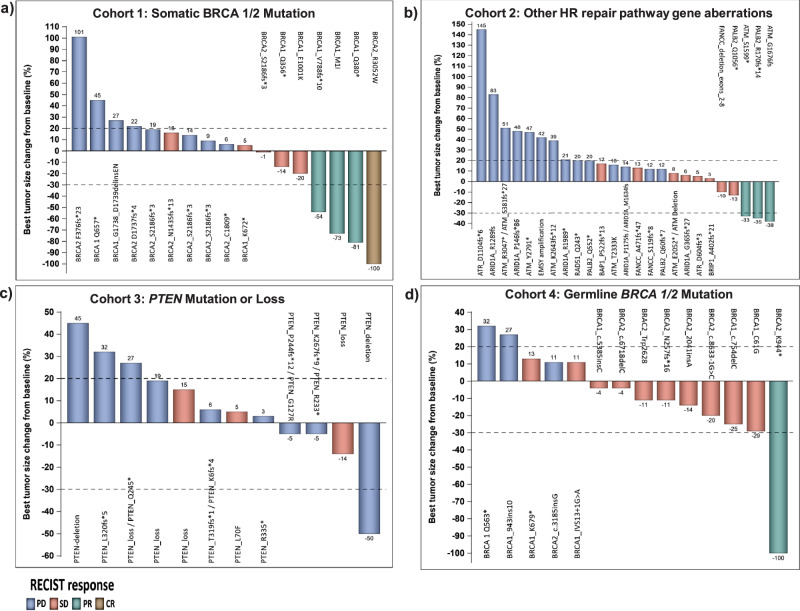
Waterfall plots depicting best RECIST response by patient broken down by cohort. Individual patients are represented by vertical bars on the X-axis. The best RECIST response (%) is depicted on the Y-axis. Of 79 patients treated on trial, 68/79 (86.1%) had disease that was measurable by RECISTv1.1 and had post-baseline radiographic imaging studies performed. Molecular aberrations corresponding to the enrolled cohorts are shown on the top of each waterfall plot. Patients with somatic *BRCA1/2* mutations (cohort 1) are shown in Fig. 1**a**; patients with other HR repair pathway mutations (cohort 2) are shown in Fig. 1**b**; patient with *PTEN* mutation/loss (cohort 3) are shown in Fig. 1**c**; and patients with germline *BRCA1/2* mutations (cohort 4) are shown in Fig. 1**d**.

Of 79 patients treated on trial, 68/79 (86.1%) had disease that was measurable by RECISTv1.1 and had post-baseline radiographic imaging studies performed. However, 11/79 did not complete tumor re-assessment and were not included in the waterfall plots (Fig. 1, Supplementary Fig. 1). Evaluation of tumor response per RECIST v1.1 is shown in Fig. 1 and Table 2. In Fig. 1, waterfall plots depict the best RECIST response per patient broken down by cohort. In cohort 1 (Fig. 1a and Table 2), six of 18 patients had a mean CBR of 32.5% (90% credible interval: 16.7%, 50.3%), including one with CR, three with PR and two with prolonged SD. In cohort 2 (Fig. 1b and Table 2), six of 31 patients had a mean CBR of 19.7% (90% credible interval: 9.6%, 31.9%) including three patients with PR and three with prolonged SD. Limited CBR was observed in patients with *PTEN* mutation/loss in cohort 3 (Fig. 1c and Table 2). Among these patients, one with gallbladder cancer and somatic *PTEN* L70F mutation had prolonged SD (32 weeks). The mean CBR for cohort 3 was 9.4% (90% credible interval: 1.2%, 23.3%). In cohort 4 (Fig. 1d and Table 2), the mean CBR was 30.6% (90% credible interval: 14.5%, 49.2%) including one patient with PR and 4 with prolonged SD.

The study had a median follow-up duration of 9.9 months, with data cut-off occurring on August 1, 2022. The median PFS on this trial was 9.6 weeks (95% CI: 7.7, 15.9 weeks). The median OS across all cohorts was 10.3 months (95% CI: 8.5, 12.9 months). The median DOR was 6.3 months (95% CI: 1.7, not reached) in the eight patients that were objective responders (CR or PR).

**Fig. 2:**
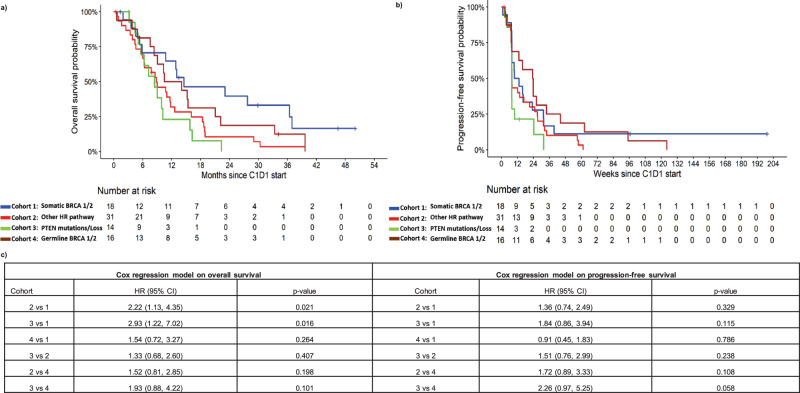
Overall survival and progression free survival analysis. Patients were grouped by mutation status/cohort. **a** Kaplan–Meier plot of overall survival. **b** Kaplan–Meier plot of progression-free survival. **c** Table shows the hazard ratios and *p*-value from Cox proportional hazards regression model.

We found that patients with *PTEN* mutation exhibited a higher risk of death and disease progression compared to patients with *PTEN* wildtype (*WT*) status. Our prespecified cohort survival analyses revealed that the risk of death among the patients in cohort 3 was 2.93 (95% CI: 1.22, 7.02, p = 0.016) times the risk among that of cohort 1, 1.33 (95% CI: 0.68, 2.60, p = 0.407) times that of cohort 2, and 1.93 (95% CI: 0.88, 4.22, p = 0.101) times that of cohort 4 (Fig. 2a, c). Similarly, the risk of disease progression among the *PTEN* mutation/loss (cohort 3) was higher with a hazard ratio of 1.84 (95% CI: 0.86, 3.94, p = 0.115) times that of cohort 1, 1.51 (95% CI; 0.76, 2.99, p = 0.238) times that of cohort 2 and 2.26 (95% CI:0.97,5.25, p = 0.058) times that of cohort 4 (Fig. 2b, c).

### PTEN analysis

**Fig. 3:**
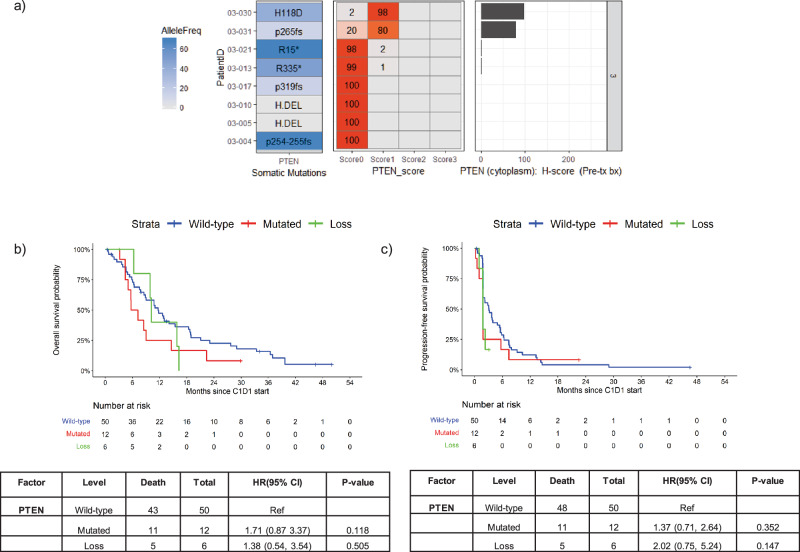
*PTEN* status and overall survival and progression free survival analysis. **a** *PTEN* mutation status and PTEN-IHC results are shown in tile and horizontal bar plots. *PTEN* mutations are represented in the left tile plot with the allelic frequency (AlleleFreq) of somatic mutations represented by the fill and the amino acid change indicated by text. *PTEN* copy number deletions are indicated as H.DEL. The middle tile plot represents the percentage of cells with the respective PTEN-IHC staining level; score 0 = no staining, score 1 = low, score 2 = intermediate, and score 3 = high staining levels. The right horizontal bar plot represents the H-score calculated from the PTEN-IHC staining levels. Of note, patients with either *PTEN* truncating mutations or H.DEL had PTEN loss on IHC testing. Additionally, two of three patients with *PTEN* frameshift mutations also had PTEN loss by IHC. **b** Kaplan–Meier plot of overall survival. Sixty-eight patients were stratified by PTEN status (either wild-type, mutated or deletion/loss by IHC). Table shows the hazard ratios and *p*-value from Cox proportional hazards regression model. **c** Kaplan–Meier plot of progression-free survival. Sixty-eight patients were stratified by PTEN status (either wild-type, mutated or deletion/loss by IHC). Table shows the hazard ratios and *p*-value from Cox proportional hazards regression model.

PTEN IHC was performed for 8 of the 14 patients enrolled to cohort 3 that had pre-treatment biopsy tissue available. PTEN IHC levels overall correlated with mutation and copy number status. All patients with deleterious *PTEN* mutations or deletion had either no or low PTEN expression (Fig. 3a).

To investigate the impact of *PTEN* status, at study enrollment, on the efficacy of talazoparib, we conducted a comparative analysis of patients with *PTEN WT* versus those with deleterious *PTEN* alterations including PTEN loss by IHC. The analysis encompassed 70 of the 79 patients whose *PTEN* status was known at the time of enrollment. Amongst these 70 patients, 50 (71.4%) exhibited *PTEN WT*, while 18 (25.7%) displayed deleterious PTEN alterations, which included 6 cases of *PTEN* deletion or loss by IHC and 12 cases of *PTEN* inactivating mutations. Two patients from cohort 2 harbored *PTEN* mutations of indeterminate significance and were excluded from the overall survival (OS) and progression-free survival (PFS) evaluations. This stratification allowed for a more nuanced understanding of the impact of specific *PTEN* deleterious alterations on patient outcomes when treated with talazoparib.

Our analysis revealed a correlation between *PTEN* inactivating alterations and diminished survival outcomes, including OS and PFS. We observed that the median OS varied across the groups, with PTEN *WT* patients showing a median OS of 11.7 months (95% CI: 7.8, 15.4). In contrast, patients with PTEN inactivating mutations had a median OS of 6.5 months (95% CI:4.4,14.6) and those with PTEN deletion or loss by IHC exhibited a median OS of 10.2 months (95% CI:6.3,16.3) (Fig. 3b).The median PFS in *PTEN WT*, mutated and PTEN deletion or loss were 3.1 months (95% CI:1.8,4.9), 1.8 months (95% CI:0.5,5.7) and 1.8 months (95% CI: 1.0, not reached), respectively. The risk of disease progression in patients with *PTEN* alterations was higher compared to those with *PTEN WT* though not statistically significant. The hazard ratios for disease progression in PTEN inactivating mutations and *PTEN* deletion or loss by IHC were 1.37 (95% CI: 0.71–2.64, p = 0.352) and 2.02 (95% CI: 0.75-5.24, p = 0.147) when compared with *PTEN WT*, respectively (Fig. 3c). Furthermore, the analysis revealed a trend towards an increase in risk of death, with a HR of 1.71 (95% CI, 0.87–3.37, p = 0.118) in *PTEN* mutated and HR of 1.38 (95% CI, 0.54–3.54, p = 0.505) in PTEN deletion or loss by IHC when compared to the *PTEN WT* group.

### Molecular analysis

**Fig. 4:**
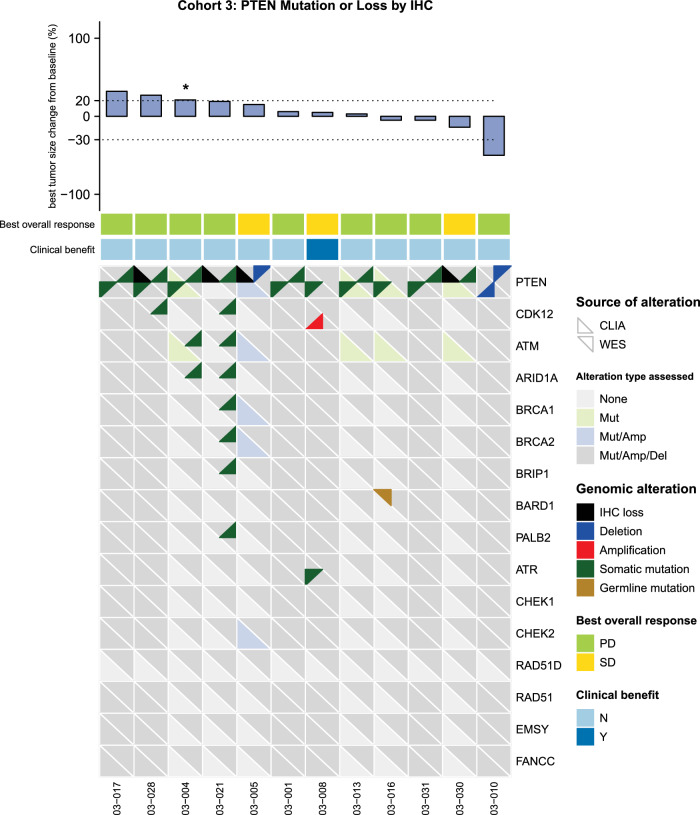
Overview of mutational landscape and treatment response of patients in cohort 3 (*PTEN* alterations and PTEN loss by IHC). The heatmap illustrates alterations detected in *PTEN* and in the DNA Damage Repair (DDR) genes (including *ATM, BRCA2, BRCA1, ARID1A, PALB2, ATR, CDK12, BARD1, CHEK1, EMSY, BRIP1, FANCC, CHEK2, RAD51D and RAD51*) with corresponding treatment response. The heatmap analysis presents results from the whole exome sequencing (WES) analysis using pre-treatment tumor biopsy samples, alongside corresponding pre-identified molecular alterations allowing for enrollment onto study (identified as CLIA alterations). Patient with clinical progression as assessed by the treating physician and no post-baseline scans was assigned a value of +21% (*).

To date, we have analyzed the pre-treatment biopsies collected on patients in cohort 3. Among 14 patients enrolled into this cohort, samples for 12 patients underwent whole exome sequencing (WES) and analysis. Our focus was on confirming molecular alterations in PTEN along with identifying alterations in DNA Damage Repair (DDR) genes including *ATM, BRCA2, BRCA1, ARID1A, PALB2, ATR, CDK12, BARD1, CHEK1, EMSY, BRIP1, FANCC, CHEK2, RAD51D and RAD51*. The WES results were then compared to the patient’s historical molecular report used for enrollment purposes alongside their best RECIST response, as illustrated in Fig. 4.

Our analysis confirmed the pre-identified molecular aberration used for study enrollment in 7 of 9 patients’ (78%) pre-treatment tumor biopsy samples (3 patients enrolled for PTEN loss by IHC were excluded including patients 005, 021 and 030).

### Adverse events

Talazoparib therapy was generally well tolerated with most AEs being grade 1–2. In total, treatment-related AEs occurred in 57/79 (72.2%) patients with the most common being thrombocytopenia (37.9%), anemia (32.9%) fatigue (24.1%) and leukopenia (24.1%) (Supplementary Table 1). Treatment-related AEs that were grade 3–4 occurred in 31/79 (39.2%) patients. Grade 3–4 thrombocytopenia was observed in 17/79 (21.5%) of patients. Fifteen patients (18.9%) experienced grade 3–4 anemia and 12 patients (15.2%) experienced grade 3–4 neutropenia (Supplementary Table 2). All patients who experienced grade 3–4 anemia were transfused with packed red blood cells (PRBCs). Thrombocytopenia and neutropenia were transient and resolved with treatment break. Supplementary Table 3 outlines AEs resulting in dose reduction.

Apart from hematological toxicities, we also encountered one patient with grade 3 fatigue (1.3%), 1 patient with grade 3 diarrhea (1.3%) and 1 patient with grade 3 proteinuria (1.3%). No other grade 3–4 laboratory AEs were encountered during this study. As of August 1, 2022, a total of 40 SAEs were reported during this study, 3 of which were attributed to talazoparib including one patient who died during treatment. This patient was a 67-year-old female with metastatic urothelial cancer who completed only 1 cycle of treatment. She developed grade 3 thrombocytopenia that was attributed to talazoparib, but her death was of unknown cause. The second treatment related SAE was reported for a 51-year-old male with metastatic appendiceal adenocarcinoma who developed grade 4 anemia following 49 days of study treatment. The anemia resolved to baseline after patient received PRBC transfusion. The last treatment related SAE reported was grade 3 diarrhea, which led to discontinuation from study treatment for a 45-year-old male with primary papillary renal cell carcinoma.

Treatment related AEs leading to talazoparib dose modification occurred in 19% of patients. One patient had dose modification due to grade 2 fatigue while the remaining 14 participants were due to hematologic toxicities (Supplementary Table 3).

## Discussion

Recent evidence underscores the versatility of talazoparib, advocating for its use in a variety of contexts, across a spectrum of molecular backgrounds and tumor types. In the era of personalized cancer therapy, where patients are often molecularly profiled at or shortly after the time of cancer relapse, we need to have a better understanding of the biologic and clinical significance of the genomic testing results. We attempted to ascertain the clinical actionability of somatic alterations of *BRCA*1/2, mutations/deletions/amplification in other HR repair pathway genes, *PTEN* genomic alterations or PTEN loss, and germline *BRCA1/2* mutations beyond breast or ovarian cancer, as it pertains to sensitivity to the PARP inhibitor talazoparib.

Patients with deleterious germline *BRCA1/2* mutations have tumors that have demonstrated defects in HR and have been shown in several trials to have response to PARP inhibitors^7,8^. Currently trials have focused on patients with breast and ovarian cancer. We were interested to see if the treatment benefit translated to other tumor types. This phase II study demonstrated encouraging activity of talazoparib in cohorts with somatic and germline *BRCA1/2* mutations, where mean clinical benefit rates (CBRs) were 32.5% and 30.6%, respectively. However, patients with *PTEN* alterations showed limited benefit with mean CBR of 9.4%. Analysis of treatment response by tumor type revealed lower ORR (14.3%) and CBR (28.6%) in our patients compared to previous studies, where ORRs ranging from 31%–33% and CBRs from 48–50% in breast cancers^31^. Despite attempts to stratify by tumor type, our study did not show improvements in ORR and CBR. This outcome could stem from our study’s design, which focused on a range of molecular alterations and deliberately excluded established “responder” disease types such as ovarian and breast cancers in the germline cohort. Our tissue agnostic basket study resulted in varied sample sizes among different tumor types. Additionally, our patient population did not include individuals with known germline *PALB2* mutations at the time of enrollment, a molecular aberration known to have sensitivity to PARP inhibition^32^. Finally, the lower response rate in our study may also be attributable to the extensive prior lines of therapy that our patients had received. This is in line with a previous study of twenty-six breast cancer patients who had undergone up to seven lines of prior chemotherapy, where no objective responses were observed in patients with either *BRCA* mutant or wild-type tumors^33^.

The tolerability of talazoparib in our study aligns with findings from de Bono’s earlier phase I study^34^. Both our study and his predominantly reported anemia, thrombocytopenia, and neutropenia as the most common grade 3 or 4 adverse events (AEs)^34^. Yet, our study observed a higher incidence of thrombocytopenia (Grade 1–2: 37.9% vs. 21%; Grade 3–4: 21.5% vs. 18%), potentially due to our patients undergoing more extensive prior treatments, with a median of 4 systemic therapies compared to 2.5 in de Bono’s study. Contrary to de Bono’s findings, our study reported a lower incidence of alopecia^34^.

In our study, cohort one, focusing on patients with deleterious somatic *BRCA1/2* mutations, showed a mean CBR of 32.5%. Notable responses included a sustained complete response in an ovarian cancer patient with a *BRCA2 R3052W* mutation, partial responses in a soft tissue sarcoma patient (BRCA1 V788fs*10 mutation) and a salivary gland cancer patient (*BRCA1 M1I* mutation), and prolonged partial response in a breast cancer patient (*BRCA1 Q38*0* mutation). Additionally, two patients exhibited stable disease for over 24 weeks. Cohort two, which included patients with alterations in other homologous recombination repair pathway genes, had a CBR of 19.7%. This cohort observed partial responses in patients with appendiceal carcinoma (ATM S1599* mutation), cholangiocarcinoma (*ATM G1676fs* mutation), and urachal adenocarcinoma (PALB2 R170fs14 mutation), along with three cases of prolonged stable disease in patients with mutations in *Fanconi Anemia genes* and *ATM* mutations. These findings are consistent with previous studies that have demonstrated anti-tumor efficacy with PARP inhibition for patients with HRD gene alterations. For example, a patient with advanced pancreatic cancer that responded to talazoparib in the first-in-human phase I study was found to harbor a *PALB2* mutation^34^. Further, in a phase 2 trial of olaparib in patients with advanced, castration-resistant prostate cancer, responses were observed in 4 patients with *ATM* aberrations^35^.

Studies have shown that some cancers with *PTEN* loss are sensitive to PARP inhibitors, and this may be due to down regulation of *Rad51*, a critical HR component^16,17^. This concept has been supported by Shen and colleagues who found that in mouse embryonic fibroblast, inactivation of *PTEN* induced chromosomal instability through defective *Rad51*-mediated HR DNA repair^36^. Additionally, Minami and colleagues found that in *PTEN*-deficient lung cancer cells and xenograft tumor models, combination therapy with cisplatin and olaparib proved to be synergistic^37^. This effect was not seen when *PTEN* was restored in the lung cancer cells or when tested on *PTEN+* xenograft models^37^. Minami and colleagues also found that *PTEN* deficiency led to reductions in nuclear *RAD51*, RPA focus formation and phosphorylated Chk1 and Mre11^37^. Thus, *PTEN* inactivation leads to suppression of DNA repair^37^. However, conflicting data challenges the notion of a direct causal link between *PTEN* and *RAD*51 expression, these studies suggest that *PTEN* may not regulate *Rad51* and that *PTEN* inactivation renders *BRCA 1/2* aberrant cells resistant to PARP^18,19^.

Fourteen patients with *PTEN* mutation/deletion or PTEN loss by IHC were enrolled onto cohort three. The CBR when the cohort enrollment was halted early due to lack of efficacy was 9.4%. We performed a subgroup analysis within the PTEN-altered cohort, differentiating between PTEN deletion or loss by IHC and PTEN inactivating mutations. This stratification yielded a trend towards diminished OS and PFS in both *PTEN* altered subgroups when compared to *PTEN wildtype* and contrasts with previous findings showing that cancers with *PTEN* loss exhibited increased sensitivity to PARP inhibitors^16,17^.

Despite our observations of poorer outcomes in patients with *PTEN*-altered tumors, a 61-year-old breast cancer patient treated in cohort 1 with co-occurring deleterious *BRCA1* and *PTEN* mutations experienced a prolonged PR. It is unclear if the patient’s anti-tumor efficacy is due to the deleterious *BRCA1* mutation and response to PARP inhibition and if this was enough to overcome the trend towards poorer OS and PFS we saw in patients with *PTEN* mutations This case illustrates the complexity and personalized nature of cancer treatment responses and the need for more in depth analysis. Clearly, further studies are needed to determine if *PTEN* alterations are simply a marker of poor prognosis in the spectrum of cancers assessed or if they are true negative predictors for PARP inhibitor therapy benefit. It would also be of interest to determine if the lack-of single agent efficacy could be overcome with combination therapy targeting the PI3K/AKT/mTOR pathway given the *PTEN* mutation status. Interestingly, despite the limitation of a small sample size, Westin and colleagues demonstrated encouraging clinical outcomes in patients with *PTEN* mutation treated with a combination of the PARP and AKT inhibitors, olaparib and capivasertib^38^. Indeed there are emerging preclinical data demonstrating enhanced antitumor activity with the combination of PARP and PI3K pathway inhibitors and PI3K inhibitors were found to have in vivo synergy with PARP inhibitors for the treatment of an endogenous mouse model for *BRCA1*-related breast cancers and patient derived xenograft models of TNBC without *BRCA* mutations^39,40^. Further, there is emerging clinical data suggesting clinical benefit of combinations with PARP and PI3K pathway inhibitors in patients with germline *BRCA* mutations as well as wildtype patients^38,40,41^.

Cohort four, which consisted of diverse tumor types beyond the traditional scope of breast and ovarian cancers, yielded a mean CBR of 30.6%. This expansion of PARP inhibitor use is compelling given the partial response observed in a colorectal cancer patient with a germline BRCA2 p.K944*. Additionally, 4 patients attained SD ≥ 24 weeks including two patients with ampullary and gastric cancers, carrying germline *BRCA2_c.8633-1* *G* > *C* and *BRCA2_Trp2628Ter*, respectively, who exhibited sustained response over a year. These findings echo the successes observed with olaparib in germline *BRCA*-mutated metastatic pancreatic cancer^26^. These findings underscore the therapeutic potential of PARP inhibitors across a range of tumors.

Analyzing the outcomes across various cohorts treated with talazoparib, we observed a disparity in the trend of OS and PFS emerging when comparing hazard ratios between germline *BRCA 1/2* mutated patients (cohort 4) and those with somatic *BRCA 1/2* mutations (cohort 1). The hazard ratio for OS in cohort 4 versus cohort 1 was 1.54 (95% CI: 0.72, 3.27), contrasting with the PFS hazard ratio of 0.91 (95% CI: 0.45, 1.83). This observed discrepancy could be explained by the distinct patient characteristics in cohort 4. Notably, 25% of patients in cohort 4 were diagnosed with pancreatic cancer, known for its aggressive behavior and challenging prognosis. Additionally, there were differences in the clinical characteristics between cohort 1 and 4. Patients in cohort 4 had a median age of 61 years (range 53–72 years), whereas the median age in cohort 1 was 59.5 years (range 32–81 years). Importantly, after discontinuing talazoparib, the median number of treatments received by patients in cohort 1 was 1 (range 0–4), compared to 0.5 (range 0–5) in cohort 4. This suggests that patients in cohort 1 had access to more treatment options post-talazoparib, potentially impacting their survival outcomes.

Our study has several limitations. Firstly, we were limited by the small numbers of patients in each cohort, which may impact the statistical power and generalizability of our findings. Additionally, the patients included in our study were heavily pretreated, having undergone a median of 4 prior systemic therapies. Furthermore, our patient population was heterogenous consisting of various tumor types. The pretreatment history and heterogeneity could have influenced their response to the intervention tested in our study. Another important limitation to consider is that the enrollment criteria were contingent upon molecular aberrations identified in pre-existing molecular reports. This process, while allowing for expedited study enrollment, raises concerns regarding the fidelity of tumor characterization. Moreover, the study did not define a standardized temporal window for molecular testing, which could potentially lead to the inclusion of patients with outdated molecular data.

To address these limitations, it would be beneficial to incorporate real-time comprehensive molecular testing prior to study commencement. This would help ensure a more accurate understanding of the determinates of treatment response and facilitate the identification of reliable biomarkers. Additionally, real-time molecular testing results would help us better match patients to targeted treatments potentially improving the efficacy and precision of our intervention.

In summary, talazoparib was well tolerated by patients and showed promising clinical benefit in a subset of patients with germline as well as somatic alterations in HR repair pathway genes. Further study is needed to determine the efficacy of talazoparib in larger cohorts, and to identify predictive markers of response and resistance that can refine patient selection.

## Methods

### Study design and dosing

This was a prospective study conducted as a single-center, investigator-initiated non-randomized, multi-cohort, phase II study to determine the response of patients with advanced solid tumors who had no curative therapeutic options and pre-identified molecular aberrations appropriate for enrollment to the study. Written Informed consent was obtained and patients were treated in accordance with University of Texas, MD Anderson Cancer Center Institutional Review Board (IRB) guidelines. Treatment with the PARP inhibitor, talazoparib, was administered on an outpatient basis at MD Anderson. Biomarin (and later Pfizer) supplied talazoparib tosylate in 250 µg capsules and subjects in all cohorts were instructed to take 1 mg daily. Compliance was determined through pill diaries and the return of unused drugs at the end of every cycle. Potential toxicities and patient safety were evaluated at baseline, weekly during cycle 1 and before clearance to begin every new 28-day cycle. Toxicities were assessed by adverse events monitoring, physical exams and laboratory tests. Complete blood counts (CBCs) continued to be performed weekly beyond cycle 1 to ensure adequate bone marrow function. Adverse events (AEs) were graded according to the National Cancer Institute (NCI) Common Terminology Criteria for Adverse Events (CTCAE) Version 4.03. Patients who experienced Grade 3 or 4 AEs, resumed drug at 25% dose reduction after recovery with a maximum of 2 dose reductions allowed per patient. Clinical trial information: NCT02286687.

### Ethics approval and consent to participate

All participants signed the informed consent form prior to study enrollment and study related procedures. The study was approved by MD Anderson Institutional Review Board (IRB) in accordance with the Declaration of Helsinki, Good Clinical Practice, and all federal, state and local regulatory guidelines.

### Cohort assignment

Subjects were assigned to one of four cohorts based on pre-identified molecular aberrations determined through a Clinical Laboratory Improvement Amendments (CLIA) validated approach. To be eligible for enrollment, the molecular aberrations had to be predicted to be deleterious, or loss of PTEN expression by gene or immunohistochemistry (IHC). The cohorts included: (1) somatic mutations or deletions of *BRCA*1/2; (2) mutations, homozygous deletions or amplification in other HR repair pathway genes (e.g., *ATM*, *PALB2*, *Fanconi Anemia* genes, *ARID1A*, *MRE11*, *RAD50*, *NBS1*, *ATR* or *EMSY* amplification); (3) mutations or homozygous deletions in *PTEN* and/or PTEN loss by IHC; and (4) germline *BRCA*1/2 mutations (not breast or ovarian cancer). Patients with breast or ovarian cancer were eligible if they did not have germline *BRCA*1/2 mutations but had another alteration qualifying for cohorts 1–3.

Germline testing was done locally as part of standard-of-care for patients on the germline cohort (cohort 4). All other cohorts had germline sequencing in the research environment after informed consent. Patients with somatic mutations in *BRCA1/2* were initiated on treatment prior to germline testing, but germline testing was performed to confirm cohort stratification (i.e., to determine if the *BRCA1/2* mutation was somatic or germline). In Cohort 2 of our study, eligibility for patient enrollment was contingent upon the identification of either germline or somatic pathogenic variants in specific homologous recombination (HR) DNA repair pathway genes, excluding *BRCA1/2*. The genes targeted for Cohort 2 enrollment included *PALB2, EMSY, ATR, ATM, RAD51, BAP1, BARD1*, and various *Fanconi Anemia genes* and others. Based on the specific pathogenic variant detected. Patients with somatic as well as deleterious germline alterations in *PTEN* were eligible for cohort 3. For patients with more than one alteration, they were assigned as follows:

- Patients with germline *BRCA1/2* alterations (not breast or ovarian cancer) were assigned to cohort 4, regardless of other alterations.
- Patients with somatic *BRCA1/2* mutations or deletions were assigned to cohort 1, regardless of other somatic alterations.
- Patients with *PTEN* mutations or deletions or loss of PTEN expression were assigned to cohort 3, in the absence of *BRCA* alterations, and regardless of other HR repair pathway alterations.
- Patients with mutations or deletions in *ATM; PALB2; Fanconi Anemia genes; ARID1A; MRE11; RAD50; NBS1;* and *ATR*; or amplification of EMSY were assigned to cohort 2, in the absence of other *BRCA* alterations.

### Eligibility criteria

Key inclusion criteria for patients meeting the above-outlined requirements included disease that is biopsiable and measurable by RECIST v1.1, Eastern Cooperative Oncology Group (ECOG) performance status (PS) of 0–1, at least 4 weeks beyond last treatment and adequate organ function defined as absolute neutrophil count (ANC) ≥ 1500/mL, platelet count ≥100,000/mL, hemoglobin 9 ≥ g/dL, serum creatinine ≤ 1.5X the upper limit of normal (ULN) (or glomerular filtration rate (GFR) ≥ 60 mL/min), serum total bilirubin ≤ 1.5 x ULN (or direct bilirubin ≤ ULN), or alanine aminotransferase (ALT) and aspartate aminotransferase (AST) ≤ 2.5 x ULN or ≤ 5 x ULN with liver metastases. Key exclusion criteria were prior treatment with a PARP inhibitor, known hepatitis B, hepatitis C or human immunodeficiency virus (HIV) infection, active central nervous system (CNS) metastases and/or carcinomatous meningitis, additional malignancy that is progressing or requires active treatment, clinically significant cardiovascular disease, active infection requiring IV antibiotics or other uncontrolled intercurrent illness requiring hospitalization, inability to swallow and pregnancy/breastfeeding.

### Genomic eligibility

All genomic alterations in eligible genes were reviewed by the MD Anderson Precision Oncology Decision Support (PODS) prior to patient enrolment. Alterations were researched within the published literature for any known effect on function, stability, expression, or therapeutic sensitivity. Alterations were then classified for their functional significance and variant-level actionability, as previously described^42^. Those with a known loss-of-function (Inactivating) or where a loss-of-function can be predicted due to a premature truncation or frameshift prior to well-characterized essential domains (Inactivating: Inferred) were considered eligible for the trial. Comprehensive details regarding the type of sample utilized for molecular testing, sample collection date, molecular report date, study entry date, specific commercial platform used in molecular testing (including panel version), and lines of systematic treatment administered after molecular testing (used for study enrollment) and preceding the initiation of talazoparib treatment are summarized in Supplementary Table 5.

### Assessment of tumor response

Baseline radiographic imaging (e.g., computed tomography (CT) scan or magnetic resonance imaging (MRI)) was performed within four weeks of the start of treatment. Tumor measurements were performed on patients at baseline and at the end of every two cycles (three cycles after 24 weeks). Measurable target lesions were evaluated for response using the Response Evaluation Criteria in Solid Tumors (RECIST v1.1). For purposes of this report, prolonged stable disease (SD) was defined as SD lasting ≥ 24 weeks. Clinical benefit was defined as CR, PR, or SD ≥ 24 weeks.

### PTEN IHC testing

For cohort 3, patients could enroll with PTEN loss on any CLIA test on archival samples. For patients treated on cohort 3, central immunohistochemistry for PTEN was performed on pre-treatment formalin fixed and paraffin embedded (FFPE) core needle biopsies. IHC was performed as previously described^43,44^. Briefly, the staining was performed on 4-micron sections using a monoclonal mouse anti-Human *PTEN* antibody, Clone 6H2.1 (DAKO, Santa Clara, CA, USA) with a 1:100 dilution. Control tissue was stained with the same run. All stains were reviewed by a qualified pathologist blinded to clinical data.

Both intensity and percent positivity of cytoplasmic staining was assessed on the biopsies. Stromal cells and/or endothelial cells with PTEN expression were used as internal positive controls. Any signal arising within the nucleus or from the cell surface membrane was documented but not used for scoring purpose. PTEN expression was semi-quantitatively scored as 0 (no staining), 1 (light staining), 2 (moderate staining) and 3 (strong staining). If there was no staining of tumor cells and no PTEN expression in stroma or endothelial cells the staining was considered as inconclusive, and the result was not considered for analysis.

Histologic score (H-Score) for cytoplasmic staining was calculated by using the following previously published criteria^45^. H-score ranged from 0 (no staining) to 300 (maximum staining) and was calculated by using the formula: H Score = 1x (% light staining) + 2x (% moderate staining) + 3x (% strong staining).

### Whole exome sequencing of tumor and analysis

Pre-treatment tumor biopsies were obtained from 12 patients in cohort 3 (*PTEN* alterations and PTEN loss by IHC), from which DNA was extracted and purified. Whole-exome libraries were constructed using biotin-labeled probes from the SeqCap EZ Exome V3 (Roche, Pleasanton, CA, USA) and subsequently sequenced on an Illumina HiSeq4000 sequencer (Illumina Inc., San Diego, CA, USA). Sequence reads were aligned to the human reference genome Hg19 using BWA, with somatic variants identified using MuTect^46^, germline mutations called using Platypus^47^, and indels detected using Pindel^48^. Alignment of FASTQ files to the reference genome utilized specific parameters with BWA^49^, and preprocessing steps including duplicate marking, realignment, and recalibration were carried out using Picard and GATK^50^. DNA copy number analysis employed HMMcopy for whole genome sequencing data and an in-house application, ExomeLyzer^51^, for whole exon sequencing data, with segmentation performed using CBS^52^. WES variant calls list is provided in Supplementary Note 1.

### Statistical considerations and analysis

The study employed Simon’s Two-stage design^53^, which incorporates early stopping rules for futility. Initially, a minimum of 10 evaluable patients were enrolled onto each cohort and assessed for clinical benefit during the first stage. If a cohort met the predefined criteria for >25% clinical benefit rate, the study progressed to the second stage, wherein additional 20 patients were enrolled. The maximum sample size to each cohort was set at 30, except for cohort 2 (other HR pathways, which permitted enrollment up to 30 patients for each sub-cohort). The *PTEN* cohort was terminated early due lack of response.

The primary endpoint of this study was clinical benefit rate (CBR) in each cohort. Clinical benefit (CB) is defined as any of the following, CR, PR, or SD for ≥ 24 weeks. A Bayesian design was used to calculate efficacy (targeting a clinical benefit rate of 25%, 90% credible interval:14–38%) and monitor toxicity^54^. If the lower bound of the 90% posterior credible interval is ≥14%, the drug will be recommended for a subsequent independent confirmatory study.

CBR was assessed using posterior probabilities along with 90% credible interval. Objective response rate (ORR) with 95% confidence interval (CI) was calculated using exact binomial method. Secondary endpoints included progression-free survival (PFS), overall survival (OS), duration of response (DOR) and toxicities. PFS was defined as from treatment start to progression or death, whichever occurred first, or last follow up. OS was defined as from treatment start to death or last follow up. DOR was defined as time from response to progression, death or last follow up among the objective responders (CR or PR). Toxicities were summarized by frequency. Distributions of PFS and OS were estimated using the Kaplan Meier method^55^. The differences in PFS or OS were estimated using the Cox proportional hazards regression model.

### Reporting summary

Further information on research design is available in the Nature Research Reporting Summary linked to this article.

### Supplementary information

Supplementary Information

Editorial policy checklist

Author list change memo

Reporting summary

## Data Availability

The whole-exome sequencing data generated and analyzed in this study have been deposited at MD Anderson OpenWorks repository, accessible via 10.52519/00133, under controlled access as per MD Anderson compliance guideline^56^. These data may be subject to patient confidentiality and may require a material transfer agreement. The de-identified participant data and dataset generated during and/or analyzed during the current study are available from the corresponding author on reasonable request.

## Code availability

The R code used to generate heatmap is provided in Supplementary Note 2.
