# Supplementary material for: Phase II study of talazoparib in advanced cancers with BRCA1/2, DNA repair, and PTEN alterations
Source: NPJ Precis Oncol. 2024 Jul 31;8:166. doi: 10.1038/s41698-024-00634-6 (PMC11291882; doi:10.1038/s41698-024-00634-6)
Supplement: Supplementary file 1 — Supplementary Information [file 41698_2024_634_MOESM1_ESM.pdf]

Supplementary Figure 1: Consort Flow Diagram

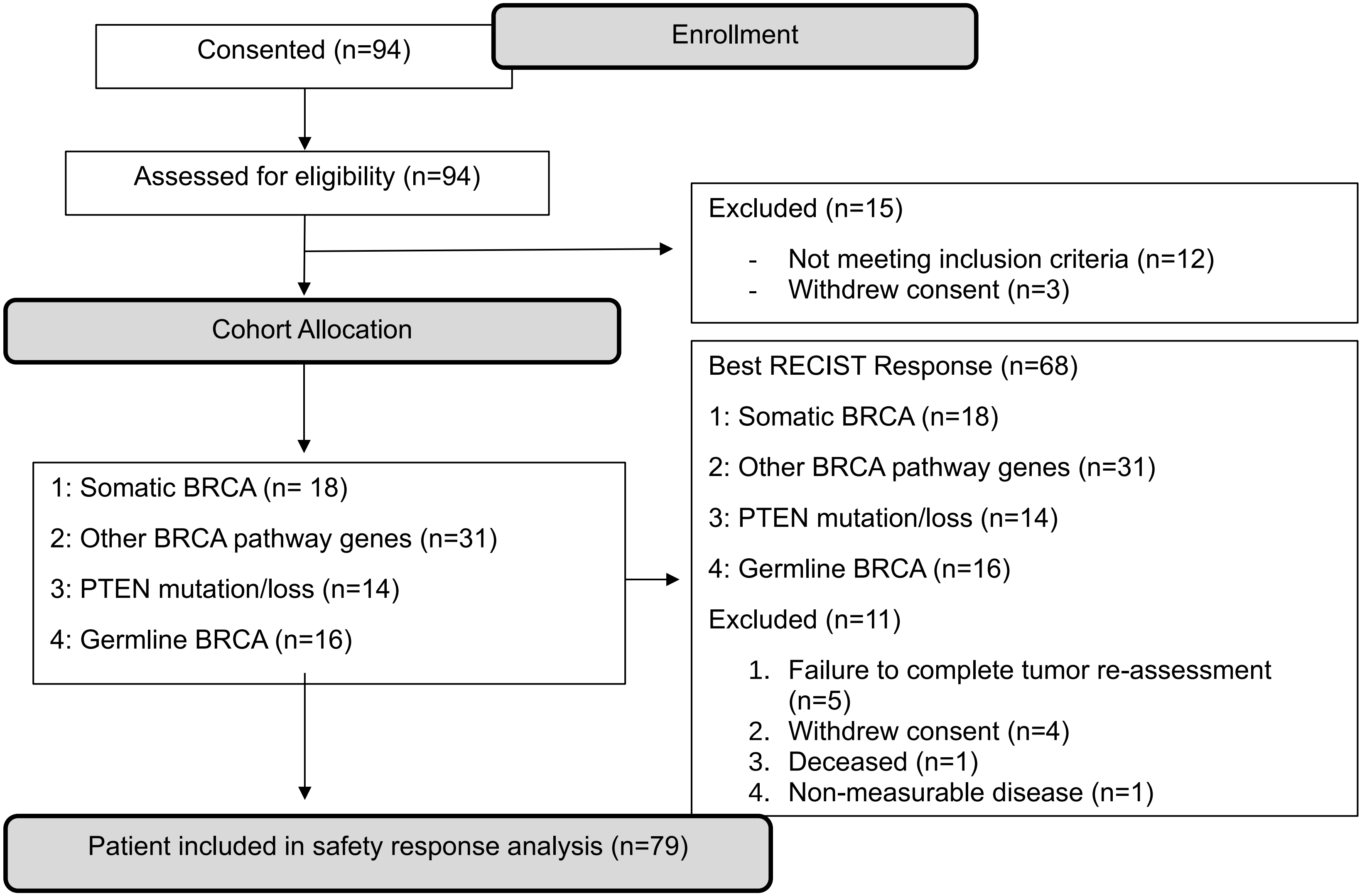

Abbreviation: PTEN, Phosphatase and Tensin homolog; BRCA, Breast Cancer gene; n, number.

Supplementary Figure 1: Consort Flow Diagram

Consort flow diagram of enrollment to the study.

Supplementary Table 1: Treatment related adverse events (TRAEs)

	Cohort				
	1	2	3	4	
Adverse Event of All Grades*	**Somatic *BRCA1/2* Mutation N=18**	**Other HR repair pathway gene aberrations¶ N=31**	***PTEN* Mutation or Loss by IHC N=14**	**Germline *BRCA1/2* Mutation N=16**	**All Participants (N=79)**
**Participants with at least one TRAE**	12 (66.7)	24 (77.4)	10 (71.4)	11 (68.8)	57 (72.2)
Proteinuria	0	1 (3.2)	0	0	1 (1.3)
Anemia	6 (33.3)	8 (25.8)	7 (50.0)	5 (31.3)	26 (32.9)
Constipation	1 (5.6)	1 (3.2)	1 (7.1)	0	3 (3.8)
Diarrhea	0	3 (9.7)	0	1 (6.3)	3 (3.8)
Fatigue	1 (5.6)	8 (25.8)	5 (35.7)	5 (31.3)	19 (24.1)
Neutropenia	6 (33.3)	6 (19.4)	2 (14.3)	4 (25.0)	18 (22.8)
Nausea	0	6 (19.4)	2 (14.3)	2 (12.5)	10 (12.7)
Thrombocytopenia	6 (33.3)	9 (29.0)	7 (50.0)	8 (50.0)	30 (37.9)
Elevated ALT	0	2 (6.4)	0	0	2 (2.5)
Leukopenia	7 (38.9)	6 (19.4)	3 (21.4)	3 (18.8)	19 (24.1)
Mucositis	1 (5.6)	1 (3.2)	2 (14.3)	0	4 (5.1)
Cramping in lower calves	1 (5.6)	0	0	0	1 (1.3)
Gum sensitivity	1 (5.6)	0	0	0	1 (1.3)
Alopecia	0	0	0	1 (6.3)	1 (1.3)
Rash	0	1 (3.2)	1 (7.1)	2 (12.5)	4 (5.1)
Anorexia	0	2 (6.4)	1 (7.1)	2 (12.5)	5 (6.3)
Vomiting	0	3 (9.7)	0	2 (12.5)	5 (6.3)

Abdominal pain	0	1 (3.2)	0	0	1 (1.3)
Bloating	0	1 (3.2)	0	0	1 (1.3)
Headache	0	1 (3.2)	0	0	1 (1.3)
Hypomagnesemia	0	1 (3.2)	0	0	1 (1.3)
Musculoskeletal and connective tissue disorder	0	1 (3.2)	0	0	1 (1.3)
Myalgia	0	1 (3.2)	0	0	1 (1.3)

*Adverse events deemed at least possibly related to treatment were graded based on the Common Terminology Criteria for Adverse Events, Version 4 (CTCAE 4.0). ¶ Includes mutations, deletions and amplifications. Abbreviations: ALT, alanine aminotransferase; BRCA, Breast Cancer gene; N, number; IHC, immunohistochemistry; PTEN, Phosphatase and Tensin homolog.

Supplementary Table 2: Grade 3-4 TRAEs that occurred in >1 participant by preferred term

	Cohort				
	1	2	3	4	
Adverse Event of All Grades	**Somatic *BRCA1/2* Mutation N=18**	**Other HR repair pathway gene aberrations¶ N=31**	***PTEN* Mutation or Loss by IHC N=14**	**Germline *BRCA1/2* Mutation N=16**	**All Participants (N=79)**
**Participants with at least one TRAE**	5 (27.8)	12 (38.7)	7 (50.0)	7 (43.8)	31 (39.2)
Anemia	3 (16.7)	5 (16.1)	3 (21.4)	4 (25.0)	15 (18.9)
Diarrhea	0	1 (3.2)	0	0	1 (1.3)
Fatigue	0	0	1 (7.1)	0	1 (1.3)
Neutropenia	4 (22.2)	4 (12.9)	1 (7.1)	3 (18.8)	12 (15.2)
Thrombocytopenia	1 (5.6)	7 (22.6)	4 (28.6)	5 (31.3)	17 (21.5)
Proteinuria	0	1 (3.2)	0	0	1 (1.3)
Leukopenia	1 (5.6)	3 (9.6)	0	1 (6.3)	5 (6.3)

¶ Includes mutations, deletions and amplifications. Abbreviations: BRCA, Breast Cancer gene; N, number; IHC, immunohistochemistry; PTEN, Phosphatase and Tensin homolog, TEAE, treatment related adverse event.

Supplementary Table 3: Treatment related adverse events (TRAEs) that required dose reduction

Cohort	Somatic *BRCA1/2* Mutation N=18	Other HR repair pathway gene aberrations¶ N=31	*PTEN* Mutation or Loss by IHC N=14	Germline *BRCA1/2* Mutation N=16	All Participants (N=79)
**TRAEs that required dose reduction**	Neutropenia-G3 (2)*<br>Anemia-G3 (1)*	Anemia-G3 (2)*<br>Neutropenia-G3/ Thrombocytopenia-G3 (1)*<br>Thrombocytopenia-G3 (1)*	Thrombocytopenia-G3 (1)*	Anemia-G3 (1)*<br>Fatgiue-G2 (1)*<br>Neutropenia-G3 (1) *<br>Thrombocytopenia-G3/4 (2)*<br>Anemia-G3/Neutropenia-G3/4 (2)*	15 (19)^

^ indicate percentage. * Number of events ¶ Includes mutations, deletions and amplifications. Abbreviations: *BRCA, Breast Cancer gene*; N, number; IHC, immunohistochemistry; PTEN, Phosphatase and Tensin homolog; G, grade.

Supplementary Table 4: Objective response rate and clinical benefit rate by tumor type

Tumor Type	Number (%)	ORR (%) (CR+PR)	CBR (%)	Prior treatment Median (range)
Sarcoma^	11 (13.9)	1 (9.1)	3 (27.3)	4 (3-11)
Colorectal	10 (12.7)	1 (10)	1 (10)	3.5 (2-12)
Breast	7 (8.9)	1 (14.3)	2 (28.6)	4 (3-13)
Cholangiocarcinoma	7 (8.9)	1 (14.3)	2 (28.6)	4 (2-10)
Ovarian	6 (7.6)	1 (16.7)	2 (33.3)	4 (2-13)
Pancreas	6 (7.6)	0	1 (16.7)	3 (1-6)
Head and neck	5 (6.3)	0	0	5 (3-9)
Bladder	3 (3.8)	0	0	4 (1-6)
Ampulla of Vater	3 (3.8)	0	1 (33.3)	2 (2-3)
Gastric	3 (3.8)	0	2 (66.7)	4 (3-4)
Renal	3 (3.8)	0	0	4 (2-5)
Endometrial	2 (2.5)	0	0	3 (3)
Gall bladder	2 (2.5)	0	1 (50)	4 (2-6)
Lung	2 (2.5)	0	0	2.5 (2-3)
Salivary gland	1 (1.3)	1 (100)	1 (100)	2
Urachal	1 (1.3)	1 (100)	1 (100)	3
Melanoma	1 (1.3)	0	0	1
Appendiceal	1 (1.3)	1 (100)	1 (100)	2
Cervical	1 (1.3)	0	0	2
Esophageal	1 (1.3)	0	0	2
Squamous cell carcinoma of skin	1 (1.3)	0	0	2

Sebaceous adenocarcinoma on upper eyelid	1 (1.3)	0	0	3
Unknown primary	1 (1.3)	0	0	1

Abbreviation: ORR, objective response rate; CR, complete response; PR, partial response; CBR, clinical benefit rate.

Supplementary Table 5: Molecular Aberrations

Study ID	Path DiseaseType	Alteration	Panel Name	Panel_Version	Molecular test sample	sample collection date	ResultDate	Study Entry	Lines of therapies [@]
01-025	Desmoplastic small round cell tumor	BRCA2_D1737fs*4	FoundationOne Heme	FoundationOne Heme_406/31/265	Surgical resected -Colon	9/30/2014	11/7/2014	8/21/2015	1
01-025	Desmoplastic small round cell tumor	CD70_*194fs*1+	FoundationOne Heme	FoundationOne Heme_406/31/265	Surgical resected -Colon	9/30/2014	11/7/2014	8/21/2015	
01-025	Desmoplastic small round cell tumor	AR_G457_G463del	FoundationOne Heme	FoundationOne Heme_406/31/265	Surgical resected -Colon	9/30/2014	11/7/2014	8/21/2015	
01-025	Desmoplastic small round cell tumor	ATR_H1440R	FoundationOne Heme	FoundationOne Heme_406/31/265	Surgical resected -Colon	9/30/2014	11/7/2014	8/21/2015	
01-025	Desmoplastic small round cell tumor	CSF1R_V32G	FoundationOne Heme	FoundationOne Heme_406/31/265	Surgical resected -Colon	9/30/2014	11/7/2014	8/21/2015	
01-025	Desmoplastic small round cell tumor	KRAS_Q70fs*3	FoundationOne Heme	FoundationOne Heme_406/31/265	Surgical resected -Colon	9/30/2014	11/7/2014	8/21/2015	
01-025	Desmoplastic small round cell tumor	MAP2K2_V215L	FoundationOne Heme	FoundationOne Heme_406/31/265	Surgical resected -Colon	9/30/2014	11/7/2014	8/21/2015	
01-025	Desmoplastic small round cell tumor	MED12_Q2105_Q2107>Q	FoundationOne Heme	FoundationOne Heme_406/31/265	Surgical resected -Colon	9/30/2014	11/7/2014	8/21/2015	
01-025	Desmoplastic small round cell tumor	MIB1_G916R	FoundationOne Heme	FoundationOne Heme_406/31/265	Surgical resected -Colon	9/30/2014	11/7/2014	8/21/2015	
01-025	Desmoplastic small round cell tumor	PTPN11_T553M	FoundationOne Heme	FoundationOne Heme_406/31/265	Surgical resected -Colon	9/30/2014	11/7/2014	8/21/2015	
01-025	Desmoplastic small round cell tumor	PTPRO_R877C	FoundationOne Heme	FoundationOne Heme_406/31/265	Surgical resected -Colon	9/30/2014	11/7/2014	8/21/2015	
01-025	Desmoplastic small round cell tumor	EWSR1-WT1	FoundationOne Heme	FoundationOne Heme_406/31/265	Surgical resected -Colon	9/30/2014	11/7/2014	8/21/2015	
01-025	Desmoplastic small round cell tumor	TP53_Deletion	FoundationOne Heme	FoundationOne Heme_406/31/265	Surgical resected -Colon	9/30/2014	11/7/2014	8/21/2015	
01-038	Adenocarcinoma of Lung	MLL_R2208W	CMS28	CMS28_11042016	unknown	5/27/2015	6/4/2015	3/23/2018	
01-038	Adenocarcinoma of Lung	FLT3_S684P	CMS28	CMS28_11042016	unknown	5/27/2015	6/4/2015	3/23/2018	
01-038	Adenocarcinoma of Lung	TSC1_R718W	STGAv1	STGAv1_03132018	Tumor biopsy	4/11/2017	5/4/2017	3/23/2018	
01-038	Adenocarcinoma of Lung	KRAS_G12A	STGAv1	STGAv1_03132018	Tumor biopsy	4/11/2017	5/4/2017	3/23/2018	
01-038	Adenocarcinoma of Lung	TP53_c.673-1G>T	STGAv1	STGAv1_03132018	Tumor biopsy	4/11/2017	5/4/2017	3/23/2018	
01-038	Adenocarcinoma of Lung	BRCA2_S2186fs*3	STGAv1	STGAv1_03132018	Tumor biopsy	4/11/2017	5/4/2017	3/23/2018	0
01-038	Adenocarcinoma of Lung	EGFR_A597S	STGAv1	STGAv1_03132018	Tumor biopsy	4/11/2017	5/4/2017	3/23/2018	
01-038	Adenocarcinoma of Lung	NF1_R1830L	STGAv1	STGAv1_03132018	Tumor biopsy	4/11/2017	5/4/2017	3/23/2018	
01-038	Adenocarcinoma of Lung	PIK3R1_V589fs*13	STGAv1	STGAv1_03132018	Tumor biopsy	4/11/2017	5/4/2017	3/23/2018	
01-038	Adenocarcinoma of Lung	KMT2A_R2208W	LMPv1	EndLeukemia Mutation Panel V1_11052021	Bone marrow	2/7/2018	2/14/2018	3/23/2018	
01-038	Adenocarcinoma of Lung	FLT3_S684P	LMPv1	EndLeukemia Mutation Panel V1_11052021	Bone marrow	2/7/2018	2/14/2018	3/23/2018	
01-038	Adenocarcinoma of Lung	BCORL1_G209S	LMPv1	EndLeukemia Mutation Panel V1_11052021	Bone marrow	2/7/2018	2/14/2018	3/23/2018	
01-038	Adenocarcinoma of Lung	GFI1_S36N	LMPv1	EndLeukemia Mutation Panel V1_11052021	Bone marrow	2/7/2018	2/14/2018	3/23/2018	
01-038	Adenocarcinoma of Lung	MET_N375S	CMS50	CMS50_01042017	Left upper lobe stump	10/16/2013	12/13/2013	3/23/2018	
01-038	Adenocarcinoma of Lung	EGFR_A597S	CMS50	CMS50_01042017	Left upper lobe stump	10/16/2013	12/13/2013	3/23/2018	
01-040	Leiomyosarcoma of uterus	TP53_c.994-1G>A	STGAv1	STGAv1_03132018	Liver	10/06/2015	6/22/2017	4/18/2018	
01-040	Leiomyosarcoma of uterus	BRCA2 S2186fs*3	STGAv1	STGAv1 03132018	Liver	10/06/2015	6/22/2017	4/18/2018	3
01-040	Leiomyosarcoma of uterus	PIK3R1_V589fs*13	STGAv1	STGAv1_03132018	Liver	10/06/2015	6/22/2017	4/18/2018	
01-040	Leiomyosarcoma of uterus	CALR_E398_D400del	LMPv1	EndLeukemia Mutation Panel V1_11052021	Bone marrow	10/9/2018	10/16/2018	4/18/2018	
01-040	Leiomyosarcoma of uterus	TET2_H1778R	LMPv1	EndLeukemia Mutation Panel V1_11052021	Bone marrow	10/9/2018	10/16/2018	4/18/2018	
01-040	Leiomyosarcoma of uterus	SH2B3_T165S	LMPv1	EndLeukemia Mutation Panel V1_11052021	Bone marrow	10/9/2018	10/16/2018	4/18/2018	
01-040	Leiomyosarcoma of uterus	ASXL1_L1325F	LMPv1	EndLeukemia Mutation Panel V1_11052021	Bone marrow	10/9/2018	10/16/2018	4/18/2018	
01-040	Leiomyosarcoma of uterus	EZH2_D185H	LMPv1	EndLeukemia Mutation Panel V1_11052021	Bone marrow	10/9/2018	10/16/2018	4/18/2018	
01-040	Leiomyosarcoma of uterus	TET2_V218M	LMPv1	EndLeukemia Mutation Panel V1_11052021	Bone marrow	10/9/2018	10/16/2018	4/18/2018	
01-040	Leiomyosarcoma of uterus	ASXL1_E1102D	LMPv1	EndLeukemia Mutation Panel V1_11052021	Bone marrow	10/9/2018	10/16/2018	4/18/2018	
01-040	Leiomyosarcoma of uterus	FLT3_V557I	LMPv1	EndLeukemia Mutation Panel V1_11052021	Bone marrow	10/9/2018	10/16/2018	4/18/2018	
01-040	Leiomyosarcoma of uterus	STAT5A_V209A	LMPv1	EndLeukemia Mutation Panel V1_11052021	Bone marrow	10/9/2018	10/16/2018	4/18/2018	
01-040	Leiomyosarcoma of uterus	TERC_c.58G>A	LMPv1	EndLeukemia Mutation Panel V1_11052021	Bone marrow	10/9/2018	10/16/2018	4/18/2018	
01-048	soft tissue sarcoma	BRCA1 V788fs*10	STGA-DNA 2018	STGA-DNA 2018 11012021	Tumor biopsy	10/3/2016	5/11/2018	6/19/2018	4
01-060	Gastric/Stomach (stomach adenocarcinoma, nos)	STK11_F354L	CMS50	CMS50_01042017	Tumor biopsy-stomach	12/9/2016	11/9/2017	9/28/2018	
01-060	Gastric/Stomach (stomach adenocarcinoma, nos)	TP53_R196*	CMS50	CMS50_01042017	Tumor biopsy-stomach	12/9/2016	11/9/2017	9/28/2018	
01-060	Gastric/Stomach (stomach adenocarcinoma, nos)	TP53_R196*	Guardant360	Guardant360_73Gene Nov2016 - Mar2019	Blood	12/28/2017	1/8/2018	9/28/2018	
01-060	Gastric/Stomach (stomach adenocarcinoma, nos)	BRCA2_F376fs*23	Guardant360	Guardant360_73Gene Nov2016 - Mar2019	Blood	12/28/2017	1/8/2018	9/28/2018	1
01-060	Gastric/Stomach (stomach adenocarcinoma, nos)	TP53_R196*	STGAv1	STGAv1_03132018	Ovary/Fallopian tube	11/22/2017	1/16/2018	9/28/2018	
01-061	Ovarian	GNAS_R201H	Guardant360	Guardant360_73Gene Nov2016 - Mar2019	Blood	7/11/2017	7/20/2017	10/3/2018	
01-061	Ovarian	TP53_V157F	Guardant360	Guardant360_73Gene Nov2016 - Mar2019	Blood	7/11/2017	7/20/2017	10/3/2018	
01-061	Ovarian	BRCA2_R3052W	Guardant360	Guardant360_73Gene Nov2016 - Mar2019	Blood	7/11/2017	7/20/2017	10/3/2018	1
01-061	Ovarian	NF1_c.4332+2T>G	Guardant360	Guardant360_73Gene Nov2016 - Mar2019	Blood	7/11/2017	7/20/2017	10/3/2018	
01-061	Ovarian	TP53_V157F	STGA-DNA 2018	STGA-DNA 2018_11012021	OVARY	6/16/2015	8/24/2018	10/3/2018	
01-061	Ovarian	BRCA2_R3052W	STGA-DNA 2018	STGA-DNA 2018_11012021	OVARY	6/16/2015	8/24/2018	10/3/2018	
01-061	Ovarian	TET2_L1375*	LMPv1	EndLeukemia Mutation Panel V1_11052021	Bone marrow	3/12/2019	3/15/2019	10/3/2018	
01-061	Ovarian	GATA2_P161A	LMPv1	EndLeukemia Mutation Panel V1_11052021	Bone marrow	3/12/2019	3/15/2019	10/3/2018	
01-061	Ovarian	NF1_c.4332+2T>G	LMPv1	EndLeukemia Mutation Panel V1_11052021	Bone marrow	3/12/2019	3/15/2019	10/3/2018	
01-061	Ovarian	BCORL1_G209S	LMPv1	EndLeukemia Mutation Panel V1_11052021	Bone marrow	3/12/2019	3/15/2019	10/3/2018	
01-061	Ovarian	CBLC_P435S	LMPv1	EndLeukemia Mutation Panel V1_11052021	Bone marrow	3/12/2019	3/15/2019	10/3/2018	
01-071	Colorectal	TP53_I195fs*52	Liquid Biopsy Panel V1	Liquid Biopsy Panel V1_11072021	Plasm circulating cell-free DNA	2/5/2019	2/12/2019	3/13/2019	
01-071	Colorectal	BRCA1_G1062S	Liquid Biopsy Panel V1	Liquid Biopsy Panel V1_11072021	Plasm circulating cell-free DNA	2/5/2019	2/12/2019	3/13/2019	
01-071	Colorectal	BRCA2_C1809*	Liquid Biopsy Panel V1	Liquid Biopsy Panel V1_11072021	Plasm circulating cell-free DNA	2/5/2019	2/12/2019	3/13/2019	0
01-071	Colorectal	KRAS_G12V	Liquid Biopsy Panel V1	Liquid Biopsy Panel V1_11072021	Plasm circulating cell-free DNA	2/5/2019	2/12/2019	3/13/2019	
01-071	Colorectal	APC_E1353*	Liquid Biopsy Panel V1	Liquid Biopsy Panel V1_11072021	Plasm circulating cell-free DNA	2/5/2019	2/12/2019	3/13/2019	
01-073	Breast (breast carcinoma (nos))	ESR1_Y537S	FoundationOne	FoundationOne_315/28 Aug 2014 - Dec 2017	Lung	1/29/2015	2/17/2015	4/4/2019	
01-073	Breast (breast carcinoma (nos))	MSH2_T292S	FoundationOne	FoundationOne_315/28 Aug 2014 - Dec 2017	Lung	1/29/2015	2/17/2015	4/4/2019	
01-073	Breast (breast carcinoma (nos))	NOTCH2_A3F	FoundationOne	FoundationOne_315/28 Aug 2014 - Dec 2017	Lung	1/29/2015	2/17/2015	4/4/2019	
01-073	Breast (breast carcinoma (nos))	BLM_E1143K	FoundationOne	FoundationOne_315/28 Aug 2014 - Dec 2017	Lung	1/29/2015	2/17/2015	4/4/2019	
01-073	Breast (breast carcinoma (nos))	ATM_Deletion_Exons_57-63	FoundationOne	FoundationOne_315/28 Aug 2014 - Dec 2017	Lung	1/29/2015	2/17/2015	4/4/2019	
01-073	Breast (breast carcinoma (nos))	TERT_H412Y	LMPv1	EndLeukemia Mutation Panel V1_11052021	Bone marrow	9/28/2018	10/3/2018	4/4/2019	
01-073	Breast (breast carcinoma (nos))	CCND1_Amplification	FoundationOne	FoundationOne_315/28 Aug 2014 - Dec 2017	Lung	1/29/2015	2/17/2015	4/4/2019	
01-073	Breast (breast carcinoma (nos))	EMSY_Amplification	FoundationOne	FoundationOne_315/28 Aug 2014 - Dec 2017	Lung	1/29/2015	2/17/2015	4/4/2019	
01-073	Breast (breast carcinoma (nos))	FGF19_Amplification	FoundationOne	FoundationOne_315/28 Aug 2014 - Dec 2017	Lung	1/29/2015	2/17/2015	4/4/2019	
01-073	Breast (breast carcinoma (nos))	FGF3_Amplification	FoundationOne	FoundationOne_315/28 Aug 2014 - Dec 2017	Lung	1/29/2015	2/17/2015	4/4/2019	
01-073	Breast (breast carcinoma (nos))	FGF3_Amplification	STGA-DNA 2018	STGA-DNA 2018_11012021	Tumor biopsy-Pleura	4/19/2021	5/12/2021	4/4/2019	
01-073	Breast (breast carcinoma (nos))	FGF4_Amplification	FoundationOne	FoundationOne_315/28 Aug 2014 - Dec 2017	Lung	1/29/2015	2/17/2015	4/4/2019	
01-073	Breast (breast carcinoma (nos))	MCL1_Amplification	FoundationOne	FoundationOne_315/28 Aug 2014 - Dec 2017	Lung	1/29/2015	2/17/2015	4/4/2019	
01-073	Breast (metastatic breast adenocarcinoma)	TP53_A159V	Liquid Biopsy Panel V1	Liquid Biopsy Panel V1_11072021	Plasm circulating cell-free DNA	10/15/2018	10/25/2018	4/4/2019	
01-073	Breast (metastatic breast adenocarcinoma)	TP53_P152L	Liquid Biopsy Panel V1	Liquid Biopsy Panel V1_11072021	Plasm circulating cell-free DNA	10/15/2018	10/25/2018	4/4/2019	
01-073	Breast (metastatic breast adenocarcinoma)	ESR1_Y537S	Liquid Biopsy Panel V1	Liquid Biopsy Panel V1_11072021	Plasm circulating cell-free DNA	10/15/2018	10/25/2018	4/4/2019	
01-073	Breast (metastatic breast adenocarcinoma)	ATM_M3011T	Liquid Biopsy Panel V1	Liquid Biopsy Panel V1_11072021	Plasm circulating cell-free DNA	10/15/2018	10/25/2018	4/4/2019	
01-073	Breast (metastatic breast adenocarcinoma)	BRCA1_Q356*	Liquid Biopsy Panel V1	Liquid Biopsy Panel V1_11072021	Plasm circulating cell-free DNA	10/15/2018	10/25/2018	4/4/2019	0
01-073	Breast (breast duct carcinoma)	CDK12_F336I	STGA-DNA 2018	STGA-DNA 2018_11012021	Tumor biopsy-Pleura	4/19/2021	5/12/2021	4/4/2019	
01-073	Breast (breast duct carcinoma)	ESR1_Y537S	STGA-DNA 2018	STGA-DNA 2018_11012021	Tumor biopsy-Pleura	4/19/2021	5/12/2021	4/4/2019	
01-074	Ovarian serous adenocarcinoma	ATM_S333F	CMS50	CMS50_01042017	Tumor biopsy-subcutaneous nodule	10/22/2013	2/10/2017	5/13/2019	
01-074	Ovarian serous adenocarcinoma	KIT_M541L	CMS50	CMS50_01042017	Tumor biopsy-subcutaneous nodule	10/22/2013	2/10/2017	5/13/2019	
01-074	Ovarian serous adenocarcinoma	KRAS_G12D	CMS50	CMS50_01042017	Tumor biopsy-subcutaneous nodule	10/22/2013	2/10/2017	5/13/2019	
01-074	Ovarian serous adenocarcinoma	PIK3CA_I391M	CMS50	CMS50_01042017	Tumor biopsy-subcutaneous nodule	10/22/2013	2/10/2017	5/13/2019	
01-074	Ovarian serous adenocarcinoma	ABL1_G626S	FoundationOne	FoundationOne_315/28 Aug 2014 - Dec 2017	Soft Tissue	1/10/2017	2/10/2017	5/13/2019	
01-074	Ovarian serous adenocarcinoma	ATM_S333F	FoundationOne	FoundationOne_315/28 Aug 2014 - Dec 2017	Soft Tissue	1/10/2017	2/10/2017	5/13/2019	
01-074	Ovarian serous adenocarcinoma	ERBB2_T245M	FoundationOne	FoundationOne_315/28 Aug 2014 - Dec 2017	Soft Tissue	1/10/2017	2/10/2017	5/13/2019	
01-074	Ovarian serous adenocarcinoma	KRAS_G12D	FoundationOne	FoundationOne_315/28 Aug 2014 - Dec 2017	Soft Tissue	1/10/2017	2/10/2017	5/13/2019	
01-074	Ovarian serous adenocarcinoma	MLH1_K618A	FoundationOne	FoundationOne_315/28 Aug 2014 - Dec 2017	Soft Tissue	1/10/2017	2/10/2017	5/13/2019	
01-074	Ovarian serous adenocarcinoma	ATRX_I1132T	FoundationOne	FoundationOne_315/28 Aug 2014 - Dec 2017	Soft Tissue	1/10/2017	2/10/2017	5/13/2019	
01-074	Ovarian serous adenocarcinoma	FOXL2_M264T	FoundationOne	FoundationOne_315/28 Aug 2014 - Dec 2017	Soft Tissue	1/10/2017	2/10/2017	5/13/2019	
01-074	Ovarian serous adenocarcinoma	LRP1B_G1181E	FoundationOne	FoundationOne_315/28 Aug 2014 - Dec 2017	Soft Tissue	1/10/2017	2/10/2017	5/13/2019	
01-074	Ovarian serous adenocarcinoma	SPTA1_L1807S	FoundationOne	FoundationOne_315/28 Aug 2014 - Dec 2017	Soft Tissue	1/10/2017	2/10/2017	5/13/2019	
01-074	Ovarian serous adenocarcinoma	TSC1_G1035S	FoundationOne	FoundationOne_315/28 Aug 2014 - Dec 2017	Soft Tissue	1/10/2017	2/10/2017	5/13/2019	
01-074	Ovarian serous adenocarcinoma	KDM5C_R951Q	FoundationOne	FoundationOne_315/28 Aug 2014 - Dec 2017	Soft Tissue	1/10/2017	2/10/2017	5/13/2019	
01-074	Ovarian serous adenocarcinoma	DICER1_K1913R	FoundationOne	FoundationOne_315/28 Aug 2014 - Dec 2017	Soft Tissue	1/10/2017	2/10/2017	5/13/2019	
01-074	Ovarian serous adenocarcinoma	PIK3C2B_G136R	FoundationOne	FoundationOne_315/28 Aug 2014 - Dec 2017	Soft Tissue	1/10/2017	2/10/2017	5/13/2019	
01-074	Ovarian serous adenocarcinoma	IRF2_D153A	FoundationOne	FoundationOne_315/28 Aug 2014 - Dec 2017	Soft Tissue	1/10/2017	2/10/2017	5/13/2019	
01-074	Ovarian serous adenocarcinoma	EGFR_G724S	Guardant360	Guardant360_73Gene Nov2016 - Mar2019	Blood	10/30/2017	11/10/2017	5/13/2019	
01-074	Ovarian serous adenocarcinoma	ERBB2_Y590C	Guardant360	Guardant360_73Gene Nov2016 - Mar2019	Blood	10/30/2017	11/10/2017	5/13/2019	
01-074	Ovarian serous adenocarcinoma	RET_G849Y	Guardant360	Guardant360_73Gene Nov2016 - Mar2019	Blood	10/30/2017	11/10/2017	5/13/2019	
01-074	Ovarian serous adenocarcinoma	TP53_E224D	Guardant360	Guardant360_73Gene Nov2016 - Mar2019	Blood	10/30/2017	11/10/2017	5/13/2019	
01-074	Ovarian serous adenocarcinoma	TP53_F113del	Guardant360	Guardant360_73Gene Nov2016 - Mar2019	Blood	10/30/2017	11/10/2017	5/13/2019	
01-074	Ovarian serous adenocarcinoma	TP53_P278L	Guardant360	Guardant360_73Gene Nov2016 - Mar2019	Blood	10/30/2017	11/10/2017	5/13/2019	
01-074	Ovarian serous adenocarcinoma	TP53_P177R	Guardant360	Guardant360_73Gene Nov2016 - Mar2019	Blood	10/30/2017	11/10/2017	5/13/2019	
01-074	Ovarian serous adenocarcinoma	BRCA2_N1435fs*13	Guardant360	Guardant360_73Gene Nov2016 - Mar2019	Blood	10/30/2017	11/10/2017	5/13/2019	2
01-074	Ovarian serous adenocarcinoma	NF1_T1456P	Guardant360	Guardant360_73Gene Nov2016 - Mar2019	Blood	10/30/2017	11/10/2017	5/13/2019	
01-077	Renal (kidney clear cell adenocarcinoma, nos)	BRCA1_V802fs*7	STGA-DNA 2018	STGA-DNA 2018_11012021	Lymph Node	12/3/2015	10/5/2018	8/22/2019	4
01-077	Renal (kidney clear cell adenocarcinoma, nos)	BAP1_F81L	STGA-DNA 2018	STGA-DNA 2018_11012021	Lymph Node	12/3/2015	10/5/2018	8/22/2019	
01-079	salivary gland, nos	BRCA1_M1I	MI Profile	592 Gene Panel, Nov 2015-Jun 2021	Upper lobe, lung	3/8/2018	7/17/2019	11/13/2019	1
01-079	salivary gland, nos	TP53_R248W	MI Profile	592 Gene Panel, Nov 2015-Jun 2021	Upper lobe, lung	3/8/2018	7/17/2019	11/13/2019	
01-082	Breast (breast carcinoma, nos)	TP53_R248Q	STGA-DNA 2018	STGA-DNA 2018_11012021	Tumor biopsy-Liver	1/2/2019	2/19/2019	2/3/2020	
01-082	Breast (breast carcinoma, nos)	PTEN_E91fs*10	STGA-DNA 2018	STGA-DNA 2018_11012021	Tumor biopsy-Liver	1/2/2019	2/19/2019	2/3/2020	
01-082	Breast (breast carcinoma, nos)	PIK3CA_Q546P	STGA-DNA 2018	STGA-DNA 2018_11012021	Tumor biopsy-Liver	1/2/2019	2/19/2019	2/3/2020	
01-082	Breast (breast carcinoma, nos)	BRCA1_Q380*	STGA-DNA 2018	STGA-DNA 2018_11012021	Tumor biopsy-Liver	1/2/2019	2/19/2019	2/3/2020	3
01-086	Cholangiocarcinoma	BRAF_R389C	FoundationOne CDx	FoundationOne CDx_324/36 Dec 2017- Present	Liver	4/3/2018	7/24/2018	7/6/2020	
01-086	Cholangiocarcinoma	GNAS_R201C	FoundationOne CDx	FoundationOne CDx_324/36 Dec 2017- Present	Liver	4/3/2018	7/24/2018	7/6/2020	
01-086	Cholangiocarcinoma	HRAS_P167L	FoundationOne CDx	FoundationOne CDx_324/36 Dec 2017- Present	Liver	4/3/2018	7/24/2018	7/6/2020	
01-086	Cholangiocarcinoma	JAK3_D609Y	FoundationOne CDx	FoundationOne CDx_324/36 Dec 2017- Present	Liver	4/3/2018	7/24/2018	7/6/2020	
01-086	Cholangiocarcinoma	KDR_G438D	FoundationOne CDx	FoundationOne CDx_324/36 Dec 2017- Present	Liver	4/3/2018	7/24/2018	7/6/2020	
01-086	Cholangiocarcinoma	PDGFRA_T428N	FoundationOne CDx	FoundationOne CDx_324/36 Dec 2017- Present	Liver	4/3/2018	7/24/2018	7/6/2020	
01-086	Cholangiocarcinoma	PIK3CA_H1047R	FoundationOne CDx	FoundationOne CDx_324/36 Dec 2017- Present	Liver	4/3/2018	7/24/2018	7/6/2020	
01-086	Cholangiocarcinoma	TP53_R181H	FoundationOne CDx	FoundationOne CDx_324/36 Dec 2017- Present	Liver	4/3/2018	7/24/2018	7/6/2020	
01-086	Cholangiocarcinoma	TP53_R282W	FoundationOne CDx	FoundationOne CDx_324/36 Dec 2017- Present	Liver	4/3/2018	7/24/2018	7/6/2020	
01-086	Cholangiocarcinoma	BRCA1_E1001K	FoundationOne CDx	FoundationOne CDx_324/36 Dec 2017- Present	Liver	4/3/2018	7/24/2018	7/6/2020	3
01-086	Cholangiocarcinoma	BRCA2_R174H	FoundationOne CDx	FoundationOne CDx_324/36 Dec 2017- Present	Liver	4/3/2018	7/24/2018	7/6/2020	
01-086	Cholangiocarcinoma	ARID1A_R1335*	FoundationOne CDx	FoundationOne CDx_324/36 Dec 2017- Present	Liver	4/3/2018	7/24/2018	7/6/2020	
01-086	Cholangiocarcinoma	ARID1A_N1986D	FoundationOne CDx	FoundationOne CDx_324/36 Dec 2017- Present	Liver	4/3/2018	7/24/2018	7/6/2020	
01-086	Cholangiocarcinoma	CREBBP_T1990fs*9	FoundationOne CDx	FoundationOne CDx_324/36 Dec 2017- Present	Liver	4/3/2018	7/24/2018	7/6/2020	
01-086	Cholangiocarcinoma	EP300_N365S	FoundationOne CDx	FoundationOne CDx_324/36 Dec 2017- Present	Liver	4/3/2018	7/24/2018	7/6/2020	
01-086	Cholangiocarcinoma	FAM123B_P1088S	FoundationOne CDx	FoundationOne CDx_324/36 Dec 2017- Present	Liver	4/3/2018	7/24/2018	7/6/2020	
01-086	Cholangiocarcinoma	MLL2_N2517fs*26	FoundationOne CDx	FoundationOne CDx_324/36 Dec 2017- Present	Liver	4/3/2018	7/24/2018	7/6/2020	
01-086	Cholangiocarcinoma	PBRM1_R1160*	FoundationOne CDx	FoundationOne CDx_324/36 Dec 2017- Present	Liver	4/3/2018	7/24/2018	7/6/2020	
01-086	Cholangiocarcinoma	PTCH1_V1035L	FoundationOne CDx	FoundationOne CDx_324/36 Dec 2017- Present	Liver	4/3/2018	7/24/2018	7/6/2020	
01-086	Cholangiocarcinoma	WHSC1_Q13K	FoundationOne CDx	FoundationOne CDx_324/36 Dec 2017- Present	Liver	4/3/2018	7/24/2018	7/6/2020	
01-086	Cholangiocarcinoma	BCOR_P1587fs*53	FoundationOne CDx	FoundationOne CDx_324/36 Dec 2017- Present	Liver	4/3/2018	7/24/2018	7/6/2020	
01-086	Cholangiocarcinoma	EPHB1_R608Q	FoundationOne CDx	FoundationOne CDx_324/36 Dec 2017- Present	Liver	4/3/2018	7/24/2018	7/6/2020	
01-086	Cholangiocarcinoma	FANCL_P179S	FoundationOne CDx	FoundationOne CDx_324/36 Dec 2017- Present	Liver	4/3/2018	7/24/2018	7/6/2020	
01-086	Cholangiocarcinoma	FGF3_Y111C	FoundationOne CDx	FoundationOne CDx_324/36 Dec 2017- Present	Liver	4/3/2018	7/24/2018	7/6/2020	
01-086	Cholangiocarcinoma	KLHL6_V472A	FoundationOne CDx	FoundationOne CDx_324/36 Dec 2017- Present	Liver	4/3/2018	7/24/2018	7/6/2020	
01-086	Cholangiocarcinoma	SF3B1_K1267E	FoundationOne CDx	FoundationOne CDx_324/36 Dec 2017- Present	Liver	4/3/2018	7/24/2018	7/6/2020	
01-086	Cholangiocarcinoma	ZNF217_S769P	FoundationOne CDx	FoundationOne CDx_324/36 Dec 2017- Present	Liver	4/3/2018	7/24/2018	7/6/2020	
01-086	Cholangiocarcinoma	ZNF703_A586V	FoundationOne CDx	FoundationOne CDx_324/36 Dec 2017- Present	Liver	4/3/2018	7/24/2018	7/6/2020	
01-086	Cholangiocarcinoma	PPARG_T270P	FoundationOne CDx	FoundationOne CDx_324/36 Dec 2017- Present	Liver	4/3/2018	7/24/2018	7/6/2020	
01-086	Cholangiocarcinoma	POLD1_R409G	FoundationOne CDx	FoundationOne CDx_324/36 Dec 2017- Present	Liver	4/3/2018	7/24/2018	7/6/2020	

01-086	Cholangiocarcinoma	QKI_V52A	FoundationOne CDx	FoundationOne CDx_324/36 Dec 2017- Present	Liver	4/3/2018	7/24/2018	7/6/2020	
01-086	Cholangiocarcinoma	RBM10_V333D	FoundationOne CDx	FoundationOne CDx_324/36 Dec 2017- Present	Liver	4/3/2018	7/24/2018	7/6/2020	
01-086	Cholangiocarcinoma	PPP2R2A_P236Q	FoundationOne CDx	FoundationOne CDx_324/36 Dec 2017- Present	Liver	4/3/2018	7/24/2018	7/6/2020	
01-088	Breast (breast lobular and other ductal ca.)	NOTCH1_Amplification	STGA-DNA 2018	STGA-DNA 2018_11012021	Tumor biopsy- breast	3/13/2020	10/30/2020	10/21/2020	
01-088	Breast (breast lobular and other ductal ca.)	MYC_Amplification	STGA-DNA 2018	STGA-DNA 2018_11012021	Tumor biopsy- breast	3/13/2020	10/30/2020	10/21/2020	
01-088	Breast (breast lobular and other ductal ca.)	HIST1H3B_Amplification	STGA-DNA 2018	STGA-DNA 2018_11012021	Tumor biopsy- breast	3/13/2020	10/30/2020	10/21/2020	
01-088	Breast (breast lobular and other ductal ca.)	BRCA1_G1738_D1739delinsEN	STGA-DNA 2018	STGA-DNA 2018_11012021	Tumor biopsy- breast	3/13/2020	10/30/2020	10/21/2020	2
01-088	Breast (breast lobular and other ductal ca.)	TP53_R196*	STGA-DNA 2018	STGA-DNA 2018_11012021	Tumor biopsy- breast	3/13/2020	10/30/2020	10/21/2020	
01-090	Thyroid (thyroid gland carcinoma, nos)	MET_N375S	CMS50	CMS50_01042017	Lymph node	12/18/2014	9/18/2015	11/10/2020	
01-090	Thyroid (thyroid gland carcinoma, nos)	PTEN_R335*	CMS50	CMS50_01042017	Lymph node	12/18/2014	9/18/2015	11/10/2020	
01-090	Thyroid (thyroid gland carcinoma, nos)	RB1_L676fs*15	CMS50	CMS50_01042017	Lymph node	12/18/2014	9/18/2015	11/10/2020	
01-090	Thyroid (thyroid gland carcinoma, nos)	RB1_L676fs	Guardant360	Guardant360_74Gene_Mar2019-Sept2020	Blood	4/8/2019	4/16/2019	11/10/2020	
01-090	Thyroid (thyroid gland carcinoma, nos)	STK11_Splice	Guardant360	Guardant360_74Gene_Mar2019-Sept2020	Blood	4/8/2019	4/16/2019	11/10/2020	
01-090	Thyroid (thyroid gland carcinoma, nos)	BRCA1_Q657*	Guardant360	Guardant360_73Gene Nov2016 - Mar2019	Blood	1/17/2019	1/26/2019	11/10/2020	2
01-090	Thyroid (thyroid gland carcinoma, nos)	NF1_W1997fs	Guardant360	Guardant360_73Gene Nov2016 - Mar2019	Blood	1/17/2019	1/26/2019	11/10/2020	
01-091	Lung (lung & bronchus squamous cell carcinoma, r	TP53_R273C	STGA-DNA 2018	STGA-DNA 2018_11012021	Biopsy- bronchus, endobronchial	1/15/2020	2/7/2020	5/26/2021	
01-091	Lung (lung & bronchus squamous cell carcinoma, r	CDK12_G1441A	STGA-DNA 2018	STGA-DNA 2018_11012021	Biopsy- bronchus, endobronchial	1/15/2020	2/7/2020	5/26/2021	
01-091	Lung (lung & bronchus squamous cell carcinoma, r	PIK3CA_E545K	STGA-DNA 2018	STGA-DNA 2018_11012021	Biopsy- bronchus, endobronchial	1/15/2020	2/7/2020	5/26/2021	
01-091	Lung (lung & bronchus squamous cell carcinoma, r	BRCA1_K672*	STGA-DNA 2018	STGA-DNA 2018_11012021	Biopsy- bronchus, endobronchial	1/15/2020	2/7/2020	5/26/2021	2
01-091	Lung (lung & bronchus squamous cell carcinoma, r	MDM2_Amplification	STGA-DNA 2018	STGA-DNA 2018_11012021	Biopsy- bronchus, endobronchial	1/15/2020	2/7/2020	5/26/2021	
01-092	Colon adenocarcinoma (crc)	RB1_Amplification	STGAv1	STGAv1_03132018	Biopsy	4/6/2017	5/22/2017	5/27/2021	
01-092	Colon adenocarcinoma (crc)	APC_T1301fs*14	STGAv1	STGAv1_03132018	Biopsy	4/6/2017	5/22/2017	5/27/2021	
01-092	Colon adenocarcinoma (crc)	BRCA2_S2186fs*3	STGAv1	STGAv1_03132018	Biopsy	4/6/2017	5/22/2017	5/27/2021	7
01-092	Colon adenocarcinoma (crc)	PIK3R1_V589fs*13	STGAv1	STGAv1_03132018	Biopsy	4/6/2017	5/22/2017	5/27/2021	
01-092	Colon adenocarcinoma (crc)	TP53_R273H	STGAv1	STGAv1_03132018	Biopsy	4/6/2017	5/22/2017	5/27/2021	
01-092	Colon adenocarcinoma (crc)	APC_R1171fs*8	STGAv1	STGAv1_03132018	Biopsy	4/6/2017	5/22/2017	5/27/2021	
01-092	Colon adenocarcinoma (crc)	SLX4_P1378L	STGA-DNA 2018	STGA-DNA 2018_11012021	Tumor resection- low anterior	10/30/2013	4/19/2021	5/27/2021	
01-092	Colon adenocarcinoma (crc)	TP53_R273H	STGA-DNA 2018	STGA-DNA 2018_11012021	Tumor resection- low anterior	10/30/2013	4/19/2021	5/27/2021	
01-093	Anorectal melanoma	CCND1_Amplification	STGA-DNA 2018	STGA-DNA 2018_11012021	Biopsy-anus	10/27/2020	3/28/2021	7/29/2021	
01-093	Anorectal melanoma	BRCA2_S2186fs*3	STGA-DNA 2018	STGA-DNA 2018_11012021	Biopsy-anus	10/27/2020	3/28/2021	7/29/2021	1
02-002	Rectum adenocarcinoma (crc)	KRAS_G12D	CMS50	CMS50_01042017	Biopsy-rectal	3/4/2013	1/22/2015	1/9/2015	
02-002	Rectum adenocarcinoma (crc)	SMAD4_R361C	CMS50	CMS50_01042017	Biopsy-rectal	3/4/2013	1/22/2015	1/9/2015	
02-002	Rectum adenocarcinoma (crc)	KRAS_G12D	FoundationOne	FoundationOne_236/19 Dec 2012 - Aug 2014	Liver	6/14/2013	6/12/2014	1/9/2015	
02-002	Rectum adenocarcinoma (crc)	ATM_I389fs*1	FoundationOne	FoundationOne_236/19 Dec 2012 - Aug 2014	Liver	6/14/2013	6/12/2014	1/9/2015	4
02-002	Rectum adenocarcinoma (crc)	SMAD4_R361C	FoundationOne	FoundationOne_236/19 Dec 2012 - Aug 2014	Liver	6/14/2013	6/12/2014	1/9/2015	
02-002	Rectum adenocarcinoma (crc)	ASXL1_P202L	FoundationOne	FoundationOne_236/19 Dec 2012 - Aug 2014	Liver	6/14/2013	6/12/2014	1/9/2015	
02-002	Rectum adenocarcinoma (crc)	LRP1B_L4021R	FoundationOne	FoundationOne_236/19 Dec 2012 - Aug 2014	Liver	6/14/2013	6/12/2014	1/9/2015	
02-002	Rectum adenocarcinoma (crc)	LRP1B_V3395I	FoundationOne	FoundationOne_236/19 Dec 2012 - Aug 2014	Liver	6/14/2013	6/12/2014	1/9/2015	
02-002	Rectum adenocarcinoma (crc)	MAP2K4_A378S	FoundationOne	FoundationOne_236/19 Dec 2012 - Aug 2014	Liver	6/14/2013	6/12/2014	1/9/2015	
02-002	Rectum adenocarcinoma (crc)	MLL_A67del	FoundationOne	FoundationOne_236/19 Dec 2012 - Aug 2014	Liver	6/14/2013	6/12/2014	1/9/2015	
02-002	Rectum adenocarcinoma (crc)	MYST3_Q1677_1680del	FoundationOne	FoundationOne_236/19 Dec 2012 - Aug 2014	Liver	6/14/2013	6/12/2014	1/9/2015	
02-002	Rectum adenocarcinoma (crc)	PTCH1_I491M	FoundationOne	FoundationOne_236/19 Dec 2012 - Aug 2014	Liver	6/14/2013	6/12/2014	1/9/2015	
02-002	Rectum adenocarcinoma (crc)	RNF43_V107G	FoundationOne	FoundationOne_236/19 Dec 2012 - Aug 2014	Liver	6/14/2013	6/12/2014	1/9/2015	
02-002	Rectum adenocarcinoma (crc)	FGF14_Amplification	FoundationOne	FoundationOne_236/19 Dec 2012 - Aug 2014	Liver	6/14/2013	6/12/2014	1/9/2015	
02-002	Rectum adenocarcinoma (crc)	IRS2_Amplification	FoundationOne	FoundationOne_236/19 Dec 2012 - Aug 2014	Liver	6/14/2013	6/12/2014	1/9/2015	
02-003	Breast adenocarcinoma	KIT_M541L	CMS46	CMS46_07242015	Biopsy-left thigh nodule	12/30/2008	3/20/2013	1/22/2015	
02-003	Breast adenocarcinoma	PIK3CA_E545K	CMS46	CMS46_07242015	Biopsy-left thigh nodule	12/30/2008	3/20/2013	1/22/2015	
02-003	Breast adenocarcinoma	EMSY amplification	FoundationOne	FoundationOne_315/28 Aug 2014 - Dec 2017	Liver	9/10/2014	9/25/2014	1/22/2015	1
02-003	Breast adenocarcinoma	PIK3CA_E545K	FoundationOne	FoundationOne_315/28 Aug 2014 - Dec 2017	Liver	9/10/2014	9/25/2014	1/22/2015	
02-003	Breast adenocarcinoma	ARID1A_S138*	FoundationOne	FoundationOne_315/28 Aug 2014 - Dec 2017	Liver	9/10/2014	9/25/2014	1/22/2015	
02-003	Breast adenocarcinoma	ARID1B_P450_S451insP	FoundationOne	FoundationOne_315/28 Aug 2014 - Dec 2017	Liver	9/10/2014	9/25/2014	1/22/2015	
02-003	Breast adenocarcinoma	AURKB_S37C	FoundationOne	FoundationOne_315/28 Aug 2014 - Dec 2017	Liver	9/10/2014	9/25/2014	1/22/2015	
02-003	Breast adenocarcinoma	C17orf39_S100Y	FoundationOne	FoundationOne_315/28 Aug 2014 - Dec 2017	Liver	9/10/2014	9/25/2014	1/22/2015	
02-003	Breast adenocarcinoma	CIC_P853S	FoundationOne	FoundationOne_315/28 Aug 2014 - Dec 2017	Liver	9/10/2014	9/25/2014	1/22/2015	
02-003	Breast adenocarcinoma	FANCF_A186V	FoundationOne	FoundationOne_315/28 Aug 2014 - Dec 2017	Liver	9/10/2014	9/25/2014	1/22/2015	
02-003	Breast adenocarcinoma	FOXP1_N570S	FoundationOne	FoundationOne_315/28 Aug 2014 - Dec 2017	Liver	9/10/2014	9/25/2014	1/22/2015	
02-003	Breast adenocarcinoma	GPR124_T348I	FoundationOne	FoundationOne_315/28 Aug 2014 - Dec 2017	Liver	9/10/2014	9/25/2014	1/22/2015	
02-003	Breast adenocarcinoma	INPP4B_K782R	FoundationOne	FoundationOne_315/28 Aug 2014 - Dec 2017	Liver	9/10/2014	9/25/2014	1/22/2015	
02-003	Breast adenocarcinoma	MYC_S344I	FoundationOne	FoundationOne_315/28 Aug 2014 - Dec 2017	Liver	9/10/2014	9/25/2014	1/22/2015	
02-003	Breast adenocarcinoma	PDCD1LG2_*274S	FoundationOne	FoundationOne_315/28 Aug 2014 - Dec 2017	Liver	9/10/2014	9/25/2014	1/22/2015	
02-003	Breast adenocarcinoma	PDGFRB_E15K	FoundationOne	FoundationOne_315/28 Aug 2014 - Dec 2017	Liver	9/10/2014	9/25/2014	1/22/2015	
02-006	Angiosarcoma	ATM_R2832C	FoundationOne Heme	FoundationOne Heme_406/31/265	Skin	8/24/2012	4/16/2014	2/24/2015	7
02-006	Angiosarcoma	TP53_S127F	FoundationOne Heme	FoundationOne Heme_406/31/265	Skin	8/24/2012	4/16/2014	2/24/2015	
02-006	Angiosarcoma	FGFR2_W4*	FoundationOne Heme	FoundationOne Heme_406/31/265	Skin	8/24/2012	4/16/2014	2/24/2015	
02-006	Angiosarcoma	AURKA_A17G	FoundationOne Heme	FoundationOne Heme_406/31/265	Skin	8/24/2012	4/16/2014	2/24/2015	
02-006	Angiosarcoma	FAM46C_V69A	FoundationOne Heme	FoundationOne Heme_406/31/265	Skin	8/24/2012	4/16/2014	2/24/2015	
02-006	Angiosarcoma	FGFR2_E163K	FoundationOne Heme	FoundationOne Heme_406/31/265	Skin	8/24/2012	4/16/2014	2/24/2015	
02-006	Angiosarcoma	KDR_P351S	FoundationOne Heme	FoundationOne Heme_406/31/265	Skin	8/24/2012	4/16/2014	2/24/2015	
02-006	Angiosarcoma	KIT_E767Q	FoundationOne Heme	FoundationOne Heme_406/31/265	Skin	8/24/2012	4/16/2014	2/24/2015	
02-006	Angiosarcoma	LRRK2_E1544K	FoundationOne Heme	FoundationOne Heme_406/31/265	Skin	8/24/2012	4/16/2014	2/24/2015	
02-006	Angiosarcoma	MET_L496V	FoundationOne Heme	FoundationOne Heme_406/31/265	Skin	8/24/2012	4/16/2014	2/24/2015	
02-006	Angiosarcoma	MLH1_A424T	FoundationOne Heme	FoundationOne Heme_406/31/265	Skin	8/24/2012	4/16/2014	2/24/2015	
02-006	Angiosarcoma	MLL3_N2741I	FoundationOne Heme	FoundationOne Heme_406/31/265	Skin	8/24/2012	4/16/2014	2/24/2015	
02-006	Angiosarcoma	MYCL1_P175L	FoundationOne Heme	FoundationOne Heme_406/31/265	Skin	8/24/2012	4/16/2014	2/24/2015	
02-006	Angiosarcoma	PDCD11_Q627R	FoundationOne Heme	FoundationOne Heme_406/31/265	Skin	8/24/2012	4/16/2014	2/24/2015	
02-006	Angiosarcoma	PDGFRA_V607M	FoundationOne Heme	FoundationOne Heme_406/31/265	Skin	8/24/2012	4/16/2014	2/24/2015	
02-006	Angiosarcoma	POT1_D77N	FoundationOne Heme	FoundationOne Heme_406/31/265	Skin	8/24/2012	4/16/2014	2/24/2015	
02-006	Angiosarcoma	PRDM1_D68N	FoundationOne Heme	FoundationOne Heme_406/31/265	Skin	8/24/2012	4/16/2014	2/24/2015	
02-006	Angiosarcoma	RARA_T233A	FoundationOne Heme	FoundationOne Heme_406/31/265	Skin	8/24/2012	4/16/2014	2/24/2015	
02-006	Angiosarcoma	RELN_M2484K	FoundationOne Heme	FoundationOne Heme_406/31/265	Skin	8/24/2012	4/16/2014	2/24/2015	
02-006	Angiosarcoma	S1PR2_R315Q	FoundationOne Heme	FoundationOne Heme_406/31/265	Skin	8/24/2012	4/16/2014	2/24/2015	
02-006	Angiosarcoma	SPEN_G2022S	FoundationOne Heme	FoundationOne Heme_406/31/265	Skin	8/24/2012	4/16/2014	2/24/2015	
02-006	Angiosarcoma	SYK_K393T	FoundationOne Heme	FoundationOne Heme_406/31/265	Skin	8/24/2012	4/16/2014	2/24/2015	
02-006	Angiosarcoma	TRAF3_P516S	FoundationOne Heme	FoundationOne Heme_406/31/265	Skin	8/24/2012	4/16/2014	2/24/2015	
02-006	Angiosarcoma	TSHR_P48S	FoundationOne Heme	FoundationOne Heme_406/31/265	Skin	8/24/2012	4/16/2014	2/24/2015	
02-006	Angiosarcoma	BRAF_Amplification	FoundationOne Heme	FoundationOne Heme_406/31/265	Skin	8/24/2012	4/16/2014	2/24/2015	
02-006	Angiosarcoma	KDR_Amplification	FoundationOne Heme	FoundationOne Heme_406/31/265	Skin	8/24/2012	4/16/2014	2/24/2015	
02-006	Angiosarcoma	KIT_Amplification	FoundationOne Heme	FoundationOne Heme_406/31/265	Skin	8/24/2012	4/16/2014	2/24/2015	
02-006	Angiosarcoma	PDGFRA_Amplification	FoundationOne Heme	FoundationOne Heme_406/31/265	Skin	8/24/2012	4/16/2014	2/24/2015	
02-006	Angiosarcoma	SMO_Amplification	FoundationOne Heme	FoundationOne Heme_406/31/265	Skin	8/24/2012	4/16/2014	2/24/2015	
02-006	Angiosarcoma	EZH2_Amplification	FoundationOne Heme	FoundationOne Heme_406/31/265	Skin	8/24/2012	4/16/2014	2/24/2015	
02-006	Angiosarcoma	MYC_Amplification	FoundationOne Heme	FoundationOne Heme_406/31/265	Skin	8/24/2012	4/16/2014	2/24/2015	
02-006	Angiosarcoma	FLCN_Amplification	FoundationOne Heme	FoundationOne Heme_406/31/265	Skin	8/24/2012	4/16/2014	2/24/2015	
02-006	Angiosarcoma	POT1_Amplification	FoundationOne Heme	FoundationOne Heme_406/31/265	Skin	8/24/2012	4/16/2014	2/24/2015	
02-006	Angiosarcoma	KMT2C_Amplification	FoundationOne Heme	FoundationOne Heme_406/31/265	Skin	8/24/2012	4/16/2014	2/24/2015	
02-006	Angiosarcoma	RAD21_Amplification	FoundationOne Heme	FoundationOne Heme_406/31/265	Skin	8/24/2012	4/16/2014	2/24/2015	
02-006	Angiosarcoma	C17orf39_Amplification	FoundationOne Heme	FoundationOne Heme_406/31/265	Skin	8/24/2012	4/16/2014	2/24/2015	
02-007	Ovarian high grade serous carcinoma	SMARCB1_Amplification	FoundationOne	FoundationOne_236/19 Dec 2012 - Aug 2014	Lymph Node	8/23/2013	8/4/2014	3/5/2015	
02-007	Ovarian high grade serous carcinoma	NF2_Amplification	FoundationOne	FoundationOne_236/19 Dec 2012 - Aug 2014	Lymph Node	8/23/2013	8/4/2014	3/5/2015	
02-007	Ovarian high grade serous carcinoma	ARFRP1_Amplification	FoundationOne	FoundationOne_236/19 Dec 2012 - Aug 2014	Lymph Node	8/23/2013	8/4/2014	3/5/2015	
02-007	Ovarian high grade serous carcinoma	CRKL_Amplification	FoundationOne	FoundationOne_236/19 Dec 2012 - Aug 2014	Lymph Node	8/23/2013	8/4/2014	3/5/2015	
02-007	Ovarian high grade serous carcinoma	PIK3CA_I391M	CMS50	CMS50_01042017	Colon mensentary	8/12/2009	7/7/2014	3/5/2015	
02-007	Ovarian high grade serous carcinoma	TP53_P191fs*56	CMS50	CMS50_01042017	Colon mensentary	8/12/2009	7/7/2014	3/5/2015	
02-007	Ovarian high grade serous carcinoma	TP53_P190fs*57	FoundationOne	FoundationOne_236/19 Dec 2012 - Aug 2014	Lymph Node	8/23/2013	8/4/2014	3/5/2015	
02-007	Ovarian high grade serous carcinoma	BRIP1_A402fs*21	FoundationOne	FoundationOne_236/19 Dec 2012 - Aug 2014	Lymph Node	8/23/2013	8/4/2014	3/5/2015	3
02-007	Ovarian high grade serous carcinoma	BCORL1_R1297Q	FoundationOne	FoundationOne_236/19 Dec 2012 - Aug 2014	Lymph Node	8/23/2013	8/4/2014	3/5/2015	
02-007	Ovarian high grade serous carcinoma	CARD11_N501K	FoundationOne	FoundationOne_236/19 Dec 2012 - Aug 2014	Lymph Node	8/23/2013	8/4/2014	3/5/2015	
02-007	Ovarian high grade serous carcinoma	FGF14_N43S	FoundationOne	FoundationOne_236/19 Dec 2012 - Aug 2014	Lymph Node	8/23/2013	8/4/2014	3/5/2015	
02-007	Ovarian high grade serous carcinoma	GPR124_R593_L605del	FoundationOne	FoundationOne_236/19 Dec 2012 - Aug 2014	Lymph Node	8/23/2013	8/4/2014	3/5/2015	
02-007	Ovarian high grade serous carcinoma	IRS2_P1015L	FoundationOne	FoundationOne_236/19 Dec 2012 - Aug 2014	Lymph Node	8/23/2013	8/4/2014	3/5/2015	
02-007	Ovarian high grade serous carcinoma	LRP1B_R2219C	FoundationOne	FoundationOne_236/19 Dec 2012 - Aug 2014	Lymph Node	8/23/2013	8/4/2014	3/5/2015	
02-007	Ovarian high grade serous carcinoma	NTRK1_G18E	FoundationOne	FoundationOne_236/19 Dec 2012 - Aug 2014	Lymph Node	8/23/2013	8/4/2014	3/5/2015	
02-007	Ovarian high grade serous carcinoma	PDGFRA_V38A	FoundationOne	FoundationOne_236/19 Dec 2012 - Aug 2014	Lymph Node	8/23/2013	8/4/2014	3/5/2015	
02-007	Ovarian high grade serous carcinoma	RICTOR_S52*	FoundationOne	FoundationOne_236/19 Dec 2012 - Aug 2014	Lymph Node	8/23/2013	8/4/2014	3/5/2015	
02-007	Ovarian high grade serous carcinoma	RICTOR_S35C	FoundationOne	FoundationOne_236/19 Dec 2012 - Aug 2014	Lymph Node	8/23/2013	8/4/2014	3/5/2015	
02-007	Ovarian high grade serous carcinoma	BCR-ZNRF3	FoundationOne	FoundationOne_236/19 Dec 2012 - Aug 2014	Lymph Node	8/23/2013	8/4/2014	3/5/2015	
02-007	Ovarian high grade serous carcinoma	NTRK3	FoundationOne	FoundationOne_236/19 Dec 2012 - Aug 2014	Lymph Node	8/23/2013	8/4/2014	3/5/2015	
02-011	Urothelial carcinoma	ATM_G844E	CMS50	CMS50_01042017	Lymph Node	11/7/2011	3/4/2014	3/25/2015	
02-011	Urothelial carcinoma	TERT_promoter -124C>T	FoundationOne	FoundationOne_315/28 Aug 2014 - Dec 2017	Bladder	6/16/2011	10/18/2014	3/25/2015	
02-011	Urothelial carcinoma	ATR D1104fs*6	FoundationOne	FoundationOne 315/28 Aug 2014 - Dec 2017	Bladder	6/16/2011	10/18/2014	3/25/2015	4
02-011	Urothelial carcinoma	FAT1_Q3524*	FoundationOne	FoundationOne_315/28 Aug 2014 - Dec 2017	Bladder	6/16/2011	10/18/2014	3/25/2015	
02-011	Urothelial carcinoma	TSC1_c.107-1G>C	FoundationOne	FoundationOne_315/28 Aug 2014 - Dec 2017	Bladder	6/16/2011	10/18/2014	3/25/2015	
02-011	Urothelial carcinoma	ATM_G844E	FoundationOne	FoundationOne_315/28 Aug 2014 - Dec 2017	Bladder	6/16/2011	10/18/2014	3/25/2015	
02-011	Urothelial carcinoma	CSF1R_G591R	FoundationOne	FoundationOne_315/28 Aug 2014 - Dec 2017	Bladder	6/16/2011	10/18/2014	3/25/2015	
02-011	Urothelial carcinoma	FAM123B_H134P	FoundationOne	FoundationOne_315/28 Aug 2014 - Dec 2017	Bladder	6/16/2011	10/18/2014	3/25/2015	
02-011	Urothelial carcinoma	FAT1_E3490Q	FoundationOne	FoundationOne_315/28 Aug 2014 - Dec 2017	Bladder	6/16/2011	10/18/2014	3/25/2015	
02-011	Urothelial carcinoma	GATA2_P161A	FoundationOne	FoundationOne_315/28 Aug 2014 - Dec 2017	Bladder	6/16/2011	10/18/2014	3/25/2015	
02-011	Urothelial carcinoma	GSK3B_E283K	FoundationOne	FoundationOne_315/28 Aug 2014 - Dec 2017	Bladder	6/16/2011	10/18/2014	3/25/2015	
02-011	Urothelial carcinoma	JAK2_Q771*	FoundationOne	FoundationOne_315/28 Aug 2014 - Dec 2017	Bladder	6/16/2011	10/18/2014	3/25/2015	
02-011	Urothelial carcinoma	MED12_Q2120_Q2121>HQQQQQ	FoundationOne	FoundationOne_315/28 Aug 2014 - Dec 2017	Bladder	6/16/2011	10/18/2014	3/25/2015	
02-011	Urothelial carcinoma	MYST3_E1393_E1397del	FoundationOne	FoundationOne_315/28 Aug 2014 - Dec 2017	Bladder	6/16/2011	10/18/2014	3/25/2015	
02-011	Urothelial carcinoma	PPP2R1A_H87Y	FoundationOne	FoundationOne_315/28 Aug 2014 - Dec 2017	Bladder	6/16/2011	10/18/2014	3/25/2015	
02-011	Urothelial carcinoma	TET2_S1820T	FoundationOne	FoundationOne_315/28 Aug 2014 - Dec 2017	Bladder	6/16/2011	10/18/2014	3/25/2015	
02-011	Urothelial carcinoma	PDGFRA-PDGFRA	FoundationOne	FoundationOne_315/28 Aug 2014 - Dec 2017	Bladder	6/16/2011	10/18/2014	3/25/2015	
02-011	Urothelial carcinoma	CDKN2A_Deletion	FoundationOne	FoundationOne_315/28 Aug 2014 - Dec 2017	Bladder	6/16/2011	10/18/2014	3/25/2015	
02-011	Urothelial carcinoma	CDKN2B_Deletion	FoundationOne	FoundationOne_315/28 Aug 2014 - Dec 2017	Bladder	6/16/2011	10/18/2014	3/25/2015	
02-011	Urothelial carcinoma	MDM2_Amplification	FoundationOne	FoundationOne_315/28 Aug 2014 - Dec 2017	Bladder	6/16/2011	10/18/2014	3/25/2015	
02-011	Urothelial carcinoma	FRS2_Amplification	FoundationOne	FoundationOne_315/28 Aug 2014 - Dec 2017	Bladder	6/16/2011	10/18/2014	3/25/2015	
02-012	Ampullary adenocarinoma	KIT_M541L	CMS50	CMS50_01042017	Tumor biopsy- ampulla of vater	10/10/2013	8/21/2014	4/15/2015	
02-012	Ampullary adenocarinoma	PIK3R1_R409Q	FoundationOne	FoundationOne_315/28 Aug 2014 - Dec 2017	Liver	8/25/2014	12/2/2014	4/15/2015	
02-012	Ampullary adenocarinoma	ATM_K2643fs*12	FoundationOne	FoundationOne_315/28 Aug 2014 - Dec 2017	Liver	8/25/2014	12/2/2014	4/15/2015	1
02-012	Ampullary adenocarinoma	APC_R2505Q	FoundationOne	FoundationOne_315/28 Aug 2014 - Dec 2017	Liver	8/25/2014	12/2/2014	4/15/2015	
02-012	Ampullary adenocarinoma	DNMT3A_D531N	FoundationOne	FoundationOne_315/28 Aug 2014 - Dec 2017	Liver	8/25/2014	12/2/2014	4/15/2015	
02-012	Ampullary adenocarinoma	GRIN2A_L887V	FoundationOne	FoundationOne_315/28 Aug 2014 - Dec 2017	Liver	8/25/2014	12/2/2014	4/15/2015	
02-012	Ampullary adenocarinoma	NFKBIA_R143Q	FoundationOne	FoundationOne_315/28 Aug 2014 - Dec 2017	Liver	8/25/2014	12/2/2014	4/15/2015	
02-012	Ampullary adenocarinoma	PBRM1_F594S	FoundationOne	FoundationOne_315/28 Aug 2014 - Dec 2017	Liver	8/25/2014	12/2/2014	4/15/2015	
02-012	Ampullary adenocarinoma	PBRM1_E133K	FoundationOne	FoundationOne_315/28 Aug 2014 - Dec 2017	Liver	8/25/2014	12/2/2014	4/15/2015	
02-012	Ampullary adenocarinoma	PDGFRA_R764H	FoundationOne	FoundationOne_315/28 Aug 2014 - Dec 2017	Liver	8/25/2014	12/2/2014	4/15/2015	
02-012	Ampullary adenocarinoma	PREX2_R1394Q	FoundationOne	FoundationOne_315/28 Aug 2014 - Dec 2017	Liver	8/25/2014	12/2/2014	4/15/2015	

02-012	Ampullary adenocarinoma	CDKN2A_Deletion	FoundationOne	FoundationOne_315/28 Aug 2014 - Dec 2017	Liver	8/25/2014	12/2/2014	4/15/2015	
02-012	Ampullary adenocarinoma	SMAD4_Deletion	FoundationOne	FoundationOne_315/28 Aug 2014 - Dec 2017	Liver	8/25/2014	12/2/2014	4/15/2015	
02-012	Ampullary adenocarinoma	ARID1A_Deletion	FoundationOne	FoundationOne_315/28 Aug 2014 - Dec 2017	Liver	8/25/2014	12/2/2014	4/15/2015	
02-012	Ampullary adenocarinoma	CDKN2B_Deletion	FoundationOne	FoundationOne_315/28 Aug 2014 - Dec 2017	Liver	8/25/2014	12/2/2014	4/15/2015	
02-012	Ampullary adenocarinoma	MYCL1_Amplification	FoundationOne	FoundationOne_315/28 Aug 2014 - Dec 2017	Liver	8/25/2014	12/2/2014	4/15/2015	
02-014	Breast adenocarcinoma	PIK3CA_E545K	FoundationOne	FoundationOne_315/28 Aug 2014 - Dec 2017	Breast	8/20/2012	2/18/2015	4/13/2015	
02-014	Breast adenocarcinoma	PTEN_R233*	FoundationOne	FoundationOne_315/28 Aug 2014 - Dec 2017	Breast	8/20/2012	2/18/2015	4/13/2015	
02-014	Breast adenocarcinoma	RB1_Y655*	FoundationOne	FoundationOne_315/28 Aug 2014 - Dec 2017	Breast	8/20/2012	2/18/2015	4/13/2015	
02-014	Breast adenocarcinoma	TP53_R273C	FoundationOne	FoundationOne_315/28 Aug 2014 - Dec 2017	Breast	8/20/2012	2/18/2015	4/13/2015	
02-014	Breast adenocarcinoma	FANCC_S119fs*8	FoundationOne	FoundationOne_315/28 Aug 2014 - Dec 2017	Breast	8/20/2012	2/18/2015	4/13/2015	3
02-014	Breast adenocarcinoma	FANCC_H120Q	FoundationOne	FoundationOne_315/28 Aug 2014 - Dec 2017	Breast	8/20/2012	2/18/2015	4/13/2015	
02-014	Breast adenocarcinoma	ERRFI1_R400*	FoundationOne	FoundationOne_315/28 Aug 2014 - Dec 2017	Breast	8/20/2012	2/18/2015	4/13/2015	
02-014	Breast adenocarcinoma	AKT1_Amplification	FoundationOne	FoundationOne_315/28 Aug 2014 - Dec 2017	Breast	8/20/2012	2/18/2015	4/13/2015	
02-014	Breast adenocarcinoma	PIK3CA_Amplification	FoundationOne	FoundationOne_315/28 Aug 2014 - Dec 2017	Breast	8/20/2012	2/18/2015	4/13/2015	
02-014	Breast adenocarcinoma	ATR_Amplification	FoundationOne	FoundationOne_315/28 Aug 2014 - Dec 2017	Breast	8/20/2012	2/18/2015	4/13/2015	
02-014	Breast adenocarcinoma	PIK3CB_Amplification	FoundationOne	FoundationOne_315/28 Aug 2014 - Dec 2017	Breast	8/20/2012	2/18/2015	4/13/2015	
02-023	Angiosarcoma of breast	RAD51_Q243*	FoundationOne Heme	FoundationOne Heme_406/31/265	skin	9/25/2014	5/5/2015	7/21/2015	2
02-023	Angiosarcoma of breast	ALK_E310D	FoundationOne Heme	FoundationOne Heme_406/31/265	skin	9/25/2014	5/5/2015	7/21/2015	
02-023	Angiosarcoma of breast	APC_R2505Q	FoundationOne Heme	FoundationOne Heme_406/31/265	skin	9/25/2014	5/5/2015	7/21/2015	
02-023	Angiosarcoma of breast	CIC_A536G	FoundationOne Heme	FoundationOne Heme_406/31/265	skin	9/25/2014	5/5/2015	7/21/2015	
02-023	Angiosarcoma of breast	DOT1L_G1452_A1458del	FoundationOne Heme	FoundationOne Heme_406/31/265	skin	9/25/2014	5/5/2015	7/21/2015	
02-023	Angiosarcoma of breast	FAM123B_A812T	FoundationOne Heme	FoundationOne Heme_406/31/265	skin	9/25/2014	5/5/2015	7/21/2015	
02-023	Angiosarcoma of breast	FGF14_R30Q	FoundationOne Heme	FoundationOne Heme_406/31/265	skin	9/25/2014	5/5/2015	7/21/2015	
02-023	Angiosarcoma of breast	IRS2_L20_N21insN	FoundationOne Heme	FoundationOne Heme_406/31/265	skin	9/25/2014	5/5/2015	7/21/2015	
02-023	Angiosarcoma of breast	KDR_N704del	FoundationOne Heme	FoundationOne Heme_406/31/265	skin	9/25/2014	5/5/2015	7/21/2015	
02-023	Angiosarcoma of breast	PIK3CA_F83I	FoundationOne Heme	FoundationOne Heme_406/31/265	skin	9/25/2014	5/5/2015	7/21/2015	
02-023	Angiosarcoma of breast	SMARCA4_P1277L	FoundationOne Heme	FoundationOne Heme_406/31/265	skin	9/25/2014	5/5/2015	7/21/2015	
02-023	Angiosarcoma of breast	TGFBR2_D379V	FoundationOne Heme	FoundationOne Heme_406/31/265	skin	9/25/2014	5/5/2015	7/21/2015	
02-032	Cholangiocarcinoma	ATM_G1676fs	ClearPoint	Other	Biopsy-liver	9/21/2015	10/1/2015	10/27/2015	
02-033	Myxoid sarcoma	ARID2_T491S	FoundationOne Heme	FoundationOne Heme_406/31/265	Peritoneum	4/18/2014	8/28/2015	11/3/2015	
02-033	Myxoid sarcoma	AXL_Y367*	FoundationOne Heme	FoundationOne Heme_406/31/265	Peritoneum	4/18/2014	8/28/2015	11/3/2015	
02-033	Myxoid sarcoma	BCOR_G559E	FoundationOne Heme	FoundationOne Heme_406/31/265	Peritoneum	4/18/2014	8/28/2015	11/3/2015	
02-033	Myxoid sarcoma	BRD4_P1019R	FoundationOne Heme	FoundationOne Heme_406/31/265	Peritoneum	4/18/2014	8/28/2015	11/3/2015	
02-033	Myxoid sarcoma	FAM46C_G243fs*40	FoundationOne Heme	FoundationOne Heme_406/31/265	Peritoneum	4/18/2014	8/28/2015	11/3/2015	
02-033	Myxoid sarcoma	FGFR1_D69N	FoundationOne Heme	FoundationOne Heme_406/31/265	Peritoneum	4/18/2014	8/28/2015	11/3/2015	
02-033	Myxoid sarcoma	FLCN_A45G	FoundationOne Heme	FoundationOne Heme_406/31/265	Peritoneum	4/18/2014	8/28/2015	11/3/2015	
02-033	Myxoid sarcoma	MYO18A_R1302C	FoundationOne Heme	FoundationOne Heme_406/31/265	Peritoneum	4/18/2014	8/28/2015	11/3/2015	
02-033	Myxoid sarcoma	NCOR2_P154L	FoundationOne Heme	FoundationOne Heme_406/31/265	Peritoneum	4/18/2014	8/28/2015	11/3/2015	
02-033	Myxoid sarcoma	SETBP1_T1547N	FoundationOne Heme	FoundationOne Heme_406/31/265	Peritoneum	4/18/2014	8/28/2015	11/3/2015	
02-033	Myxoid sarcoma	TBL1XR1-USP22	FoundationOne Heme	FoundationOne Heme_406/31/265	Peritoneum	4/18/2014	8/28/2015	11/3/2015	
02-033	Myxoid sarcoma	FANCC_deletion_exons_2-8	FoundationOne Heme	FoundationOne Heme_406/31/265	Peritoneum	4/18/2014	8/28/2015	11/3/2015	3
02-033	Myxoid sarcoma	PTCH1_Deletion	FoundationOne Heme	FoundationOne Heme_406/31/265	Peritoneum	4/18/2014	8/28/2015	11/3/2015	
02-033	Myxoid sarcoma	SDHA_Amplification	FoundationOne Heme	FoundationOne Heme_406/31/265	Peritoneum	4/18/2014	8/28/2015	11/3/2015	
02-033	Myxoid sarcoma	SGK1_Amplification	FoundationOne Heme	FoundationOne Heme_406/31/265	Peritoneum	4/18/2014	8/28/2015	11/3/2015	
02-035	Urachal adenocarcinoma	TP53_R174S	CMS400	CMS400_01062016	Biopsy-lung	4/24/2015	5/15/2015	11/16/2015	
02-035	Urachal adenocarcinoma	CSMD3_R1021C	CMS400	CMS400_01062016	Biopsy-lung	4/24/2015	5/15/2015	11/16/2015	
02-035	Urachal adenocarcinoma	MAP2K1_G128D	CMS400	CMS400_01062016	Biopsy-lung	4/24/2015	5/15/2015	11/16/2015	
02-035	Urachal adenocarcinoma	ITGA9_A671D	CMS400	CMS400_01062016	Biopsy-lung	4/24/2015	5/15/2015	11/16/2015	
02-035	Urachal adenocarcinoma	SH2D1A_R78W	CMS400	CMS400_01062016	Biopsy-lung	4/24/2015	5/15/2015	11/16/2015	
02-035	Urachal adenocarcinoma	ZNF521_S367N	CMS400	CMS400_01062016	Biopsy-lung	4/24/2015	5/15/2015	11/16/2015	
02-035	Urachal adenocarcinoma	PALB2_R170fs*14	FoundationOne	FoundationOne_315/28 Aug 2014 - Dec 2017	Bladder	1/15/2010	8/12/2015	11/16/2015	2
02-035	Urachal adenocarcinoma	TP53_R174S	FoundationOne	FoundationOne_315/28 Aug 2014 - Dec 2017	Bladder	1/15/2010	8/12/2015	11/16/2015	
02-035	Urachal adenocarcinoma	LZTR1_Truncation_Exon_9	FoundationOne	FoundationOne_315/28 Aug 2014 - Dec 2017	Bladder	1/15/2010	8/12/2015	11/16/2015	
02-035	Urachal adenocarcinoma	MAP2K4_A43fs*35	FoundationOne	FoundationOne_315/28 Aug 2014 - Dec 2017	Bladder	1/15/2010	8/12/2015	11/16/2015	
02-035	Urachal adenocarcinoma	ARID1B_V227P	FoundationOne	FoundationOne_315/28 Aug 2014 - Dec 2017	Bladder	1/15/2010	8/12/2015	11/16/2015	
02-035	Urachal adenocarcinoma	ERBB3_R1077W	FoundationOne	FoundationOne_315/28 Aug 2014 - Dec 2017	Bladder	1/15/2010	8/12/2015	11/16/2015	
02-035	Urachal adenocarcinoma	GABRA6_D418H	FoundationOne	FoundationOne_315/28 Aug 2014 - Dec 2017	Bladder	1/15/2010	8/12/2015	11/16/2015	
02-035	Urachal adenocarcinoma	MAP2K1_G128D	FoundationOne	FoundationOne_315/28 Aug 2014 - Dec 2017	Bladder	1/15/2010	8/12/2015	11/16/2015	
02-035	Urachal adenocarcinoma	MPL_Y631S	FoundationOne	FoundationOne_315/28 Aug 2014 - Dec 2017	Bladder	1/15/2010	8/12/2015	11/16/2015	
02-035	Urachal adenocarcinoma	MSH6_E264_E271>E	FoundationOne	FoundationOne_315/28 Aug 2014 - Dec 2017	Bladder	1/15/2010	8/12/2015	11/16/2015	
02-035	Urachal adenocarcinoma	MYST3_L683F	FoundationOne	FoundationOne_315/28 Aug 2014 - Dec 2017	Bladder	1/15/2010	8/12/2015	11/16/2015	
02-035	Urachal adenocarcinoma	RANBP2_G2692D	FoundationOne	FoundationOne_315/28 Aug 2014 - Dec 2017	Bladder	1/15/2010	8/12/2015	11/16/2015	
02-035	Urachal adenocarcinoma	SPTA1_A125T	FoundationOne	FoundationOne_315/28 Aug 2014 - Dec 2017	Bladder	1/15/2010	8/12/2015	11/16/2015	
02-035	Urachal adenocarcinoma	TSC1_D650E	FoundationOne	FoundationOne_315/28 Aug 2014 - Dec 2017	Bladder	1/15/2010	8/12/2015	11/16/2015	
02-041	Adenocarcinoma of pancreas	ATM_R3047*	Guardant360	Guardant360_73Gene Nov2016 - Mar2019	Blood	6/15/2017	7/3/2017	5/9/2018	2
02-041	Adenocarcinoma of pancreas	KRAS_G12D	Guardant360	Guardant360_73Gene Nov2016 - Mar2019	Blood	6/15/2017	7/3/2017	5/9/2018	
02-041	Adenocarcinoma of pancreas	ATM_S381fs*27	Invitae Multi-Cancer Panel	INVITAE Multi-Cancer Panel_12212021	Blood	6/14/2017	7/5/2017	5/9/2018	
02-042	Endometrioid adenocarcinoma	NOTCH1_P1730L	STGA-DNA 2018	STGA-DNA 2018_11012021	PERITONEUM	2/13/2018	3/16/2018	5/1/2018	
02-042	Endometrioid adenocarcinoma	PTEN_H61fs*2	STGA-DNA 2018	STGA-DNA 2018_11012021	PERITONEUM	2/13/2018	3/16/2018	5/1/2018	
02-042	Endometrioid adenocarcinoma	ARID1A_P146fs*86	STGA-DNA 2018	STGA-DNA 2018_11012021	PERITONEUM	2/13/2018	3/16/2018	5/1/2018	
02-042	Endometrioid adenocarcinoma	MED12_K1225fs*74	STGA-DNA 2018	STGA-DNA 2018_11012021	PERITONEUM	2/13/2018	3/16/2018	5/1/2018	
02-042	Endometrioid adenocarcinoma	PTEN_L70_E73del	STGA-DNA 2018	STGA-DNA 2018_11012021	PERITONEUM	2/13/2018	3/16/2018	5/1/2018	
02-042	Endometrioid adenocarcinoma	MYD88_I266fs*166	STGA-DNA 2018	STGA-DNA 2018_11012021	PERITONEUM	2/13/2018	3/16/2018	5/1/2018	
02-042	Endometrioid adenocarcinoma	ARID1A_S1992L	STGA-DNA 2018	STGA-DNA 2018_11012021	PERITONEUM	2/13/2018	3/16/2018	5/1/2018	1
02-042	Endometrioid adenocarcinoma	FANCA_P1218L	STGA-DNA 2018	STGA-DNA 2018_11012021	PERITONEUM	2/13/2018	3/16/2018	5/1/2018	
02-042	Endometrioid adenocarcinoma	ESR1_H196R	STGA-DNA 2018	STGA-DNA 2018_11012021	PERITONEUM	2/13/2018	3/16/2018	5/1/2018	
02-042	Endometrioid adenocarcinoma	MYC_R393C	STGA-DNA 2018	STGA-DNA 2018_11012021	PERITONEUM	2/13/2018	3/16/2018	5/1/2018	
02-043	Colon adenocarcinoma (crc)	RB1_Amplification	FoundationOne	FoundationOne_315/28 Aug 2014 - Dec 2017	Lung	5/31/2017	6/13/2017	5/17/2018	
02-043	Colon adenocarcinoma (crc)	FLT1_Amplification	FoundationOne	FoundationOne_315/28 Aug 2014 - Dec 2017	Lung	5/31/2017	6/13/2017	5/17/2018	
02-043	Colon adenocarcinoma (crc)	CDK8_Amplification	FoundationOne	FoundationOne_315/28 Aug 2014 - Dec 2017	Lung	5/31/2017	6/13/2017	5/17/2018	
02-043	Colon adenocarcinoma (crc)	FGF14_Amplification	FoundationOne	FoundationOne_315/28 Aug 2014 - Dec 2017	Lung	5/31/2017	6/13/2017	5/17/2018	
02-043	Colon adenocarcinoma (crc)	KRAS_G12C	FoundationOne	FoundationOne_315/28 Aug 2014 - Dec 2017	Lung	5/31/2017	6/13/2017	5/17/2018	
02-043	Colon adenocarcinoma (crc)	APC_G267*	FoundationOne	FoundationOne_315/28 Aug 2014 - Dec 2017	Lung	5/31/2017	6/13/2017	5/17/2018	
02-043	Colon adenocarcinoma (crc)	APC_Q1378*	FoundationOne	FoundationOne_315/28 Aug 2014 - Dec 2017	Lung	5/31/2017	6/13/2017	5/17/2018	
02-043	Colon adenocarcinoma (crc)	GPR124_R405H	FoundationOne	FoundationOne_315/28 Aug 2014 - Dec 2017	Lung	5/31/2017	6/13/2017	5/17/2018	
02-043	Colon adenocarcinoma (crc)	SOX9_P374fs*9	FoundationOne	FoundationOne_315/28 Aug 2014 - Dec 2017	Lung	5/31/2017	6/13/2017	5/17/2018	
02-043	Colon adenocarcinoma (crc)	TP53_P191del	FoundationOne	FoundationOne_315/28 Aug 2014 - Dec 2017	Lung	5/31/2017	6/13/2017	5/17/2018	
02-043	Colon adenocarcinoma (crc)	ATM_T2333K	FoundationOne	FoundationOne_315/28 Aug 2014 - Dec 2017	Lung	5/31/2017	6/13/2017	5/17/2018	2
02-043	Colon adenocarcinoma (crc)	CDK12_D247E	FoundationOne	FoundationOne_315/28 Aug 2014 - Dec 2017	Lung	5/31/2017	6/13/2017	5/17/2018	
02-043	Colon adenocarcinoma (crc)	GATA6_N421fs*48	FoundationOne	FoundationOne_315/28 Aug 2014 - Dec 2017	Lung	5/31/2017	6/13/2017	5/17/2018	
02-043	Colon adenocarcinoma (crc)	MSH6_L76P	FoundationOne	FoundationOne_315/28 Aug 2014 - Dec 2017	Lung	5/31/2017	6/13/2017	5/17/2018	
02-043	Colon adenocarcinoma (crc)	SDHA_D49G	FoundationOne	FoundationOne_315/28 Aug 2014 - Dec 2017	Lung	5/31/2017	6/13/2017	5/17/2018	
02-043	Colon adenocarcinoma (crc)	SPEN_E1551D	FoundationOne	FoundationOne_315/28 Aug 2014 - Dec 2017	Lung	5/31/2017	6/13/2017	5/17/2018	
02-045	Synovial sarcoma	PALB2_Q552*	Perthera	Precision	Retroperitoneum	12/28/2015	1/19/2018	6/8/2018	2
02-045	Synovial sarcoma	SS18-SSX1	Perthera	Precision	Retroperitoneum	12/28/2015	1/19/2018	6/8/2018	
02-045	Synovial sarcoma	CBLC_P435S	LMPv1	EndLeukemia Mutation Panel V1_11052021	Bone marrow	4/22/2019	4/25/2019	6/8/2018	
02-046	Cholangiocarcinoma	TP53_R175H	NCI-MATCH NGS assay	NCI-MATCH NGS assay V1.2	Other	11/4/2015	12/18/2015	6/6/2018	
02-046	Cholangiocarcinoma	MET_S1141L	Guardant360	Guardant360_54Gene_Jun2014-Jan2015	Blood	2/13/2018	11/29/2016	6/6/2018	
02-046	Cholangiocarcinoma	TP53_R175H	Guardant360	Guardant360_73Gene Nov2016 - Mar2019	Blood	2/13/2018	2/22/2018	6/6/2018	
02-046	Cholangiocarcinoma	TP53_Y220C	Guardant360	Guardant360_73Gene Nov2016 - Mar2019	Blood	2/13/2018	2/22/2018	6/6/2018	
02-046	Cholangiocarcinoma	MET_S1159L	Guardant360	Guardant360_73Gene Nov2016 - Mar2019	Blood	2/13/2018	2/22/2018	6/6/2018	
02-046	Cholangiocarcinoma	MET_S1141L	Guardant360	Guardant360_73Gene Nov2016 - Mar2019	Blood	2/13/2018	2/22/2018	6/6/2018	
02-046	Cholangiocarcinoma	BRAF_V600E	Guardant360	Guardant360_73Gene Nov2016 - Mar2019	blood	2/13/2018	2/22/2018	6/6/2018	
02-046	Cholangiocarcinoma	FGFR2_N549K	Guardant360	Guardant360_73Gene Nov2016 - Mar2019	blood	2/13/2018	2/22/2018	6/6/2018	
02-046	Cholangiocarcinoma	FGFR2_V564I	Guardant360	Guardant360_73Gene Nov2016 - Mar2019	blood	2/13/2018	2/22/2018	6/6/2018	
02-046	Cholangiocarcinoma	FGFR1_R254W	FoundationOne	FoundationOne_315/28 Aug 2014 - Dec 2017	Abdomen	3/6/2018	4/6/2018	6/6/2018	
02-046	Cholangiocarcinoma	FGFR2-BICC1	FoundationOne	FoundationOne_315/28 Aug 2014 - Dec 2017	Abdomen	3/6/2018	4/6/2018	6/6/2018	
02-046	Cholangiocarcinoma	FGFR2_N549K	FoundationOne	FoundationOne_315/28 Aug 2014 - Dec 2017	Abdomen	3/6/2018	4/6/2018	6/6/2018	
02-046	Cholangiocarcinoma	FGFR2_V564L	FoundationOne	FoundationOne_315/28 Aug 2014 - Dec 2017	Abdomen	3/6/2018	4/6/2018	6/6/2018	
02-046	Cholangiocarcinoma	PTEN_c.209+1G>A	FoundationOne	FoundationOne_315/28 Aug 2014 - Dec 2017	Abdomen	3/6/2018	4/6/2018	6/6/2018	
02-046	Cholangiocarcinoma	SRC_R271W	FoundationOne	FoundationOne_315/28 Aug 2014 - Dec 2017	Abdomen	3/6/2018	4/6/2018	6/6/2018	
02-046	Cholangiocarcinoma	TP53_R175H	FoundationOne	FoundationOne_315/28 Aug 2014 - Dec 2017	Abdomen	3/6/2018	4/6/2018	6/6/2018	
02-046	Cholangiocarcinoma	GABRA6_S399L	FoundationOne	FoundationOne_315/28 Aug 2014 - Dec 2017	Abdomen	3/6/2018	4/6/2018	6/6/2018	
02-046	Cholangiocarcinoma	PBRM1_c.529-1G>T	FoundationOne	FoundationOne_315/28 Aug 2014 - Dec 2017	Abdomen	3/6/2018	4/6/2018	6/6/2018	
02-046	Cholangiocarcinoma	PDGFRB_S55L	FoundationOne	FoundationOne_315/28 Aug 2014 - Dec 2017	Abdomen	3/6/2018	4/6/2018	6/6/2018	
02-046	Cholangiocarcinoma	SMARCA4_Q1185E	FoundationOne	FoundationOne_315/28 Aug 2014 - Dec 2017	Abdomen	3/6/2018	4/6/2018	6/6/2018	
02-046	Cholangiocarcinoma	BARD1_S551*	FoundationOne	FoundationOne_315/28 Aug 2014 - Dec 2017	Abdomen	3/6/2018	4/6/2018	6/6/2018	1
02-046	Cholangiocarcinoma	CIC_R30C	FoundationOne	FoundationOne_315/28 Aug 2014 - Dec 2017	Abdomen	3/6/2018	4/6/2018	6/6/2018	
02-046	Cholangiocarcinoma	DOT1L_R319G	FoundationOne	FoundationOne_315/28 Aug 2014 - Dec 2017	Abdomen	3/6/2018	4/6/2018	6/6/2018	
02-046	Cholangiocarcinoma	MUTYH_G382D	FoundationOne	FoundationOne_315/28 Aug 2014 - Dec 2017	Abdomen	3/6/2018	4/6/2018	6/6/2018	
02-046	Cholangiocarcinoma	MAGI2_G1304D	FoundationOne	FoundationOne_315/28 Aug 2014 - Dec 2017	Abdomen	3/6/2018	4/6/2018	6/6/2018	
02-046	Cholangiocarcinoma	FGFR2_Amplification	FoundationOne	FoundationOne_182/14 -- Dec 2012	Liver	3/30/2012	7/16/2012	6/6/2018	
02-046	Cholangiocarcinoma	FGFR2_Amplification	Guardant360	Guardant360_73Gene Nov2016 - Mar2019	Blood	2/13/2018	2/22/2018	6/6/2018	
02-046	Cholangiocarcinoma	CIC_Amplification	FoundationOne	FoundationOne_315/28 Aug 2014 - Dec 2017	Abdomen	3/6/2018	4/6/2018	6/6/2018	
02-050	Cholangiocarcinoma	FGFR2-WAC	FoundationOne	FoundationOne_315/28 Aug 2014 - Dec 2017	Adrenal Gland	3/29/2017	6/29/2017	7/12/2018	
02-050	Cholangiocarcinoma	BAP1_P522fs*13	FoundationOne	FoundationOne_315/28 Aug 2014 - Dec 2017	Adrenal Gland	3/29/2017	6/29/2017	7/12/2018	3
02-050	Cholangiocarcinoma	MUTYH_G382D	FoundationOne	FoundationOne_315/28 Aug 2014 - Dec 2017	Adrenal Gland	3/29/2017	6/29/2017	7/12/2018	
02-050	Cholangiocarcinoma	POLE_R446Q	FoundationOne	FoundationOne_315/28 Aug 2014 - Dec 2017	Adrenal Gland	3/29/2017	6/29/2017	7/12/2018	
02-050	Cholangiocarcinoma	CSF1R_E920D	FoundationOne	FoundationOne_315/28 Aug 2014 - Dec 2017	Adrenal Gland	3/29/2017	6/29/2017	7/12/2018	
02-050	Cholangiocarcinoma	MLL_P10S	FoundationOne	FoundationOne_315/28 Aug 2014 - Dec 2017	Adrenal Gland	3/29/2017	6/29/2017	7/12/2018	
02-050	Cholangiocarcinoma	PRDM1_K188E	FoundationOne	FoundationOne_315/28 Aug 2014 - Dec 2017	Adrenal Gland	3/29/2017	6/29/2017	7/12/2018	
02-050	Cholangiocarcinoma	TSC2_L826M	FoundationOne	FoundationOne_315/28 Aug 2014 - Dec 2017	Adrenal Gland	3/29/2017	6/29/2017	7/12/2018	
02-050	Cholangiocarcinoma	PBRM1_L542F	FoundationOne	FoundationOne_315/28 Aug 2014 - Dec 2017	Adrenal Gland	3/29/2017	6/29/2017	7/12/2018	
02-050	Cholangiocarcinoma	CDKN2A_Deletion	FoundationOne	FoundationOne_315/28 Aug 2014 - Dec 2017	Adrenal Gland	3/29/2017	6/29/2017	7/12/2018	
02-050	Cholangiocarcinoma	CDKN2B_Deletion	FoundationOne	FoundationOne_315/28 Aug 2014 - Dec 2017	Adrenal Gland	3/29/2017	6/29/2017	7/12/2018	
02-051	Adenocarcinoma of unknown primary	ARID1A_R1989*	The Jackson Laboratory	ActionSeq Plus	Right pleura	8/31/2017	3/13/2018	7/26/2018	1
02-051	Adenocarcinoma of unknown primary	PIK3CA_M1043I	The Jackson Laboratory	ActionSeq Plus	Right pleura	8/31/2017	3/13/2018	7/26/2018	
02-051	Adenocarcinoma of unknown primary	TP53_Q167*	The Jackson Laboratory	ActionSeq Plus	Right pleura	8/31/2017	3/13/2018	7/26/2018	
02-051	Adenocarcinoma of unknown primary	ATM_V410A	The Jackson Laboratory	ActionSeq Plus	Right pleura	8/31/2017	3/13/2018	7/26/2018	
02-051	Adenocarcinoma of unknown primary	ATR_K764E	The Jackson Laboratory	ActionSeq Plus	Right pleura	8/31/2017	3/13/2018	7/26/2018	
02-051	Adenocarcinoma of unknown primary	CDK8_Y353_P354del	The Jackson Laboratory	ActionSeq Plus	Right pleura	8/31/2017	3/13/2018	7/26/2018	
02-051	Adenocarcinoma of unknown primary	FANCL_T372fs*23	The Jackson Laboratory	ActionSeq Plus	Right pleura	8/31/2017	3/13/2018	7/26/2018	
02-051	Adenocarcinoma of unknown primary	GRM3_R290C	The Jackson Laboratory	ActionSeq Plus	Right pleura	8/31/2017	3/13/2018	7/26/2018	
02-051	Adenocarcinoma of unknown primary	NF1_D2606V	The Jackson Laboratory	ActionSeq Plus	Right pleura	8/31/2017	3/13/2018	7/26/2018	
02-051	Adenocarcinoma of unknown primary	NOTCH2_H2056R	The Jackson Laboratory	ActionSeq Plus	Right pleura	8/31/2017	3/13/2018	7/26/2018	
02-054	Appendiceal cancer	SMAD4_G247*	STGAv1	STGAv1_03132018	Soft Tissue, RETROPYLORIC TUMOR	11/27/2017	1/18/2018	8/14/2018	
02-054	Appendiceal cancer	GNAS_R201H	STGAv1	STGAv1_03132018	Soft Tissue, RETROPYLORIC TUMOR	11/27/2017	1/18/2018	8/14/2018	
02-054	Appendiceal cancer	KRAS_G12D	STGAv1	STGAv1_03132018	Soft Tissue, RETROPYLORIC TUMOR	11/27/2017	1/18/2018	8/14/2018	
02-054	Appendiceal cancer	ATM_S1599*	STGAv1	STGAv1_03132018	Soft Tissue, RETROPYLORIC TUMOR	11/27/2017	1/18/2018	8/14/2018	2

02-055	Adenocarinoma of rectum	NRAS_Q61R	STGA-DNA 2018	STGA-DNA 2018_11012021	Liver	1/8/2018	5/11/2018	8/23/2018	
02-055	Adenocarinoma of rectum	ATM_Y2791*	STGA-DNA 2018	STGA-DNA 2018_11012021	Liver	1/8/2018	5/11/2018	8/23/2018	0
02-055	Adenocarinoma of rectum	PIK3CA_E81K	STGA-DNA 2018	STGA-DNA 2018_11012021	Liver	1/8/2018	5/11/2018	8/23/2018	
02-055	Adenocarinoma of rectum	TP53_R306*	STGA-DNA 2018	STGA-DNA 2018_11012021	Liver	1/8/2018	5/11/2018	8/23/2018	
02-057	Cholangiocarcinoma	CSF1R_R549C	FoundationOne	FoundationOne_315/28 Aug 2014 - Dec 2017	Liver	7/25/2016	7/10/2017	9/11/2018	
02-057	Cholangiocarcinoma	HNF1A_H483R	FoundationOne	FoundationOne_315/28 Aug 2014 - Dec 2017	Liver	7/25/2016	7/10/2017	9/11/2018	
02-057	Cholangiocarcinoma	IDH1_R132C	FoundationOne	FoundationOne_315/28 Aug 2014 - Dec 2017	Liver	7/25/2016	7/10/2017	9/11/2018	
02-057	Cholangiocarcinoma	ARID1A_G365fs*27	FoundationOne	FoundationOne_315/28 Aug 2014 - Dec 2017	Liver	7/25/2016	7/10/2017	9/11/2018	4
02-057	Cholangiocarcinoma	PBRM1_Y1128C	FoundationOne	FoundationOne_315/28 Aug 2014 - Dec 2017	Liver	7/25/2016	7/10/2017	9/11/2018	
02-057	Cholangiocarcinoma	AXL_Q361P	FoundationOne	FoundationOne_315/28 Aug 2014 - Dec 2017	Liver	7/25/2016	7/10/2017	9/11/2018	
02-057	Cholangiocarcinoma	CTNNA1_R546G	FoundationOne	FoundationOne_315/28 Aug 2014 - Dec 2017	Liver	7/25/2016	7/10/2017	9/11/2018	
02-057	Cholangiocarcinoma	SF3B1-ZAN	FoundationOne	FoundationOne_315/28 Aug 2014 - Dec 2017	Liver	7/25/2016	7/10/2017	9/11/2018	
02-057	Cholangiocarcinoma	ZBTB2_L222M	FoundationOne	FoundationOne_315/28 Aug 2014 - Dec 2017	Liver	7/25/2016	7/10/2017	9/11/2018	
02-058	Rectal cancer	FLT3_Amplification	STGA-DNA 2018	STGA-DNA 2018_11012021	Portion of sigmoid, rectum, anus	7/25/2017	5/17/2018	9/20/2018	
02-058	Rectal cancer	FANCD2_R1392Q	STGA-DNA 2018	STGA-DNA 2018_11012021	Portion of sigmoid, rectum, anus	7/25/2017	5/17/2018	9/20/2018	
02-058	Rectal cancer	TSC2_E1552del	STGA-DNA 2018	STGA-DNA 2018_11012021	Portion of sigmoid, rectum, anus	7/25/2017	5/17/2018	9/20/2018	
02-058	Rectal cancer	TSC2_c.4569+1G>C	STGA-DNA 2018	STGA-DNA 2018_11012021	Portion of sigmoid, rectum, anus	7/25/2017	5/17/2018	9/20/2018	
02-058	Rectal cancer	ATR_D604fs*3	STGA-DNA 2018	STGA-DNA 2018_11012021	Portion of sigmoid, rectum, anus	7/25/2017	5/17/2018	9/20/2018	1
02-058	Rectal cancer	ATR_M593_P597delinsK	STGA-DNA 2018	STGA-DNA 2018_11012021	Portion of sigmoid, rectum, anus	7/25/2017	5/17/2018	9/20/2018	
02-058	Rectal cancer	TP53_R273H	STGA-DNA 2018	STGA-DNA 2018_11012021	Portion of sigmoid, rectum, anus	7/25/2017	5/17/2018	9/20/2018	
02-058	Rectal cancer	FBXW7_W606*	STGA-DNA 2018	STGA-DNA 2018_11012021	Portion of sigmoid, rectum, anus	7/25/2017	5/17/2018	9/20/2018	
02-059	Ovarian clear cell carcinoma	ARID1A_P1175fs	MI Profile	592 Gene Panel, Nov 2015-Jun 2021	Liver	8/18/2017	9/11/2017	9/11/2018	3
02-059	Ovarian clear cell carcinoma	ARID1A_M1634fs	MI Profile	592 Gene Panel, Nov 2015-Jun 2021	Liver	8/18/2017	9/11/2017	9/11/2018	
02-059	Ovarian clear cell carcinoma	MAX_H28R	MI Profile	592 Gene Panel, Nov 2015-Jun 2021	Liver	8/18/2017	9/11/2017	9/11/2018	
02-059	Ovarian clear cell carcinoma	PIK3CA_Q546R	MI Profile	592 Gene Panel, Nov 2015-Jun 2021	Liver	8/18/2017	9/11/2017	9/11/2018	
02-059	Ovarian clear cell carcinoma	BMPR1A_E415K	MI Profile	592 Gene Panel, Nov 2015-Jun 2021	Liver	8/18/2017	9/11/2017	9/11/2018	
02-059	Ovarian clear cell carcinoma	CBFA2T3_V602M	MI Profile	592 Gene Panel, Nov 2015-Jun 2021	Liver	8/18/2017	9/11/2017	9/11/2018	
02-059	Ovarian clear cell carcinoma	CDH11_S271R	MI Profile	592 Gene Panel, Nov 2015-Jun 2021	Liver	8/18/2017	9/11/2017	9/11/2018	
02-059	Ovarian clear cell carcinoma	CHEK2_P128L	MI Profile	592 Gene Panel, Nov 2015-Jun 2021	Liver	8/18/2017	9/11/2017	9/11/2018	
02-059	Ovarian clear cell carcinoma	DOT1L_G1392S	MI Profile	592 Gene Panel, Nov 2015-Jun 2021	Liver	8/18/2017	9/11/2017	9/11/2018	
02-059	Ovarian clear cell carcinoma	EXT2_G662A	MI Profile	592 Gene Panel, Nov 2015-Jun 2021	Liver	8/18/2017	9/11/2017	9/11/2018	
02-059	Ovarian clear cell carcinoma	IRS2_A701dup	MI Profile	592 Gene Panel, Nov 2015-Jun 2021	Liver	8/18/2017	9/11/2017	9/11/2018	
02-059	Ovarian clear cell carcinoma	MAP2K4_S16R	MI Profile	592 Gene Panel, Nov 2015-Jun 2021	Liver	8/18/2017	9/11/2017	9/11/2018	
02-059	Ovarian clear cell carcinoma	NOTCH1_G2126R	MI Profile	592 Gene Panel, Nov 2015-Jun 2021	Liver	8/18/2017	9/11/2017	9/11/2018	
02-059	Ovarian clear cell carcinoma	NUMA1_L81Q	MI Profile	592 Gene Panel, Nov 2015-Jun 2021	Liver	8/18/2017	9/11/2017	9/11/2018	
02-059	Ovarian clear cell carcinoma	PPARG_F275S	MI Profile	592 Gene Panel, Nov 2015-Jun 2021	Liver	8/18/2017	9/11/2017	9/11/2018	
02-059	Ovarian clear cell carcinoma	ROS1_V1970A	MI Profile	592 Gene Panel, Nov 2015-Jun 2021	Liver	8/18/2017	9/11/2017	9/11/2018	
02-062	Gastro-esophageal junction adenocarcinoma	ATM_Deletion	Tempus xT Assay	Tempus XT 648 Gene List	Lung, Right	5/23/2017	7/10/2018	10/26/2018	2
02-062	Gastro-esophageal junction adenocarcinoma	CDKN2A_Deletion	Tempus xT Assay	Tempus XT 648 Gene List	Lung, Right	5/23/2017	7/10/2018	10/26/2018	
02-062	Gastro-esophageal junction adenocarcinoma	APC_R499*	Tempus xT Assay	Tempus XT 648 Gene List	Lung, Right	5/23/2017	7/10/2018	10/26/2018	
02-062	Gastro-esophageal junction adenocarcinoma	CHD2_K1245fs	Tempus xT Assay	Tempus XT 648 Gene List	Lung, Right	5/23/2017	7/10/2018	10/26/2018	
02-062	Gastro-esophageal junction adenocarcinoma	KDM5C_c.2244-1G>A	Tempus xT Assay	Tempus XT 648 Gene List	Lung, Right	5/23/2017	7/10/2018	10/26/2018	
02-062	Gastro-esophageal junction adenocarcinoma	RAD50_K722fs	Tempus xT Assay	Tempus XT 648 Gene List	Lung, Right	5/23/2017	7/10/2018	10/26/2018	
02-062	Gastro-esophageal junction adenocarcinoma	SLC26A3_R312M	Tempus xT Assay	Tempus XT 648 Gene List	Lung, Right	5/23/2017	7/10/2018	10/26/2018	
02-062	Gastro-esophageal junction adenocarcinoma	SETD2_I1884T	Tempus xT Assay	Tempus XT 648 Gene List	Lung, Right	5/23/2017	7/10/2018	10/26/2018	
02-062	Gastro-esophageal junction adenocarcinoma	PHF6_S181Y	Tempus xT Assay	Tempus XT 648 Gene List	Lung, Right	5/23/2017	7/10/2018	10/26/2018	
02-062	Gastro-esophageal junction adenocarcinoma	CTCF_H373N	Tempus xT Assay	Tempus XT 648 Gene List	Lung, Right	5/23/2017	7/10/2018	10/26/2018	
02-062	Gastro-esophageal junction adenocarcinoma	SETD2_R1740W	Tempus xT Assay	Tempus XT 648 Gene List	Lung, Right	5/23/2017	7/10/2018	10/26/2018	
02-062	Gastro-esophageal junction adenocarcinoma	ALK_G1011A	Tempus xT Assay	Tempus XT 648 Gene List	Lung, Right	5/23/2017	7/10/2018	10/26/2018	
02-062	Gastro-esophageal junction adenocarcinoma	RUNX1T1_H318Q	Tempus xT Assay	Tempus XT 648 Gene List	Lung, Right	5/23/2017	7/10/2018	10/26/2018	
02-062	Gastro-esophageal junction adenocarcinoma	SOD2_C217*	Tempus xT Assay	Tempus XT 648 Gene List	Lung, Right	5/23/2017	7/10/2018	10/26/2018	
02-062	Gastro-esophageal junction adenocarcinoma	FLG_R1987S	Tempus xT Assay	Tempus XT 648 Gene List	Lung, Right	5/23/2017	7/10/2018	10/26/2018	
02-062	Gastro-esophageal junction adenocarcinoma	MLH3_G1248_R1251delinsGTKK	Tempus xT Assay	Tempus XT 648 Gene List	Lung, Right	5/23/2017	7/10/2018	10/26/2018	
02-062	Gastro-esophageal junction adenocarcinoma	INPP4B_Q737E	Tempus xT Assay	Tempus XT 648 Gene List	Lung, Right	5/23/2017	7/10/2018	10/26/2018	
02-062	Gastro-esophageal junction adenocarcinoma	IRF2_P22R	Tempus xT Assay	Tempus XT 648 Gene List	Lung, Right	5/23/2017	7/10/2018	10/26/2018	
02-062	Gastro-esophageal junction adenocarcinoma	ATM_E2052*	Tempus xT Assay	Tempus XT 648 Gene List	Lung, Right	5/23/2017	7/10/2018	10/26/2018	
02-062	Gastro-esophageal junction adenocarcinoma	ATM_L1322R	Tempus xT Assay	Tempus XT 648 Gene List	Lung, Right	5/23/2017	7/10/2018	10/26/2018	
02-062	Gastro-esophageal junction adenocarcinoma	BMPR1A_V474L	Tempus xT Assay	Tempus XT 648 Gene List	Lung, Right	5/23/2017	7/10/2018	10/26/2018	
02-062	Gastro-esophageal junction adenocarcinoma	APOB_L2898P	Tempus xT Assay	Tempus XT 648 Gene List	Lung, Right	5/23/2017	7/10/2018	10/26/2018	
02-063	Gallbladder adenocarcinoma	ATM_C2704fs*2	Guardant360	Guardant360_73Gene Nov2016 - Mar2019	Blood	4/24/2017	5/2/2017	10/4/2018	1
02-063	Gallbladder adenocarcinoma	EGFR_R977H	Guardant360	Guardant360_73Gene Nov2016 - Mar2019	Blood	4/24/2017	5/2/2017	10/4/2018	
02-063	Gallbladder adenocarcinoma	PDGFRA_N103fs*24	Guardant360	Guardant360_73Gene Nov2016 - Mar2019	Blood	4/24/2017	5/2/2017	10/4/2018	
02-063	Gallbladder adenocarcinoma	PIK3CA_E545K	Guardant360	Guardant360_73Gene Nov2016 - Mar2019	Blood	4/24/2017	5/2/2017	10/4/2018	
02-063	Gallbladder adenocarcinoma	PTEN_D326H	Guardant360	Guardant360_73Gene Nov2016 - Mar2019	Blood	4/24/2017	5/2/2017	10/4/2018	
02-063	Gallbladder adenocarcinoma	RB1_R255*	Guardant360	Guardant360_73Gene Nov2016 - Mar2019	Blood	4/24/2017	5/2/2017	10/4/2018	
02-063	Gallbladder adenocarcinoma	RB1_Y606fs*47	Guardant360	Guardant360_73Gene Nov2016 - Mar2019	Blood	4/24/2017	5/2/2017	10/4/2018	
02-063	Gallbladder adenocarcinoma	SMAD4_R361H	Guardant360	Guardant360_73Gene Nov2016 - Mar2019	Blood	4/24/2017	5/2/2017	10/4/2018	
02-063	Gallbladder adenocarcinoma	TP53_R175H	Guardant360	Guardant360_73Gene Nov2016 - Mar2019	Blood	4/24/2017	5/2/2017	10/4/2018	
02-063	Gallbladder adenocarcinoma	NF1_R440*	Guardant360	Guardant360_73Gene Nov2016 - Mar2019	Blood	4/24/2017	5/2/2017	10/4/2018	
02-064	Squamous cell carcinoma oropharynx	RAD51D_E14K	STGA-DNA 2018	STGA-DNA 2018_11012021	Left neck	8/4/2017	2/1/2018	10/3/2018	
02-064	Squamous cell carcinoma oropharynx	FANCA_W298*	STGA-DNA 2018	STGA-DNA 2018_11012021	Left neck	8/4/2017	2/1/2018	10/3/2018	1
02-064	Squamous cell carcinoma oropharynx	AKT1_E17K	STGA-DNA 2018	STGA-DNA 2018_11012021	Left neck	8/4/2017	2/1/2018	10/3/2018	
02-064	Squamous cell carcinoma oropharynx	NOTCH2_C471fs*2	STGA-DNA 2018	STGA-DNA 2018_11012021	Left neck	8/4/2017	2/1/2018	10/3/2018	
02-064	Squamous cell carcinoma oropharynx	POLE_E2140K	STGA-DNA 2018	STGA-DNA 2018_11012021	Left neck	8/4/2017	2/1/2018	10/3/2018	
02-066	Ampullar of Vater carcinoma	FBXW7_R465H	MI Tumor Seek	592 Gene panel, Nov 2015- Jun 2021	Duodenum	2/29/2016	6/27/2017	11/2/2018	
02-066	Ampullar of Vater carcinoma	MET_H633L	MI Tumor Seek	592 Gene panel, Nov 2015- Jun 2021	Duodenum	2/29/2016	6/27/2017	11/2/2018	
02-066	Ampullar of Vater carcinoma	ARID1A_R1289fs	MI Tumor Seek	592 Gene panel, Nov 2015- Jun 2021	Duodenum	2/29/2016	6/27/2017	11/2/2018	2
02-066	Ampullar of Vater carcinoma	TP53_D391fs	MI Tumor Seek	592 Gene panel, Nov 2015- Jun 2021	Duodenum	2/29/2016	6/27/2017	11/2/2018	
02-066	Ampullar of Vater carcinoma	MET_H633L	Invitae Multi-Cancer Panel	INVITAE Multi-Cancer Panel_12212021	Blood	9/22/2016	4/5/2016	11/2/2018	
02-066	Ampullar of Vater carcinoma	PALB2_L763F	Invitae Multi-Cancer Panel	INVITAE Multi-Cancer Panel_12212021	Blood	9/22/2016	4/5/2016	11/2/2018	
02-070	Head & Neck (squamous cell carcinoma)	PALB2_Q60fs*7	FoundationOne CDx	FoundationOne CDx_324/36 Dec 2017- Present	Soft tissue	4/30/2018	12/18/2018	1/11/2019	2
02-070	Head & Neck (squamous cell carcinoma)	BRD4_P252R	FoundationOne CDx	FoundationOne CDx_324/36 Dec 2017- Present	Soft tissue	4/30/2018	12/18/2018	1/11/2019	
02-070	Head & Neck (squamous cell carcinoma)	PDGFRB_R502W	FoundationOne CDx	FoundationOne CDx_324/36 Dec 2017- Present	Soft tissue	4/30/2018	12/18/2018	1/11/2019	
02-070	Head & Neck (squamous cell carcinoma)	CASP8_L287F	FoundationOne CDx	FoundationOne CDx_324/36 Dec 2017- Present	Soft tissue	4/30/2018	12/18/2018	1/11/2019	
02-070	Head & Neck (squamous cell carcinoma)	RARA_E176Q	FoundationOne CDx	FoundationOne CDx_324/36 Dec 2017- Present	Soft tissue	4/30/2018	12/18/2018	1/11/2019	
02-070	Head & Neck (squamous cell carcinoma)	DDR1_R105Q	FoundationOne CDx	FoundationOne CDx_324/36 Dec 2017- Present	Soft tissue	4/30/2018	12/18/2018	1/11/2019	
02-070	Head & Neck (squamous cell carcinoma)	JAK2_S797C	FoundationOne CDx	FoundationOne CDx_324/36 Dec 2017- Present	Soft tissue	4/30/2018	12/18/2018	1/11/2019	
02-078	High grade serous ovarian	PALB2_Q1056*	MI Profile	592 Gene Panel, Nov 2015-Jun 2021	Uterus and cervix	2/8/2019	2/26/2019	8/24/2019	1
02-078	High grade serous ovarian	CDK12_P1190A	MI Profile	592 Gene Panel, Nov 2015-Jun 2021	Uterus and cervix	2/8/2019	2/26/2019	8/24/2019	
02-078	High grade serous ovarian	TP53_Y163S	MI Profile	592 Gene Panel, Nov 2015-Jun 2021	Uterus and cervix	2/8/2019	2/26/2019	8/24/2019	
02-078	High grade serous ovarian	ARAF_R326Q	MI Profile	592 Gene Panel, Nov 2015-Jun 2021	Uterus and cervix	2/8/2019	2/26/2019	8/24/2019	
02-078	High grade serous ovarian	AXIN1_A558T	MI Profile	592 Gene Panel, Nov 2015-Jun 2021	Uterus and cervix	2/8/2019	2/26/2019	8/24/2019	
02-078	High grade serous ovarian	BCL7A_P172Q	MI Profile	592 Gene Panel, Nov 2015-Jun 2021	Uterus and cervix	2/8/2019	2/26/2019	8/24/2019	
02-078	High grade serous ovarian	BLM_A1043D	MI Profile	592 Gene Panel, Nov 2015-Jun 2021	Uterus and cervix	2/8/2019	2/26/2019	8/24/2019	
02-078	High grade serous ovarian	CAMTA1_P1219A	MI Profile	592 Gene Panel, Nov 2015-Jun 2021	Uterus and cervix	2/8/2019	2/26/2019	8/24/2019	
02-078	High grade serous ovarian	CD79A_V44L	MI Profile	592 Gene Panel, Nov 2015-Jun 2021	Uterus and cervix	2/8/2019	2/26/2019	8/24/2019	
02-078	High grade serous ovarian	CREB3L1_E45K	MI Profile	592 Gene Panel, Nov 2015-Jun 2021	Uterus and cervix	2/8/2019	2/26/2019	8/24/2019	
02-078	High grade serous ovarian	DDX5_P28L	MI Profile	592 Gene Panel, Nov 2015-Jun 2021	Uterus and cervix	2/8/2019	2/26/2019	8/24/2019	
02-078	High grade serous ovarian	IL6ST_I335T	MI Profile	592 Gene Panel, Nov 2015-Jun 2021	Uterus and cervix	2/8/2019	2/26/2019	8/24/2019	
02-078	High grade serous ovarian	KMT2A_P3112S	MI Profile	592 Gene Panel, Nov 2015-Jun 2021	Uterus and cervix	2/8/2019	2/26/2019	8/24/2019	
02-078	High grade serous ovarian	MAFB_R48S	MI Profile	592 Gene Panel, Nov 2015-Jun 2021	Uterus and cervix	2/8/2019	2/26/2019	8/24/2019	
02-078	High grade serous ovarian	MAML2_R762S	MI Profile	592 Gene Panel, Nov 2015-Jun 2021	Uterus and cervix	2/8/2019	2/26/2019	8/24/2019	
02-078	High grade serous ovarian	MLLT3_S345P	MI Profile	592 Gene Panel, Nov 2015-Jun 2021	Uterus and cervix	2/8/2019	2/26/2019	8/24/2019	
02-078	High grade serous ovarian	NCOA4_R58L	MI Profile	592 Gene Panel, Nov 2015-Jun 2021	Uterus and cervix	2/8/2019	2/26/2019	8/24/2019	
02-078	High grade serous ovarian	NDRG1_T360_G369dup	MI Profile	592 Gene Panel, Nov 2015-Jun 2021	Uterus and cervix	2/8/2019	2/26/2019	8/24/2019	
02-078	High grade serous ovarian	NUMA1_R1885H	MI Profile	592 Gene Panel, Nov 2015-Jun 2021	Uterus and cervix	2/8/2019	2/26/2019	8/24/2019	
02-078	High grade serous ovarian	PPP2R1A_E100K	MI Profile	592 Gene Panel, Nov 2015-Jun 2021	Uterus and cervix	2/8/2019	2/26/2019	8/24/2019	
02-078	High grade serous ovarian	RECQL4_G387R	MI Profile	592 Gene Panel, Nov 2015-Jun 2021	Uterus and cervix	2/8/2019	2/26/2019	8/24/2019	
02-078	High grade serous ovarian	REL_Q535H	MI Profile	592 Gene Panel, Nov 2015-Jun 2021	Uterus and cervix	2/8/2019	2/26/2019	8/24/2019	
02-078	High grade serous ovarian	RNF213_V933I	MI Profile	592 Gene Panel, Nov 2015-Jun 2021	Uterus and cervix	2/8/2019	2/26/2019	8/24/2019	
02-078	High grade serous ovarian	SLC45A3_L113V	MI Profile	592 Gene Panel, Nov 2015-Jun 2021	Uterus and cervix	2/8/2019	2/26/2019	8/24/2019	
02-078	High grade serous ovarian	SPEN_T2983M	MI Profile	592 Gene Panel, Nov 2015-Jun 2021	Uterus and cervix	2/8/2019	2/26/2019	8/24/2019	
02-078	High grade serous ovarian	SYK_G595R	MI Profile	592 Gene Panel, Nov 2015-Jun 2021	Uterus and cervix	2/8/2019	2/26/2019	8/24/2019	
02-078	High grade serous ovarian	TAL1_A104L	MI Profile	592 Gene Panel, Nov 2015-Jun 2021	Uterus and cervix	2/8/2019	2/26/2019	8/24/2019	
02-078	High grade serous ovarian	TMPRSS2_A53S	MI Profile	592 Gene Panel, Nov 2015-Jun 2021	Uterus and cervix	2/8/2019	2/26/2019	8/24/2019	
02-078	High grade serous ovarian	TPR_V915A	MI Profile	592 Gene Panel, Nov 2015-Jun 2021	Uterus and cervix	2/8/2019	2/26/2019	8/24/2019	
02-078	High grade serous ovarian	USP6_M1404I	MI Profile	592 Gene Panel, Nov 2015-Jun 2021	Uterus and cervix	2/8/2019	2/26/2019	8/24/2019	
02-083	Leiomyosarcoma	TP53_C275Y	STGAv1	STGAv1_03132018	Biopsy- left adrenal	1/26/2016	6/29/2016	2/4/2020	
02-083	Leiomyosarcoma	RB1_Y173fs*2	STGAv1	STGAv1_03132018	Biopsy- left adrenal	1/26/2016	6/29/2016	2/4/2020	
02-083	Leiomyosarcoma	TP53_C275Y	Tempus xT Assay	Tempus XT 648 Gene List	Bone, right deep pelvic	10/28/2019	11/16/2019	2/4/2020	
02-083	Leiomyosarcoma	RB1_Y173fs*2	Tempus xT Assay	Tempus XT 648 Gene List	Bone, right deep pelvic	10/28/2019	11/16/2019	2/4/2020	
02-083	Leiomyosarcoma	FANCC_A471fs*47	Tempus xT Assay	Tempus XT 648 Gene List	Bone, right deep pelvic	10/28/2019	11/16/2019	2/4/2020	0
02-083	Leiomyosarcoma	B2M_Deletion	Tempus xT Assay	Tempus XT 648 Gene List	Bone, right deep pelvic	10/28/2019	11/16/2019	2/4/2020	
02-085	Renal cell carcinoma	FANCC_E310*	Tempus xT Assay	Tempus XT 648 Gene List	Kidney, left	5/29/2018	Unkown	2/21/2020	2
02-085	Renal cell carcinoma	KMT2D_Q2540*	Tempus xT Assay	Tempus XT 648 Gene List	Kidney, left	5/29/2018	Unkown	2/21/2020	
02-085	Renal cell carcinoma	MKI67_K3206fs*32	Tempus xT Assay	Tempus XT 648 Gene List	Kidney, left	5/29/2018	Unkown	2/21/2020	
02-085	Renal cell carcinoma	SRPRA_K170fs	Tempus xT Assay	Tempus XT 648 Gene List	Kidney, left	5/29/2018	Unkown	2/21/2020	
02-085	Renal cell carcinoma	PRSS1_R68C	Tempus xT Assay	Tempus XT 648 Gene List	Kidney, left	5/29/2018	Unkown	2/21/2020	
03-001	Colon adenocarcinoma (crc)	PTEN_K6fs*4	FoundationOne	FoundationOne_236/19 Dec 2012 - Aug 2014	Rectum	9/17/2013	5/30/2014	1/15/2015	4
03-001	colon adenocarcinoma (crc)	PTEN_T319fs*1	FoundationOne	FoundationOne_236/19 Dec 2012 - Aug 2014	Rectum	9/17/2013	5/30/2014	1/15/2015	
03-001	colon adenocarcinoma (crc)	EP300_E1025fs*63	FoundationOne	FoundationOne_236/19 Dec 2012 - Aug 2014	Rectum	9/17/2013	5/30/2014	1/15/2015	
03-001	colon adenocarcinoma (crc)	AXL_R471C	FoundationOne	FoundationOne_236/19 Dec 2012 - Aug 2014	Rectum	9/17/2013	5/30/2014	1/15/2015	
03-001	colon adenocarcinoma (crc)	IRS2_F783del	FoundationOne	FoundationOne_236/19 Dec 2012 - Aug 2014	Rectum	9/17/2013	5/30/2014	1/15/2015	
03-001	colon adenocarcinoma (crc)	JUN_K311fs*6	FoundationOne	FoundationOne_236/19 Dec 2012 - Aug 2014	Rectum	9/17/2013	5/30/2014	1/15/2015	
03-001	colon adenocarcinoma (crc)	JUN_T231fs*20	FoundationOne	FoundationOne_236/19 Dec 2012 - Aug 2014	Rectum	9/17/2013	5/30/2014	1/15/2015	
03-001	colon adenocarcinoma (crc)	MAP2K2_K61E	FoundationOne	FoundationOne_236/19 Dec 2012 - Aug 2014	Rectum	9/17/2013	5/30/2014	1/15/2015	
03-001	colon adenocarcinoma (crc)	SPEN_P542S	FoundationOne	FoundationOne_236/19 Dec 2012 - Aug 2014	Rectum	9/17/2013	5/30/2014	1/15/2015	
03-004	Breast adenocarcinoma	KIT_K499N	CMS50	CMS50_01042017	Liver	8/6/2014	8/15/2014	2/24/2015	
03-004	Breast adenocarcinoma	PTEN_K254fs*42	CMS50	CMS50_01042017	Liver	8/6/2014	8/15/2014	2/24/2015	2
03-004	Breast adenocarcinoma	TP53_S183*	CMS50	CMS50_01042017	Liver	8/6/2014	8/15/2014	2/24/2015	
03-004	Breast adenocarcinoma	TP53_P98S	CMS50	CMS50_01042017	Liver	8/6/2014	8/15/2014	2/24/2015	
03-005	Breast infiltrating ductal carcinoma	PTEN_deletion	IHC	IHC		8/25/2014	8/27/2014	3/11/2015	0
03-005	Breast infiltrating ductal carcinoma	FBXW7_F241L	STGAv1	STGAv1_03132018	Breast	11/19/2015	6/15/2016	3/10/2015	
03-005	Breast infiltrating ductal carcinoma	NOTCH1_G1543R	STGAv1	STGAv1_03132018	Breast	11/19/2015	6/15/2016	3/10/2015	
03-005	Breast infiltrating ductal carcinoma	TP53_I255T	STGAv1	STGAv1_03132018	Breast	11/19/2015	6/15/2016	3/10/2015	
03-008	Gallbladder adenocarcinoma	ALK_T16A	FoundationOne	FoundationOne_236/19 Dec 2012 - Aug 2014	Liver	3/26/2012	8/8/2013	3/14/2015	
03-008	Gallbladder adenocarcinoma	FGFR2_V496I	FoundationOne	FoundationOne_236/19 Dec 2012 - Aug 2014	Liver	3/26/2012	8/8/2013	3/14/2015	
03-008	Gallbladder adenocarcinoma	KIT_R248K	FoundationOne	FoundationOne_236/19 Dec 2012 - Aug 2014	Liver	3/26/2012	8/8/2013	3/14/2015	
03-008	Gallbladder adenocarcinoma	NOTCH1_R912W	FoundationOne	FoundationOne_236/19 Dec 2012 - Aug 2014	Liver	3/26/2012	8/8/2013	3/14/2015	

03-008	Gallbladder adenocarcinoma	ATR_D331G	FoundationOne	FoundationOne_236/19 Dec 2012 - Aug 2014	Liver	3/26/2012	8/8/2013	3/14/2015	
03-008	Gallbladder adenocarcinoma	ATRX_S549G	FoundationOne	FoundationOne_236/19 Dec 2012 - Aug 2014	Liver	3/26/2012	8/8/2013	3/14/2015	
03-008	Gallbladder adenocarcinoma	MSH2_I651F	FoundationOne	FoundationOne_236/19 Dec 2012 - Aug 2014	Liver	3/26/2012	8/8/2013	3/14/2015	
03-008	Gallbladder adenocarcinoma	NF1_P1867S	FoundationOne	FoundationOne_236/19 Dec 2012 - Aug 2014	Liver	3/26/2012	8/8/2013	3/14/2015	
03-008	Gallbladder adenocarcinoma	NOTCH4_C1191R	FoundationOne	FoundationOne_236/19 Dec 2012 - Aug 2014	Liver	3/26/2012	8/8/2013	3/14/2015	
03-008	Gallbladder adenocarcinoma	PTCH1_T1052M	FoundationOne	FoundationOne_236/19 Dec 2012 - Aug 2014	Liver	3/26/2012	8/8/2013	3/14/2015	
03-008	Gallbladder adenocarcinoma	SOS1_M684I	FoundationOne	FoundationOne_236/19 Dec 2012 - Aug 2014	Liver	3/26/2012	8/8/2013	3/14/2015	
03-008	Gallbladder adenocarcinoma	AKT2_D416Y	FoundationOne	FoundationOne_236/19 Dec 2012 - Aug 2014	Liver	3/26/2012	8/8/2013	3/14/2015	
03-008	Gallbladder adenocarcinoma	BLM_P707S	FoundationOne	FoundationOne_236/19 Dec 2012 - Aug 2014	Liver	3/26/2012	8/8/2013	3/14/2015	
03-008	Gallbladder adenocarcinoma	MYCL_P18R	FoundationOne	FoundationOne_236/19 Dec 2012 - Aug 2014	Liver	3/26/2012	8/8/2013	3/14/2015	
03-008	Gallbladder adenocarcinoma	NRAS_G12V	FoundationOne	FoundationOne_236/19 Dec 2012 - Aug 2014	Liver	3/26/2012	8/8/2013	3/14/2015	
03-008	Gallbladder adenocarcinoma	PIK3CA_E365K	FoundationOne	FoundationOne_236/19 Dec 2012 - Aug 2014	Liver	3/26/2012	8/8/2013	3/14/2015	
03-008	Gallbladder adenocarcinoma	PTEN_L70F	FoundationOne	FoundationOne_236/19 Dec 2012 - Aug 2014	Liver	3/26/2012	8/8/2013	3/14/2015	4
03-008	Gallbladder adenocarcinoma	TP53_Q144*	FoundationOne	FoundationOne_236/19 Dec 2012 - Aug 2014	Liver	3/26/2012	8/8/2013	3/14/2015	
03-008	Gallbladder adenocarcinoma	RB1_Q471*	FoundationOne	FoundationOne_236/19 Dec 2012 - Aug 2014	Liver	3/26/2012	8/8/2013	3/14/2015	
03-008	Gallbladder adenocarcinoma	AKT2_D416Y	FoundationOne	FoundationOne_236/19 Dec 2012 - Aug 2014	Liver	3/26/2012	8/8/2013	3/14/2015	
03-008	Gallbladder adenocarcinoma	EPHA5_C302F	FoundationOne	FoundationOne_236/19 Dec 2012 - Aug 2014	Liver	3/26/2012	8/8/2013	3/14/2015	
03-008	Gallbladder adenocarcinoma	FGFR2_V496I	FoundationOne	FoundationOne_236/19 Dec 2012 - Aug 2014	Liver	3/26/2012	8/8/2013	3/14/2015	
03-008	Gallbladder adenocarcinoma	HGF_M684I	FoundationOne	FoundationOne_236/19 Dec 2012 - Aug 2014	Liver	3/26/2012	8/8/2013	3/14/2015	
03-008	Gallbladder adenocarcinoma	KIT_R248K	FoundationOne	FoundationOne_236/19 Dec 2012 - Aug 2014	Liver	3/26/2012	8/8/2013	3/14/2015	
03-008	Gallbladder adenocarcinoma	MSH2_I651F	FoundationOne	FoundationOne_236/19 Dec 2012 - Aug 2014	Liver	3/26/2012	8/8/2013	3/14/2015	
03-008	Gallbladder adenocarcinoma	MYCL1_P18R	FoundationOne	FoundationOne_236/19 Dec 2012 - Aug 2014	Liver	3/26/2012	8/8/2013	3/14/2015	
03-008	Gallbladder adenocarcinoma	NF1_P1867S	FoundationOne	FoundationOne_236/19 Dec 2012 - Aug 2014	Liver	3/26/2012	8/8/2013	3/14/2015	
03-008	Gallbladder adenocarcinoma	NOTCH1_R912W	FoundationOne	FoundationOne_236/19 Dec 2012 - Aug 2014	Liver	3/26/2012	8/8/2013	3/14/2015	
03-008	Gallbladder adenocarcinoma	PTCH1_T1052M	FoundationOne	FoundationOne_236/19 Dec 2012 - Aug 2014	Liver	3/26/2012	8/8/2013	3/14/2015	
03-008	Gallbladder adenocarcinoma	ERBB2_Amplification	FoundationOne	FoundationOne_236/19 Dec 2012 - Aug 2014	Liver	3/26/2012	8/8/2013	3/14/2015	
03-008	Gallbladder adenocarcinoma	CDK12_Amplification	FoundationOne	FoundationOne_236/19 Dec 2012 - Aug 2014	Liver	3/26/2012	8/8/2013	3/14/2015	
03-008	Gallbladder adenocarcinoma	RARA_Amplification	FoundationOne	FoundationOne_236/19 Dec 2012 - Aug 2014	Liver	3/26/2012	8/8/2013	3/14/2015	
03-010	Uterine leiomyosarcoma	GNAS_R147S	FoundationOne	FoundationOne_236/19 Dec 2012 - Aug 2014	Uterus	8/18/2009	7/1/2013	3/27/2015	
03-010	Uterine leiomyosarcoma	TP53_P190L	FoundationOne	FoundationOne_236/19 Dec 2012 - Aug 2014	Uterus	8/18/2009	7/1/2013	3/27/2015	
03-010	Uterine leiomyosarcoma	DDR2_Rearrangement	FoundationOne	FoundationOne_236/19 Dec 2012 - Aug 2014	Uterus	8/18/2009	7/1/2013	3/27/2015	
03-010	Uterine leiomyosarcoma	LRP1B_H856Q	FoundationOne	FoundationOne_236/19 Dec 2012 - Aug 2014	Uterus	8/18/2009	7/1/2013	3/27/2015	
03-010	Uterine leiomyosarcoma	TNFAIP3_T647P	FoundationOne	FoundationOne_236/19 Dec 2012 - Aug 2014	Uterus	8/18/2009	7/1/2013	3/27/2015	
03-010	Uterine leiomyosarcoma	FANCD2_C758S	FoundationOne	FoundationOne_236/19 Dec 2012 - Aug 2014	Uterus	8/18/2009	7/1/2013	3/27/2015	
03-010	Uterine leiomyosarcoma	STAG2_I948V	FoundationOne	FoundationOne_236/19 Dec 2012 - Aug 2014	Uterus	8/18/2009	7/1/2013	3/27/2015	
03-010	Uterine leiomyosarcoma	PTEN_Deletion	FoundationOne	FoundationOne_236/19 Dec 2012 - Aug 2014	Uterus	8/18/2009	7/1/2013	3/27/2015	8
03-013	Nasopharyngeal carcinoma	PTEN_R335*	CMS50	CMS50_01042017	unknown	11/27/2012	11/1/2014	5/4/2015	4
03-015	Squamous cell carcinoma of right inguinal area	PIK3CA_E81K	CMS50	CMS50_01042017	Inguinal soft tissue	7/28/2014	3/10/2015	5/1/2015	
03-015	Squamous cell carcinoma of right inguinal area	PTEN_R130Q	CMS50	CMS50_01042017	Inguinal soft tissue	7/28/2014	3/10/2015	5/1/2015	2
03-015	Squamous cell carcinoma of right inguinal area	SMAD4_F119fs*4	CMS50	CMS50_01042017	Inguinal soft tissue	7/28/2014	3/10/2015	5/1/2015	
03-015	Squamous cell carcinoma of right inguinal area	SMAD4_D415fs*20	CMS50	CMS50_01042017	Inguinal soft tissue	7/28/2014	3/10/2015	5/1/2015	
03-016	Endometrioid adenocarcinoma	CTNNB1_S33Y	CMS50	CMS50_01042017	Biopsy-vaginal	10/7/2014	1/26/2015	5/1/2015	
03-016	Endometrioid adenocarcinoma	PTEN_G127R	CMS50	CMS50_01042017	Biopsy-vaginal	10/7/2014	1/26/2015	5/1/2015	
03-016	Endometrioid adenocarcinoma	PTEN_P244fs*12	CMS50	CMS50_01042017	Biopsy-vaginal	10/7/2014	1/26/2015	5/1/2015	2
03-017	Cholangiocarcinoma	MCL1_Amplification	FoundationOne	FoundationOne_236/19 Dec 2012 - Aug 2014	Liver	6/27/2011	10/23/2013	5/16/2015	
03-017	Cholangiocarcinoma	PTEN_L320fs*5	FoundationOne	FoundationOne_236/19 Dec 2012 - Aug 2014	Liver	6/27/2011	10/23/2013	5/16/2015	5
03-017	Cholangiocarcinoma	ERBB3_P558S	FoundationOne	FoundationOne_236/19 Dec 2012 - Aug 2014	Liver	6/27/2011	10/23/2013	5/16/2015	
03-017	Cholangiocarcinoma	PIK3R1_N453del	FoundationOne	FoundationOne_236/19 Dec 2012 - Aug 2014	Liver	6/27/2011	10/23/2013	5/16/2015	
03-017	Cholangiocarcinoma	SPEN_A1779T	FoundationOne	FoundationOne_236/19 Dec 2012 - Aug 2014	Liver	6/27/2011	10/23/2013	5/16/2015	
03-017	Cholangiocarcinoma	TSC2_R1159W	FoundationOne	FoundationOne_236/19 Dec 2012 - Aug 2014	Liver	6/27/2011	10/23/2013	5/16/2015	
03-017	Cholangiocarcinoma	TSC2_A678T	FoundationOne	FoundationOne_236/19 Dec 2012 - Aug 2014	Liver	6/27/2011	10/23/2013	5/16/2015	
03-017	Cholangiocarcinoma	BCORL1_T1111M	FoundationOne	FoundationOne_236/19 Dec 2012 - Aug 2014	Liver	6/27/2011	10/23/2013	5/16/2015	
03-017	Cholangiocarcinoma	FANCE_R92W	FoundationOne	FoundationOne_236/19 Dec 2012 - Aug 2014	Liver	6/27/2011	10/23/2013	5/16/2015	
03-017	Cholangiocarcinoma	STAT4_I387T	FoundationOne	FoundationOne_236/19 Dec 2012 - Aug 2014	Liver	6/27/2011	10/23/2013	5/16/2015	
03-017	Cholangiocarcinoma	WISP3_I227L	FoundationOne	FoundationOne_236/19 Dec 2012 - Aug 2014	Liver	6/27/2011	10/23/2013	5/16/2015	
03-021	Neuroendocrine carcinoma of pancreas	PTEN loss	Caris	IHC	Liver	12/23/2008	1/13/2014	6/27/2015	6
03-028	Cervix squamous cell carcinoma	PTEN loss	MD Anderson	IHC	Tumor	2/13/2015	3/15/2015	8/25/2015	0
03-028	Cervix squamous cell carcinoma	JAK2_H303fs*4	FoundationOne	FoundationOne_315/28 Aug 2014 - Dec 2017	Peritoneum	8/25/2015	9/23/2015	8/25/2015	
03-028	Cervix squamous cell carcinoma	TSC1_N762S	FoundationOne	FoundationOne_315/28 Aug 2014 - Dec 2017	Peritoneum	8/25/2015	9/23/2015	8/25/2015	
03-028	Cervix squamous cell carcinoma	JAK2_H303fs*4	FoundationOne	FoundationOne_315/28 Aug 2014 - Dec 2017	Peritoneum	8/25/2015	9/23/2015	8/25/2015	
03-028	Cervix squamous cell carcinoma	NOTCH1_C440fs*69	FoundationOne	FoundationOne_315/28 Aug 2014 - Dec 2017	Peritoneum	8/25/2015	9/23/2015	8/25/2015	
03-028	Cervix squamous cell carcinoma	PIK3CA_E545K	FoundationOne	FoundationOne_315/28 Aug 2014 - Dec 2017	Peritoneum	8/25/2015	9/23/2015	8/25/2015	
03-028	Cervix squamous cell carcinoma	PTEN_Q245*	FoundationOne	FoundationOne_315/28 Aug 2014 - Dec 2017	Peritoneum	8/25/2015	9/23/2015	8/25/2015	
03-028	Cervix squamous cell carcinoma	CREBBP_Q887*	FoundationOne	FoundationOne_315/28 Aug 2014 - Dec 2017	Peritoneum	8/25/2015	9/23/2015	8/25/2015	
03-028	Cervix squamous cell carcinoma	MLL2_P1005S	FoundationOne	FoundationOne_315/28 Aug 2014 - Dec 2017	Peritoneum	8/25/2015	9/23/2015	8/25/2015	
03-028	Cervix squamous cell carcinoma	MLL2_Q928*	FoundationOne	FoundationOne_315/28 Aug 2014 - Dec 2017	Peritoneum	8/25/2015	9/23/2015	8/25/2015	
03-028	Cervix squamous cell carcinoma	TSC1_N762S	FoundationOne	FoundationOne_315/28 Aug 2014 - Dec 2017	Peritoneum	8/25/2015	9/23/2015	8/25/2015	
03-028	Cervix squamous cell carcinoma	IRS2_G879S	FoundationOne	FoundationOne_315/28 Aug 2014 - Dec 2017	Peritoneum	8/25/2015	9/23/2015	8/25/2015	
03-028	Cervix squamous cell carcinoma	IRS2_G882A	FoundationOne	FoundationOne_315/28 Aug 2014 - Dec 2017	Peritoneum	8/25/2015	9/23/2015	8/25/2015	
03-028	Cervix squamous cell carcinoma	MDM4_C460fs*5	FoundationOne	FoundationOne_315/28 Aug 2014 - Dec 2017	Peritoneum	8/25/2015	9/23/2015	8/25/2015	
03-028	Cervix squamous cell carcinoma	GLI1_G672E	FoundationOne	FoundationOne_315/28 Aug 2014 - Dec 2017	Peritoneum	8/25/2015	9/23/2015	8/25/2015	
03-028	Cervix squamous cell carcinoma	TGFBR2_Deletion	FoundationOne	FoundationOne_315/28 Aug 2014 - Dec 2017	Peritoneum	8/25/2015	9/23/2015	8/25/2015	
03-030	Mixed high grade serous and clear cell ovarian	PTEN	Caris	IHC	Ovary	9/5/2014	6/12/2015	10/20/2015	3
03-030	Mixed high grade serous and clear cell ovarian	TP53_Q104*	MI Profile	Hotspot Panel, Feb 2013- Jun 2017	Ovary	9/5/2014	6/12/2015	10/20/2015	
03-031	Pleomorphic liposarcoma	TP53_R273H	FoundationOne Heme	FoundationOne Heme_406/31/265	Soft tissue	1/5/2015	7/21/2015	11/4/2015	
03-031	Pleomorphic liposarcoma	NF1_I679fs*21	FoundationOne Heme	FoundationOne Heme_406/31/265	Soft tissue	1/5/2015	7/21/2015	11/4/2015	
03-031	Pleomorphic liposarcoma	PTEN_K267fs*9	FoundationOne Heme	FoundationOne Heme_406/31/265	Soft tissue	1/5/2015	7/21/2015	11/4/2015	3
03-031	Pleomorphic liposarcoma	PTEN_R233*	FoundationOne Heme	FoundationOne Heme_406/31/265	Soft tissue	1/5/2015	7/21/2015	11/4/2015	
03-031	Pleomorphic liposarcoma	MLH1_deletion	FoundationOne Heme	FoundationOne Heme_406/31/265	Soft tissue	1/5/2015	7/21/2015	11/4/2015	
03-031	Pleomorphic liposarcoma	CIC_c1597fs*13+	FoundationOne Heme	FoundationOne Heme_406/31/265	Soft tissue	1/5/2015	7/21/2015	11/4/2015	
03-031	Pleomorphic liposarcoma	MAP3K6_c480+1G>A	FoundationOne Heme	FoundationOne Heme_406/31/265	Soft tissue	1/5/2015	7/21/2015	11/4/2015	
03-031	Pleomorphic liposarcoma	RB1_c1333-2A>T	FoundationOne Heme	FoundationOne Heme_406/31/265	Soft tissue	1/5/2015	7/21/2015	11/4/2015	
03-031	Pleomorphic liposarcoma	DDR2_A727V	FoundationOne Heme	FoundationOne Heme_406/31/265	Soft tissue	1/5/2015	7/21/2015	11/4/2015	
03-031	Pleomorphic liposarcoma	MSH3_L911W	FoundationOne Heme	FoundationOne Heme_406/31/265	Soft tissue	1/5/2015	7/21/2015	11/4/2015	
03-031	Pleomorphic liposarcoma	MYO18A_R1059C	FoundationOne Heme	FoundationOne Heme_406/31/265	Soft tissue	1/5/2015	7/21/2015	11/4/2015	
03-031	Pleomorphic liposarcoma	NFE2L2_c1457fs*12	FoundationOne Heme	FoundationOne Heme_406/31/265	Soft tissue	1/5/2015	7/21/2015	11/4/2015	
03-031	Pleomorphic liposarcoma	PRKDC_I132T	FoundationOne Heme	FoundationOne Heme_406/31/265	Soft tissue	1/5/2015	7/21/2015	11/4/2015	
03-031	Pleomorphic liposarcoma	U2AF2_c231-2_231-1insC	FoundationOne Heme	FoundationOne Heme_406/31/265	Soft tissue	1/5/2015	7/21/2015	11/4/2015	
03-036	Uterine leiomyosarcoma	CDKN2A_Deletion	FoundationOne	FoundationOne_236/19 Dec 2012 - Aug 2014	Lung	5/1/2009	9/30/2013	12/7/2015	
03-036	Uterine leiomyosarcoma	PTEN_Deletion	FoundationOne	FoundationOne_236/19 Dec 2012 - Aug 2014	Lung	5/1/2009	9/30/2013	12/7/2015	4
03-036	Uterine leiomyosarcoma	ARID1A_Deletion	FoundationOne	FoundationOne_236/19 Dec 2012 - Aug 2014	Lung	5/1/2009	9/30/2013	12/7/2015	
03-036	Uterine leiomyosarcoma	CDKN2B_Deletion	FoundationOne	FoundationOne_236/19 Dec 2012 - Aug 2014	Lung	5/1/2009	9/30/2013	12/7/2015	
03-036	Uterine leiomyosarcoma	CDKN2C_Deletion	FoundationOne	FoundationOne_236/19 Dec 2012 - Aug 2014	Lung	5/1/2009	9/30/2013	12/7/2015	
03-036	Uterine leiomyosarcoma	CIC_Deletion	FoundationOne	FoundationOne_236/19 Dec 2012 - Aug 2014	Lung	5/1/2009	9/30/2013	12/7/2015	
03-036	Uterine leiomyosarcoma	LRP1B_E3013*	FoundationOne	FoundationOne_236/19 Dec 2012 - Aug 2014	Lung	5/1/2009	9/30/2013	12/7/2015	
03-036	Uterine leiomyosarcoma	MLL_K2818R	FoundationOne	FoundationOne_236/19 Dec 2012 - Aug 2014	Lung	5/1/2009	9/30/2013	12/7/2015	
04-018	Renal cell carcinoma, clear cell type	BRCA1_IVS13+1G>A	Myriad	Specific Site Analysis	Blood	8/30/2012	9/18/2012	5/212015	3
04-019	colon adenocarcinoma (crc)	BRCA2_c.3185insG	Myriad	BRACAnalysis	Blood	12/5/2005	10/10/2012	6/17/2015	2
04-020	Urothelial carcinoma	PIK3CA_Amplification	FoundationOne	FoundationOne_315/28 Aug 2014 - Dec 2017	Head and Neck, L supraclavicular	1/8/2015	2/25/2015	6/6/2015	
04-020	Urothelial carcinoma	SOX2_Amplification	FoundationOne	FoundationOne_315/28 Aug 2014 - Dec 2017	Head and Neck, L supraclavicular	1/8/2015	2/25/2015	6/6/2015	
04-020	Urothelial carcinoma	PRKCI_Amplification	FoundationOne	FoundationOne_315/28 Aug 2014 - Dec 2017	Head and Neck, L supraclavicular	1/8/2015	2/25/2015	6/6/2015	
04-020	Urothelial carcinoma	TERC_Amplification	FoundationOne	FoundationOne_315/28 Aug 2014 - Dec 2017	Head and Neck, L supraclavicular	1/8/2015	2/25/2015	6/6/2015	
04-020	Urothelial carcinoma	CDKN2A_c.423_457+40del75	FoundationOne	FoundationOne_315/28 Aug 2014 - Dec 2017	Head and Neck, L supraclavicular	1/8/2015	2/25/2015	6/6/2015	
04-020	Urothelial carcinoma	FGFR3_G370C	FoundationOne	FoundationOne_315/28 Aug 2014 - Dec 2017	Head and Neck, L supraclavicular	1/8/2015	2/25/2015	6/6/2015	
04-020	Urothelial carcinoma	TP53_C176Y	FoundationOne	FoundationOne_315/28 Aug 2014 - Dec 2017	Head and Neck, L supraclavicular	1/8/2015	2/25/2015	6/6/2015	
04-020	Urothelial carcinoma	BRCA2_I1364fs*3	FoundationOne	FoundationOne_315/28 Aug 2014 - Dec 2017	Head and Neck, L supraclavicular	1/8/2015	2/25/2015	6/6/2015	
04-020	Urothelial carcinoma	MLL2_L1645fs*77	FoundationOne	FoundationOne_315/28 Aug 2014 - Dec 2017	Head and Neck, L supraclavicular	1/8/2015	2/25/2015	6/6/2015	
04-020	Urothelial carcinoma	BRCA2_c.4320delAT	Myriad	BRACAnalysis	Blood	unknown	Unknown	6/6/2015	1
04-024	Pancreatic cancer	BRCA2_c.6718delC	Myriad	BRACAnalysis	Blood	4/7/2003	4/25/2003	7/27/2015	1
04-026	Urothelial carcinoma	KIT_M541L	CMS50	CMS50_01042017	Right lateral bladder tumor	8/22/2013	12/7/2013	8/13/2015	
04-026	Urothelial carcinoma	PIK3CA_N1044K	CMS50	CMS50_01042017	Right lateral bladder tumor	8/22/2013	12/7/2013	8/13/2015	
04-026	Urothelial carcinoma	BRCA1_c.5386insC	Myriad	BRACAnalysis	Blood	11/15/2007	12/3/2007	8/13/2015	5
04-027	Esophageal squamous cell carcinoma	BRCA1_Y1463X (4508C>A)	Myriad	BRACAnalysis	Blood	5/14/2012	5/21/2012	8/7/2015	1
04-029	Ampullary adenocarcinoma	BRCA2_c.8633-1G>C	Myriad	BRACAnalysis	Blood	3/19/2015	3/30/2015	9/14/2015	0
04-029	Ampullary adenocarcinoma	EGFR_G735R	Guardant360	Guardant360_73Gene Nov2016 - Mar2019	Blood	2/6/2018	2/16/2018	9/14/2015	
04-029	Ampullary adenocarcinoma	EGFR_P373L	Guardant360	Guardant360_73Gene Nov2016 - Mar2019	Blood	2/6/2018	2/16/2018	9/14/2015	
04-029	Ampullary adenocarcinoma	PDGFRA_I1060del	Guardant360	Guardant360_73Gene Nov2016 - Mar2019	Blood	2/6/2018	2/16/2018	9/14/2015	
04-029	Ampullary adenocarcinoma	SMAD4_Q366*	Guardant360	Guardant360_73Gene Nov2016 - Mar2019	Blood	2/6/2018	2/16/2018	9/14/2015	
04-029	Ampullary adenocarcinoma	TP53_V173L	Guardant360	Guardant360_73Gene Nov2016 - Mar2019	Blood	2/6/2018	2/16/2018	9/14/2015	
04-029	Ampullary adenocarcinoma	BRCA2_T3033fs*11	Guardant360	Guardant360_73Gene Nov2016 - Mar2019	Blood	2/6/2018	2/16/2018	9/14/2015	
04-029	Ampullary adenocarcinoma	SMAD4_Q366*	FoundationOne	FoundationOne_315/28 Aug 2014 - Dec 2017	Ampulla of Vater	8/26/2013	3/30/2018	9/14/2015	
04-029	Ampullary adenocarcinoma	TP53_V173L	FoundationOne	FoundationOne_315/28 Aug 2014 - Dec 2017	Ampulla of Vater	8/26/2013	3/30/2018	9/14/2015	
04-029	Ampullary adenocarcinoma	BRCA2_c.8633-1G>C	FoundationOne	FoundationOne_315/28 Aug 2014 - Dec 2017	Ampulla of Vater	8/26/2013	3/30/2018	9/14/2015	
04-029	Ampullary adenocarcinoma	CHEK1_A306V	FoundationOne	FoundationOne_315/28 Aug 2014 - Dec 2017	Ampulla of Vater	8/26/2013	3/30/2018	9/14/2015	
04-029	Ampullary adenocarcinoma	CREBBP_A1907T	FoundationOne	FoundationOne_315/28 Aug 2014 - Dec 2017	Ampulla of Vater	8/26/2013	3/30/2018	9/14/2015	
04-029	Ampullary adenocarcinoma	WT1_S20C	FoundationOne	FoundationOne_315/28 Aug 2014 - Dec 2017	Ampulla of Vater	8/26/2013	3/30/2018	9/14/2015	
04-029	Ampullary adenocarcinoma	STAT4_G413A	FoundationOne	FoundationOne_315/28 Aug 2014 - Dec 2017	Ampulla of Vater	8/26/2013	3/30/2018	9/14/2015	
04-029	Ampullary adenocarcinoma	PLCG2_W578*	FoundationOne	FoundationOne_315/28 Aug 2014 - Dec 2017	Ampulla of Vater	8/26/2013	3/30/2018	9/14/2015	
04-029	Ampullary adenocarcinoma	QKI_P246L	FoundationOne	FoundationOne_315/28 Aug 2014 - Dec 2017	Ampulla of Vater	8/26/2013	3/30/2018	9/14/2015	
04-034	Cholangiocarcinoma	BRCA2_2041insA	Other	Single site Analysis	Blood	1/6/2011	Unknown	11/11/2015	5
04-037	Parapharyngeal solitary fibrous tumor	BRCA1_K679*	FoundationOne Heme	FoundationOne Heme_406/31/265	Soft Tissue	2/16/2001	11/2/2015	12/28/2015	
04-037	Parapharyngeal solitary fibrous tumor	ASMTL_G375S	FoundationOne Heme	FoundationOne Heme_406/31/265	Soft Tissue	2/16/2001	11/2/2015	12/28/2015	
04-037	Parapharyngeal solitary fibrous tumor	BCORL1_R890Q	FoundationOne Heme	FoundationOne Heme_406/31/265	Soft Tissue	2/16/2001	11/2/2015	12/28/2015	
04-037	Parapharyngeal solitary fibrous tumor	BRCA1_L246V	FoundationOne Heme	FoundationOne Heme_406/31/265	Soft Tissue	2/16/2001	11/2/2015	12/28/2015	
04-037	Parapharyngeal solitary fibrous tumor	BRIP1_H396R	FoundationOne Heme	FoundationOne Heme_406/31/265	Soft Tissue	2/16/2001	11/2/2015	12/28/2015	
04-037	Parapharyngeal solitary fibrous tumor	NCOR2_V564M	FoundationOne Heme	FoundationOne Heme_406/31/265	Soft Tissue	2/16/2001	11/2/2015	12/28/2015	
04-037	Parapharyngeal solitary fibrous tumor	NOTCH2_R91L	FoundationOne Heme	FoundationOne Heme_406/31/265	Soft Tissue	2/16/2001	11/2/2015	12/28/2015	
04-037	Parapharyngeal solitary fibrous tumor	TGFBR2_P26S	FoundationOne Heme	FoundationOne Heme_406/31/265	Soft Tissue	2/16/2001	11/2/2015	12/28/2015	
04-037	Parapharyngeal solitary fibrous tumor	TNFAIP3_R280Q	FoundationOne Heme	FoundationOne Heme_406/31/265	Soft Tissue	2/16/2001	11/2/2015	12/28/2015	
04-037	Parapharyngeal solitary fibrous tumor	BRCA1_K679*	Myriad	BRACAnalysis	Blood	5/7/2008	5/15/2008	12/28/2015	4
04-037	Parapharyngeal solitary fibrous tumor	STAT6-NAB2	FoundationOne Heme	FoundationOne Heme_406/31/265	Soft Tissue	2/16/2001	11/2/2015	12/28/2015	
04-052	Adenocarcinoma of cecum	BRCA2_K944*	AmbryGenetics	CustomNext-Cancer	Blood	12/5/2016	12/22/2016	8/10/2018	1
04-052	Adenocarcinoma of cecum	MSH6_H1203Y	AmbryGenetics	CustomNext-Cancer	Blood	12/5/2016	12/22/2016	8/10/2018	
04-053	Sebaceous adenocarcinoma	BRCA1_c.754delC	Ambry genetic	Specific Site Analysis	Blood	5/16/2017	5/30/2017	7/20/2018	2
04-053	Sebaceous adenocarcinoma	TP53_Q331*	STGAv1	STGAv1_03132018	Left superficial parotid	4/15/2015	4/28/2017	7/20/2018	
04-065	Gastric adenocarcinoma	BRAC2_Trp2628	GeneDx	Custom Panel 1053889	Blood	1/8/2016	2/1/2016	11/20/2018	3
04-065	Gastric adenocarcinoma	TP53_C275fs*70	CMS50	CMS50_01042017	unknown	11/25/2015	12/2/2015	11/20/2018	
04-065	Gastric adenocarcinoma	PTEN_Q298*	CMS50	CMS50_01042017	unknown	11/25/2015	12/2/2015	11/20/2018	
04-075	Pancreas adenocarcinoma	BRCA2_D252Vfs*24	GeneDx	BRCA 1/2 sequencing and Del/Dup Analysis	Blood	1/30/2016	2/10/2016	5/14/2019	3

04-075	Pancreas adenocarcinoma	NBN_W2*	STGA-DNA 2018	STGA-DNA 2018_11012021	Soft Tissue	1/6/2020	1/27/2020	5/14/2019	
04-075	Pancreas adenocarcinoma	TP53_R248Q	STGA-DNA 2018	STGA-DNA 2018_11012021	Soft Tissue	1/6/2020	1/27/2020	5/14/2019	
04-075	Pancreas adenocarcinoma	BRCA2_N257fs*16	STGA-DNA 2018	STGA-DNA 2018_11012021	Soft Tissue	1/6/2020	1/27/2020	5/14/2019	
04-075	Pancreas adenocarcinoma	KRAS_G12D	STGA-DNA 2018	STGA-DNA 2018_11012021	Soft Tissue	1/6/2020	1/27/2020	5/14/2019	
04-075	Pancreas adenocarcinoma	KRAS_G12D	Liquid Biopsy Panel V1	Liquid Biopsy Panel V1_11072021	Plasma circulating cell-free DNA	3/26/2020	4/3/2020	5/14/2019	
04-075	Pancreas adenocarcinoma	TP53_Y220C	Liquid Biopsy Panel V1	Liquid Biopsy Panel V1_11072021	Plasma circulating cell-free DNA	3/26/2020	4/3/2020	5/14/2019	
04-075	Pancreas adenocarcinoma	BRCA2_N257fs	Liquid Biopsy Panel V1	Liquid Biopsy Panel V1_11072021	Plasma circulating cell-free DNA	3/26/2020	4/3/2020	5/14/2019	
04-075	Pancreas adenocarcinoma	TP53_R248Q	Liquid Biopsy Panel V1	Liquid Biopsy Panel V1_11072021	Plasma circulating cell-free DNA	3/26/2020	4/3/2020	5/14/2019	
04-076	Pancreas adenocarcinoma	BRCA1_943ins10	Myriad	Comprehensive BRACAnalysis	Blood	11/12/2008	11/20/2008	6/21/2019	5
04-076	Pancreas adenocarcinoma	KRAS_G12V	Liquid Biopsy Panel V1	Liquid Biopsy Panel V1_11072021	Plasma circulating cell-free DNA	1/14/2019	1/24/2019	6/21/2019	
04-076	Pancreas adenocarcinoma	TP53_R306*	Liquid Biopsy Panel V1	Liquid Biopsy Panel V1_11072021	Plasma circulating cell-free DNA	1/14/2019	1/24/2019	6/21/2019	
04-076	Pancreas adenocarcinoma	AR_I2K	Liquid Biopsy Panel V1	Liquid Biopsy Panel V1_11072021	Plasma circulating cell-free DNA	1/14/2019	1/24/2019	6/21/2019	
04-084	Neuroendocrine carcinoma of pancreas	BRCA1_Q563*	Invitae	Sequence analysis and Del/Dup Analysis	Blood	4/24/2019	5/2/2019	2/4/2020	1
04-087	Anus squamous cell carcinoma,	BRCA1_C61G	Invitae	Sequence analysis and Del/Dup Analysis	Blood	10/11/2019	10/22/2019	9/17/2020	0
04-087	Anus squamous cell carcinoma,	BRCA1_C61G	Tempus xT Assay	Tempus XT 596 Gene List	Pelvis	8/9/2019	9/11/2019	9/17/2020	
04-087	Anus squamous cell carcinoma,	FBXW7_R479G	Tempus xT Assay	Tempus XT 596 Gene List	Pelvis	8/9/2019	9/11/2019	9/17/2020	
04-087	Anus squamous cell carcinoma,	KMT2C_R2139*	Tempus xT Assay	Tempus XT 596 Gene List	Pelvis	8/9/2019	9/11/2019	9/17/2020	
04-087	Anus squamous cell carcinoma,	ERBB2_R678Q	Tempus xT Assay	Tempus XT 596 Gene List	Pelvis	8/9/2019	9/11/2019	9/17/2020	
04-087	Anus squamous cell carcinoma,	FAT1_Y400fs*1	Tempus xT Assay	Tempus XT 596 Gene List	Pelvis	8/9/2019	9/11/2019	9/17/2020	
04-087	Anus squamous cell carcinoma,	KMT2D_Q4007*	Tempus xT Assay	Tempus XT 596 Gene List	Pelvis	8/9/2019	9/11/2019	9/17/2020	
04-087	Anus squamous cell carcinoma,	DNM2_R361*	Tempus xT Assay	Tempus XT 596 Gene List	Pelvis	8/9/2019	9/11/2019	9/17/2020	
04-087	Anus squamous cell carcinoma,	NFE2L2_R34Q	Tempus xT Assay	Tempus XT 596 Gene List	Pelvis	8/9/2019	9/11/2019	9/17/2020	
04-087	Anus squamous cell carcinoma,	BCOR_E1199K	Tempus xT Assay	Tempus XT 596 Gene List	Pelvis	8/9/2019	9/11/2019	9/17/2020	
04-087	Anus squamous cell carcinoma,	ERBB4_E64Q	Tempus xT Assay	Tempus XT 596 Gene List	Pelvis	8/9/2019	9/11/2019	9/17/2020	
04-087	Anus squamous cell carcinoma,	EP300_Y1446C	Tempus xT Assay	Tempus XT 596 Gene List	Pelvis	8/9/2019	9/11/2019	9/17/2020	
04-087	Anus squamous cell carcinoma,	ALK_G821R	Tempus xT Assay	Tempus XT 596 Gene List	Pelvis	8/9/2019	9/11/2019	9/17/2020	
04-087	Anus squamous cell carcinoma,	ETV1_D162H	Tempus xT Assay	Tempus XT 596 Gene List	Pelvis	8/9/2019	9/11/2019	9/17/2020	
04-087	Anus squamous cell carcinoma,	IFNGR2_Q34*	Tempus xT Assay	Tempus XT 596 Gene List	Pelvis	8/9/2019	9/11/2019	9/17/2020	
04-087	Anus squamous cell carcinoma,	CTNNA1_S558*	Tempus xT Assay	Tempus XT 596 Gene List	Pelvis	8/9/2019	9/11/2019	9/17/2020	
04-087	Anus squamous cell carcinoma,	RXRA_A241fs*18	Tempus xT Assay	Tempus XT 596 Gene List	Pelvis	8/9/2019	9/11/2019	9/17/2020	
04-087	Anus squamous cell carcinoma,	LDLR_L790P	Tempus xT Assay	Tempus XT 596 Gene List	Pelvis	8/9/2019	9/11/2019	9/17/2020	
04-087	Anus squamous cell carcinoma,	SPEN_A3009S	Tempus xT Assay	Tempus XT 596 Gene List	Pelvis	8/9/2019	9/11/2019	9/17/2020	
04-087	Anus squamous cell carcinoma,	ASNS_E227Q	Tempus xT Assay	Tempus XT 596 Gene List	Pelvis	8/9/2019	9/11/2019	9/17/2020	
04-087	Anus squamous cell carcinoma,	KMT2B_E789K	Tempus xT Assay	Tempus XT 596 Gene List	Pelvis	8/9/2019	9/11/2019	9/17/2020	
04-087	Anus squamous cell carcinoma,	CEBPA_H195_P196dup	LMPv1	EndLeukemia Mutation Panel V1_11052021	Bone marrow	7/21/2021	7/27/2021	9/17/2020	
04-087	Anus squamous cell carcinoma,	CBLC_P435S	LMPv1	EndLeukemia Mutation Panel V1_11052021	Bone marrow	7/21/2021	7/27/2021	9/17/2020	
04-087	Anus squamous cell carcinoma,	TET2_V218M	LMPv1	EndLeukemia Mutation Panel V1_11052021	Bone marrow	7/21/2021	7/27/2021	9/17/2020	
04-087	Anus squamous cell carcinoma,	TET2_H1778R	LMPv1	EndLeukemia Mutation Panel V1_11052021	Bone marrow	7/21/2021	7/27/2021	9/17/2020	
04-087	Anus squamous cell carcinoma,	TET2_P363L	LMPv1	EndLeukemia Mutation Panel V1_11052021	Bone marrow	7/21/2021	7/27/2021	9/17/2020	
04-087	Anus squamous cell carcinoma,	TET2_L34F	LMPv1	EndLeukemia Mutation Panel V1_11052021	Bone marrow	7/21/2021	7/27/2021	9/17/2020	
04-087	Anus squamous cell carcinoma,	ATR_E24Q	STGA-DNA 2018	STGA-DNA 2018_11012021	Lymph Node	6/23/2021	8/7/2021	9/17/2020	
04-087	Anus squamous cell carcinoma,	ERBB2_R678Q	STGA-DNA 2018	STGA-DNA 2018_11012021	Lymph Node	6/23/2021	8/7/2021	9/17/2020	
04-087	Anus squamous cell carcinoma,	AKT2_Amplification	STGA-DNA 2018	STGA-DNA 2018_11012021	Lymph Node	6/23/2021	8/7/2021	9/17/2020	
04-087	Anus squamous cell carcinoma,	CCNE1_Amplification	STGA-DNA 2018	STGA-DNA 2018_11012021	Lymph Node	6/23/2021	8/7/2021	9/17/2020	

@ lines of systematic treatment administered subsequent to molecular testing for study enrollment and preceding the initiation of talazoparib treatment

Supplementary Note 1: WES_mutation and CNA List

Patient_ID	gene	exonicfunc	key	transcript
03-028	PTEN	stop_gained	10_89717708_C/T	ENST00000371953
03-028	CDK12	non_synonymous_	17_37657600_C/G	ENST00000430627
03-030	PTEN	non_synonymous_	10_89692868_C/G	ENST00000371953
03-031	PTEN	frameshift_variant	10_89717770_A/-	ENST00000371953
03-013	PTEN	stop_gained	10_89720852_C/T	ENST00000371953
03-001	PTEN	frameshift_variant	10_89624242_AA/-	ENST00000371953
03-001	PTEN	frameshift_variant	10_89720799_TAC	ENST00000371953
03-004	PTEN	frameshift_variant	10_89717735_AAA	ENST00000371953
03-004	ATM	non_synonymous_	11_108153535_A/(	ENST00000278616
03-004	ARID1A	non_synonymous_	1_27106727_C/T	ENST00000324856
03-017	PTEN	frameshift_variant	10_89720806_-/T	ENST00000371953
03-021	PTEN	stop_gained	10_89624269_A/T	ENST00000371953
03-021	ATM	non_synonymous_	11_108124557_G/	ENST00000278616
03-021	ATM	splice_donor_varia	11_108168110_G/	ENST00000278616
03-021	ATM	non_synonymous_	11_108186770_G/	ENST00000278616
03-021	ARID1A	non_synonymous_	1_27106558_C/T	ENST00000324856
03-021	BRCA2	non_synonymous_	13_32972723_G/A	ENST00000544455
03-021	PALB2	non_synonymous_	16_23647307_C/T	ENST00000261584
03-021	PALB2	non_synonymous_	16_23652462_G/A	ENST00000261584
03-021	CDK12	non_synonymous_	17_37627934_G/A	ENST00000430627
03-021	BRCA1	non_synonymous_	17_41223085_G/A	ENST00000412061
03-021	BRIP1	non_synonymous_	17_59861743_C/T	ENST00000259008
03-021	BRIP1	stop_gained	17_59934578_G/T	ENST00000259008
03-016	BARD1	non_synonymous_	2_215617178_G/C	ENST00000260947

cdna	aaannotation	AlleleFreq	dbSNP	Mutation_Type
ENST00000371953.3	PTEN_Q245*	34.64	NA	Somatic
ENST00000430627.1	CDK12_F839L	22.79	NA	Somatic
ENST00000371953.3	PTEN_H118D	40.28	NA	Somatic
ENST00000371953.3	-	11.64	NA	Somatic
ENST00000371953.3	PTEN_R335*	50	rs121909231	Somatic
ENST00000371953.3	-	20.18	NA	Somatic
ENST00000371953.3	-	8.7	NA	Somatic
ENST00000371953.3	-	68.79	NA	Somatic
ENST00000278616.4	ATM_Q1225H	34.95	NA	Somatic
ENST00000324856.7	ARID1A_S2113F	20.93	NA	Somatic
ENST00000371953.3	-	13.95	NA	Somatic
ENST00000371953.3	PTEN_R15*	70.29	NA	Somatic
ENST00000278616.4	ATM_D639N	15.58	NA	Somatic
ENST00000278616.4	-	36.76	NA	Somatic
ENST00000278616.4	ATM_G2043D	31.25	NA	Somatic
ENST00000324856.7	ARID1A_R2057W	24.04	NA	Somatic
ENST00000544455.1	BRCA2_G3358E	19.3	NA	Somatic
ENST00000261584.4	PALB2_P187L	38.67	NA	Somatic
ENST00000261584.4	PALB2_G6E	21.47	NA	Somatic
ENST00000430627.1	CDK12_A617T	10.6	NA	Somatic
ENST00000412061.2	BRCA1_A1637T	18.79	NA	Somatic
ENST00000259008.1	BRIP1_P506S	15.65	NA	Somatic
ENST00000259008.1	BRIP1_E74*	33.19	NA	Somatic
ENST00000260947.4	BARD1_C557S	0.4296	rs28997576	Germline

Patient _ID	gene	CN	CNS
03-005	PTEN	0.9	DEL
03-010	PTEN	0.8	DEL

Supplementary Note 2: R code used for PTEN cohort analysis.

```
require(pacman)
p_load(tidyverse, data.table, ggplot2, readxl, ComplexHeatmap, RColorBrewer, grid)


########### Read the data #################
ci <- readRDS("patient_information_cohort3.rds")
alt.mtx.clia <- readRDS("Clinical_alterations_cohort3.rds")
wes.germline <- readRDS("WES_germline_mutation_cohort3.rds")
wes.somatic <- readRDS("WES_somatic_mutation_cohort3.rds")
wes.cna <- readRDS("WES_copyNumberAlteration_cohort3.rds")

genes <- c('BRCA1', 'BRCA2', 'ATM', 'BRIP1', 'BARD1', 'CDK12', 'CHEK1', 'CHEK2', 'PALB2',
           'RAD51D', 'ARID1A', 'ATR', "PTEN", "RAD51", "EMSY", "FANCC")


setDT(wes.germline)
setDT(wes.somatic)
setDT(wes.cna)


altList.wes <- rbind(wes.somatic[, .(Patient_ID, gene, alteration="Somatic_MUT")],
                     wes.germline[, .(Patient_ID, gene, alteration="Germline_MUT")],
                     wes.cna[!grepl(CNS, pattern=","), .(Patient_ID, gene, alteration=CNS)])

alt.mtx.wes <- altList.wes %>%
  rbind(., tibble(Patient_ID = setdiff(ci$Patient_ID, altList.wes$Patient_ID),
                  gene = setdiff(genes, altList.wes$gene),
                  alteration="")) %>% # add the patients without WES alterations and the gene not al
  mutate(MutationType = paste0("WES_", alteration)) %>%
  aggregate(MutationType ~ Patient_ID + gene, data=., FUN = function(x) paste(unique(x), collapse=";
  as.data.table() %>%
  dcast(., gene ~ Patient_ID, value.var = "MutationType", fill="") %>%
  column_to_rownames('gene')

alt.mtx.wes.with_background <-
  matrix(paste0("WES_Mut/Amp/Del;", as.matrix(alt.mtx.wes)),
                                   nrow = nrow(alt.mtx.wes), ncol = ncol(alt.mtx.wes),
                                   dimnames=dimnames(alt.mtx.wes))

############ Merge WES and CLIA alterations ##########
alt.mtx.ddr <- matrix(paste(as.matrix(alt.mtx.clia[genes, ci$Patient_ID]),
                            as.matrix(alt.mtx.wes.with_background[genes, ci$Patient_ID]),
                            sep=';'),
                      nrow=length(genes), ncol=nrow(ci),
                      dimnames = list(genes, ci$Patient_ID))


############ Oncoprint ##########
## define color and functions for heatmap.
cb.col <- structure(brewer.pal(8, name = "Paired")[1:2],
                    names = c("N", "Y"))

brr.col <- structure(brewer.pal(8, name = "Set2")[5:6],
                     names = unique(ci$`Best RECIST response`))


col <- c("CLIA_IHC_Loss" = "black", "CLIA_DEL" = "blue", "CLIA_AMP" = "red",
         "CLIA_Somatic_MUT" = "darkgreen", "CLIA_Germline_MUT" = "darkgoldenrod", "CLIA_Fusion"="dar
         "WES_DEL" = "blue", "WES_AMP" = "red",
         "WES_Somatic_MUT" = "darkgreen", "WES_Germline_MUT" = "darkgoldenrod",
         "CLIA_Not_tested" = "#F0F0F0", "CLIA_Mut" = "#E6F5C9",
         "CLIA_Amp" = "#FFF2AE", "CLIA_Mut/Amp" = "#CBD5E8", "CLIA_Mut/Amp/Del" = "#D9D9D9",
         "WES_Mut/Amp/Del" = "#D9D9D9")

alter_fun = list(
  background = function(x, y, w, h) {
    grid.polygon(
      unit.c(x - 0.5*w, x - 0.5*w, x + 0.5*w),
      unit.c(y - 0.5*h, y + 0.5*h, y - 0.5*h),
      gp = gpar(fill = "white", col = "#969696"))
```

```
  grid.polygon(
    unit.c(x + 0.5*w, x + 0.5*w, x - 0.5*w),
    unit.c(y + 0.5*h, y - 0.5*h, y + 0.5*h),
    gp = gpar(fill = "white", col = "#969696"))
},
CLIA_Not_tested = function(x, y, w, h) {
  grid.polygon(
    unit.c(x - 0.5*w, x - 0.5*w, x + 0.5*w),
    unit.c(y - 0.5*h, y + 0.5*h, y - 0.5*h),
    gp = gpar(fill = col["CLIA_Not_tested"], col = "white"))
},
CLIA_Mut = function(x, y, w, h) {
  grid.polygon(
    unit.c(x - 0.5*w, x - 0.5*w, x + 0.5*w),
    unit.c(y - 0.5*h, y + 0.5*h, y - 0.5*h),
    gp = gpar(fill = col["CLIA_Mut"], col = "white"))
},
CLIA_Amp = function(x, y, w, h) {
  grid.polygon(
    unit.c(x - 0.5*w, x - 0.5*w, x + 0.5*w),
    unit.c(y - 0.5*h, y + 0.5*h, y - 0.5*h),
    gp = gpar(fill = col["CLIA_Amp"], col = "white"))
},
`CLIA_Mut/Amp` = function(x, y, w, h) {
  grid.polygon(
    unit.c(x - 0.5*w, x - 0.5*w, x + 0.5*w),
    unit.c(y - 0.5*h, y + 0.5*h, y - 0.5*h),
    gp = gpar(fill = col["CLIA_Mut/Amp"], col = "white"))
},
`CLIA_Mut/Amp/Del` = function(x, y, w, h) {
  grid.polygon(
    unit.c(x - 0.5*w, x - 0.5*w, x + 0.5*w),
    unit.c(y - 0.5*h, y + 0.5*h, y - 0.5*h),
    gp = gpar(fill = col["CLIA_Mut/Amp/Del"], col = "white"))
},
`WES_Mut/Amp/Del` = function(x, y, w, h) {
  grid.polygon(
    unit.c(x + 0.5*w, x + 0.5*w, x - 0.5*w),
    unit.c(y + 0.5*h, y - 0.5*h, y + 0.5*h),
    gp = gpar(fill = col["WES_Mut/Amp/Del"], col = "white"))
},
`CLIA_Somatic_MUT` = function(x, y, w, h) {
  grid.polygon(
    unit.c(x - 0.5*w, x - 0.5*w, x),
    unit.c(y, y - 0.5*h, y),
    gp = gpar(fill = col["CLIA_Somatic_MUT"], col = col["CLIA_Somatic_MUT"]))
},
`CLIA_AMP` = function(x, y, w, h) {
  grid.polygon(
    unit.c(x - 0.5*w, x, x),
    unit.c(y - 0.5*h, y - 0.5*h, y),
    gp = gpar(fill = col["CLIA_AMP"], col = col["CLIA_AMP"]))
},
`CLIA_DEL` = function(x, y, w, h) {
  grid.polygon(
    unit.c(x - 0.5*w, x, x),
    unit.c(y - 0.5*h, y - 0.5*h, y),
    gp = gpar(fill = col["CLIA_DEL"], col = col["CLIA_DEL"]))
},
`CLIA_Germline_MUT` = function(x, y, w, h) {
  grid.polygon(
    unit.c(x, x, x + 0.5*w),
    unit.c(y, y - 0.5*h, y - 0.5*h),
    gp = gpar(fill = col["CLIA_Germline_MUT"], col = col["CLIA_Germline_MUT"]))
},
`CLIA_IHC_Loss` = function(x, y, w, h) {
  grid.polygon(
    unit.c(x - 0.5*w, x - 0.5*w, x),
    unit.c(y + 0.5*h, y, y),
    gp = gpar(fill = col["CLIA_IHC_Loss"], col = col["CLIA_IHC_Loss"]))
```

```
  },
  `CLIA_Fusion` = function(x, y, w, h) {
    grid.polygon(
      unit.c(x - 0.5*w, x - 0.5*w, x),
      unit.c(y + 0.5*h, y, y),
      gp = gpar(fill = col["CLIA_Fusion"], col = col["CLIA_Fusion"]))
  },
  `WES_Somatic_MUT` = function(x, y, w, h) {
    grid.polygon(
      unit.c(x, x+0.5*w, x+0.5*w),
      unit.c(y, y, y+0.5*h),
      gp = gpar(fill = col["WES_Somatic_MUT"], col = col["WES_Somatic_MUT"]))
  },
  `WES_AMP` = function(x, y, w, h) {
    grid.polygon(
      unit.c(x, x, x+0.5*w),
      unit.c(y+0.5*h, y, y+0.5*h),
      gp = gpar(fill = col["WES_AMP"], col = col["WES_AMP"]))
  },
  `WES_DEL` = function(x, y, w, h) {
    grid.polygon(
      unit.c(x, x, x+0.5*w),
      unit.c(y+0.5*h, y, y+0.5*h),
      gp = gpar(fill = col["WES_DEL"], col = col["WES_DEL"]))
  },
  `WES_Germline_MUT` = function(x, y, w, h) {
    grid.polygon(
      unit.c(x-0.5*w, x, x),
      unit.c(y+0.5*h, y, y+0.5*h),
      gp = gpar(fill = col["WES_Germline_MUT"], col = col["WES_Germline_MUT"]))
  }
)


ha <- HeatmapAnnotation(`best tumor size change from baseline (%)` =
                          anno_barplot(ci$Best_recist_response,
                                       ylim = c(-110, 110),
                                       height = unit(4, "cm"),
                                       border = F,
                                       gp = gpar(fill = cohort.col[3]),
                                       axis_param = list(at = c(-100, -30, 0, 20, 100))),
                        `Best overall response` = anno_simple(ci$`Best RECIST response`,
                                                              col=brr.col,
                                                              gp=gpar(col="white", lwd=2),
                                                              height = unit(5, 'mm')),
                        `Clinical benefit` = anno_simple(ci$`Clinical benefit`,
                                                         col=cb.col,
                                                         gp=gpar(col="white", lwd=2),
                                                         height = unit(5, 'mm')),
                        annotation_name_rot=c(90, 0, 0),
                        annotation_name_side = "left",
                        annotation_name_gp = gpar(fontsize=6))


lgd1 <- Legend(title = "Source of alteration",
               labels = c('CLIA', 'WES'),
               labels_gp = gpar(fontsize = 7),
               title_gp = gpar(fontsize=8, fontface="bold"),
               graphics = list(
                 function(x, y, w, h) {
                   grid.polygon(
                     unit.c(x - 0.4*w, x - 0.4*w, x + 0.4*w),
                     unit.c(y - 0.4*h, y + 0.4*h, y - 0.4*h),
                     gp = gpar(fill = "white", col = "grey"))
                 },
                 function(x, y, w, h) {
                   grid.polygon(
                     unit.c(x + 0.4*w, x + 0.4*w, x - 0.4*w),
                     unit.c(y + 0.4*h, y - 0.4*h, y + 0.4*h),
                     gp = gpar(fill = "white", col = "grey"))
```

```
              }
            ))

lgd2 <- Legend(title = "Alteration type assessed",
               labels = c('None', 'Mut', 'Mut/Amp', 'Mut/Amp/Del'),
               legend_gp = gpar(fill = col[c(11, 12, 14, 15)]),
               labels_gp = gpar(fontsize = 7),
               title_gp = gpar(fontsize=6, fontface="bold"))


lgd3 <- Legend(title = "Genomic alteration",
               labels = c('IHC loss', 'Deletion', 'Amplification', 'Somatic mutation', 'Germline mut
               legend_gp = gpar(fill = col[c('CLIA_IHC_Loss', 'CLIA_DEL', 'CLIA_AMP', 'CLIA_Somatic_
                                             'CLIA_Germline_MUT')]),
               labels_gp = gpar(fontsize = 7),
               title_gp = gpar(fontsize=8, fontface="bold"))

lgd4 <- Legend(title = "Best overall response", labels = names(brr.col)[1:2],
               legend_gp = gpar(fill = brr.col[1:2]),
               labels_gp = gpar(fontsize = 7),
               title_gp = gpar(fontsize=7, fontface="bold"))

lgd5 <- Legend(title = "Clinical benefit", labels = names(cb.col),
               legend_gp = gpar(fill = cb.col),
               labels_gp = gpar(fontsize = 7),
               title_gp = gpar(fontsize=7, fontface="bold"))


lgdList <- packLegend(list=list(lgd1, lgd2, lgd3, lgd4, lgd5), direction='vertical')


op <- oncoPrint(alt.mtx.ddr.cohort3,
                alter_fun = alter_fun, col = col,
                top_annotation = ha,
                right_annotation = NULL,
                show_heatmap_legend=F,
                show_column_names=T,
                show_row_names=T,
                show_pct=F,
                column_names_gp=gpar(fontsize=6),
                row_names_gp=gpar(fontsize=7),
                row_title="",
                row_title_side="left",
                column_order = 1:ncol(alt.mtx.ddr.cohort3),
                # heatmap_legend_param = heatmap_legend_param.ddr,
                # column_split = ci$Cohort,
                column_title = "Cohort 3: PTEN Mutation or Loss by IHC",
                column_title_gp = gpar(fontsize=8, fontface="bold"),
                column_gap = unit(3, 'mm'),
                remove_empty_rows=F)


op <- draw(op, heatmap_legend_list = lgdList, heatmap_legend_side = "right")

asterix.index <- which(ci.cohort3$Asterix == "Y")
asterix.y.pos <- ci.cohort3$Best_recist_response[asterix.index] %>%
  ifelse(. >= 0, .+ 5, .)

asterix.x.pos <- asterix.index - 0.08

pdf(file.path(res.dir, "alt_ddr_cohort3.pdf"), width=6)
op
decorate_annotation("best tumor size change from baseline (%)", {
  grid.lines(c(0.45, 12), c(20, 20), gp = gpar(lty = 3, col = "black"),
             default.units = "native")
})
decorate_annotation("best tumor size change from baseline (%)", {
  grid.lines(c(0.45, 12), c(-30, -30), gp = gpar(lty = 3, col = "black"), default.units = "native")

})
```

```
decorate_annotation("best tumor size change from baseline (%)", {
  grid.text("*", x = asterix.x.pos, y = asterix.y.pos,
            default.units = "native", just = c("left", "bottom"),
            gp = gpar(fontsize = 10, col = "black", fontface="bold"), rot = 0)
})
dev.off()
```
