# Supplementary material for: Phase II study of talazoparib in advanced cancers with BRCA1/2, DNA repair, and PTEN alterations
Source: NPJ Precis Oncol. 2024 Jul 31;8:166. doi: 10.1038/s41698-024-00634-6 (PMC11291882; doi:10.1038/s41698-024-00634-6)
Supplement: Supplementary file 4 — Reporting summary [file 41698_2024_634_MOESM4_ESM.pdf]

MD Anderson IND Sponsor Cover Sheet

**Protocol ID**	2013-0961
**Protocol Title**	Phase II Study of the PARP Inhibitor Talazoparib in Advanced Cancer Patients with Somatic Alterations in BRCA1/2, Mutations/Deletions in Other BRCA Pathway Genes and Germline Mutation in BRCA1/2 (not breast or ovarian cancer)
**Phase**	Phase II
**Version**	01 Feb 2024
**Version Date**	02/01/2024
**Protocol PI**	Sarina A. Piha-Paul, MD
**Department**	Investigational Cancer Therapeutics
**Investigational Products**	Talazoparib
**IND Sponsor**	MD Anderson Cancer Center
**IND #**	124,245

TITLE OF STUDY: Phase II Study of the PARP Inhibitor Talazoparib in Advanced Cancer Patients with Somatic Alterations in *BRCA1/2*, Mutations/Deletions in Other *BRCA* Pathway Genes and Germline Mutation in *BRCA1/2* (not breast or ovarian cancer)

Indication: Advanced cancer patients with one of the following:

1) Somatic mutations or deletions of *BRCA1* or *BRCA2*
2) Mutations or homozygous deletions in other *BRCA* pathway genes (subcohorts: A. *ATM*, B. *PALB2, and C.* other genes, e.g. *Fanconi Anemia genes, ARID1A, MER11, RAD50, NBS1, ATR*
3) Germline *BRCA1* or *BRCA2* mutations (not breast or ovarian cancer)

Principal Investigator: Sarina A. Piha-Paul, MD

Co-Principal Investigator: Funda Meric-Bernstam, MD

Institution: The University of Texas, MD Anderson Cancer Center, Department of Investigation Cancer Therapeutics

Sponsor: The University of Texas MD Anderson Cancer Center

This is an investigator-initiated study. The principal investigator, Sarina A. Piha-Paul, MD (who may also be referred to as the sponsor-investigator), is conducting the study. The University of Texas MD Anderson Cancer Center will be the IND sponsor. Therefore, the legal/ethical obligations of the principal investigator include both those of a sponsor and those of an investigator.

IND Number: 124,245

TABLE OF CONTENTS:

Title of Study 0

1. Trial Design 4

2. Objectives 5

   2.1 Primary Objective and Hypothesis 5

   2.2 Secondary Objectives 6

3. Rationale and Background 7

   3.1 Background 7

   3.2 Rationale 13

4. Methodology 16

   4.1 Entry Criteria 16

Table 1: Cohort Enrollment Algorithm 17

   4.2 Trial Treatment 21

Table 2: Regimen Description 21

Table 3: Dose Modification Guidelines for Drug-Related Adverse Events 22

Table 4: Talazoparib Dose Reduction for Toxicity 23

Table 5: Criteria for Temporary Withholding of Talazoparib in Association With Liver Test Abnormalities 24

Table 6: Investigations of Alternative Causes for Abnormal Liver Tests 25

Table 7: Monitoring of Liver Tests for Potential Drug-Induced Liver Injury 27

   4.3 Concomitant Medications 28

   4.4 Supportive Care 29

   4.5 Diet/Activity/Other Considerations 30

   4.6 Subject Withdrawal/Discontinuation Criteria 32

   4.7 Cohort Management 33

4.8 Beginning and End of Trial 33

5. Trial Flow Chart 34

6. Trial Information and Procedures 36

6.1 Trial Design 36

6.2 Treatment Plan 36

6.3 Trial Procedures 37

6.4 Assessing, Recording, and Reporting Adverse Events 51

6.5 Data Collection 56

7. Statistical Considerations 57

7.1 Statistical Analysis Plan Summary 57

1. TRIAL DESIGN

This is a single-center, non-randomized, multi-cohort trial of talazoparib (BMN 673) in subjects with advanced solid tumors with no curative therapeutic options. Subjects will be enrolled on one of the following five solid tumor cohorts:

1) Somatic mutations or deletions of *BRCA1* or *BRCA2*
2) Mutations or homozygous deletions in other *BRCA* pathway genes
    a. *ATM*
    b. *PALB2*
    c. other genes, e.g. *Fanconi Anemia genes, ARID1A, MER11, RAD50, NBS1, ATR*
3) Germline *BRCA1* or *BRCA2* mutations (not breast or ovarian cancer)

Up to approximately 150 patients will be enrolled in this trial to examine the safety and efficacy in these cohorts treated with talazoparib at 1 milligram (mg) orally per day. Subjects will be evaluated every 8 weeks (56 days +/- 7 days) with radiographic imaging to assess response to treatment. After 6 months, radiographic imaging may be evaluated every 12 weeks (84 days +/- 7 days) per investigator's decision. RECIST 1.1 will be used as the primary efficacy endpoint of response rate.

The primary objective of the trial is to determine whether the PARP inhibitor talazoparib achieves clinical benefit (complete response (CR), partial response (PR) or stable disease (SD) >24 weeks) in metastatic or inoperable locally advanced or locally recurrent cancer patients who have somatic mutations or deletions of *BRCA1* or *BRCA2*, mutations or homozygous deletions in other *BRCA* pathway genes, and germline mutations in *BRCA1* or *BRCA2* with cancers other than breast or ovarian cancer.

Secondary objectives include safety and tolerability, determination of progression free survival (PFS), duration of response (DOR), and overall survival (OS). Also included in

secondary objectives are determination of baseline molecular markers (DNA, RNA and protein) that may predict clinical benefit, determination of pharmacodynamics markers in blood and plasma that may predict outcome, determination of concordance of *BRCA1/2* alterations and determination of the concordance of genomic alterations in tumor and circulating free DNA.

Figure 1 Trial Schema

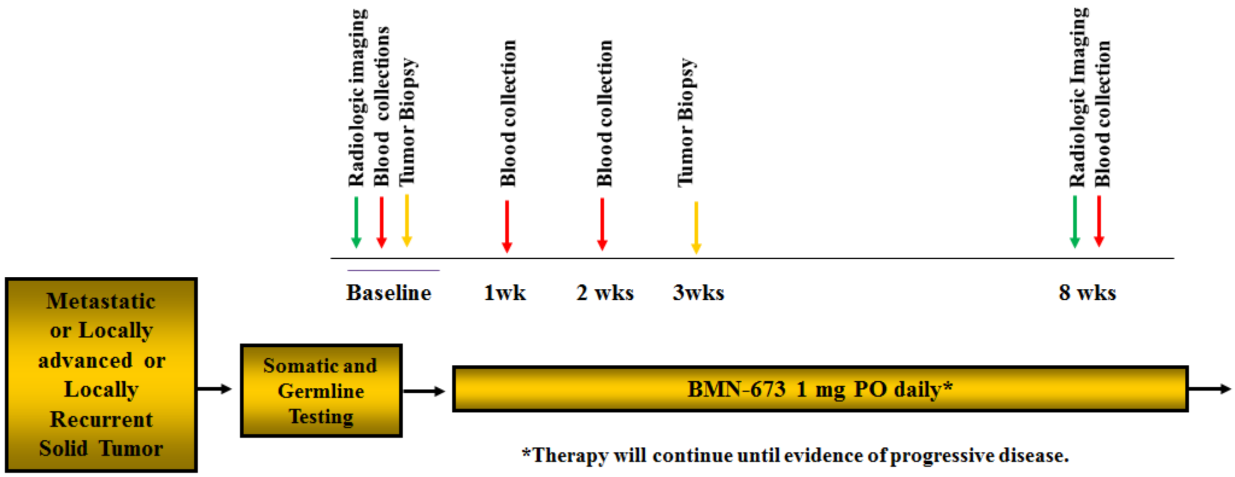

2. OBJECTIVES

2.1 Primary Objective and Hypothesis

Primary Objective: To determine whether the PARP inhibitor talazoparib achieves clinical benefit (CR, PR or SD>24 weeks) in metastatic or inoperable locally advanced or locally recurrent cancer patients who have somatic mutations or deletions of *BRCA1* or *BRCA2*, mutations or homozygous deletions in other *BRCA* pathway genes, and germline mutations in *BRCA1* or *BRCA2* with cancers other than breast or ovarian cancer.

Hypothesis: Oral administration of single agent talazoparib to subjects with advanced solid tumor and homologous repair defects as defined by a pre-identified molecular aberration (listed below) will result in clinical benefit.

The primary objective will be tested separately in each of the cohorts listed below:

1) Somatic mutations or deletions of *BRCA1* or *BRCA2*
2) Mutations or homozygous deletions in Other *BRCA* Pathway Genes
   a. *ATM*
   b. *PALB2*
   c. other genes, e.g. *Fanconi Anemia genes, ARID1A, MER11, RAD50, NBS1, ATR*
3) Germline *BRCA1* or *BRCA2* mutations (not breast or ovarian cancer)

2.2 Secondary Objectives

Across-Indication Secondary Objective

1) To evaluate the safety and tolerability of talazoparib in this patient population.

Within-Indication Secondary Objectives

The following secondary objective will be evaluated separately in each of the nine cohorts listed in Section 1.1 as well as analyzed across indications.

1) To determine baseline molecular markers (DNA, RNA and protein) or scores (e.g., microsatellite instability positives, and somatic mutation burden) that may predict clinical benefit.
2) To determine pharmacodynamic (PD) markers in tumor, blood and plasma that may predict outcome.
3) To determine concordance of *BRCA1/2* alterations in archival tissue and pre-treatment biopsies.
4) To determine concordance of genomic alterations in tumor and circulating free DNA.

5) To evaluate the progression free survival (PFS) in patients.
6) To evaluate the duration of response (DOR) in patients.
7) To evaluate the overall survival (OR) in patients.

3. RATIONALE AND BACKGROUND

3.1 Background

Refer to the Investigator's Brochure (IB) for detailed background information on talazoparib.[1]

3.1.1 Pharmaceutical and Therapeutic Background

Cancer cells frequently have defects in deoxyribose nucleic acid (DNA) repair pathways.[2,3] Cells deficient in breast cancer (*BRCA*) 1 or *BRCA2* function have a high level of chromosomal instability.[4-6] Poly (ADP-ribose) polymerase (*PARP*) inhibitors have been shown to be selectively lethal to cells lacking functional *BRCA1* or BRCA2 with minimal toxicity to normal cells.[7] Further, patients with germline *BRCA* mutations have tumors that have demonstrated defects in homologous recombination (HR) and have been shown in several trials to have response to PARP inhibitors.[8,9]

Talazoparib is a potent PARP1/2 inhibitor. Talazoparib exhibits selective anti-tumor cytotoxicity and elicits DNA repair biomarkers at much lower concentrations than earlier generation PARP1/2 inhibitors (such as olaparib, rucaparib and veliparib).[10] Talazoparib is generally well tolerated with the most common side-effects, occurring in 20-30% of patients, being fatigue, nausea and alopecia.[11] Anemia, thrombocytopenia, and neutropenia have also been observed, including grade 3-4 events.[1] Further, talazoparib has good oral bioavailability and a long half-life supporting once daily dosing.[11]

Currently several trials are evaluating the efficacy of PARP inhibitors in patients with germline *BRCA* mutations.[12,13] However, there is a great interest in identifying other patients that may benefit from PARP inhibitors.[3] In our ongoing genomic profiling studies, we have identified patients with somatic mutations, homologous deletions and loss of heterozygosity (LOH) in *BRCA1* and *BRCA2* in several tumor types. Of 248 patients that we have analyzed, we found *BRCA* mutations or deletions in patients with a variety of tumor types including breast cancer, colon cancer, hepatocellular cancer, renal cancer, ovarian cancer, endometrial cancer, soft tissue sarcoma, squamous cell of the head and neck, non-small cell lung cancer, adrenocortical carcinoma, melanoma and primary central nervous system malignancy.

3.1.1.1 **Mutations/Deletions in Other BRCA Pathway Genes**

Preclinical studies suggest that cancer cells with mutations or deletions in other *BRCA* pathway genes may preferentially benefit from treatment with PARP inhibitors.

The ataxia telangiectasia mutated (ATM) kinase is a key protein in DNA damage response.[14] ATM is recruited and activated by DNA double-strand breaks where it phosphorylates several proteins that are important in activating the DNA damage checkpoint leading to cell cycle arrest, DNA repair or apoptosis. Heterozygous germline mutation of *ATM* is a moderate-risk factor for developing breast cancer.[14] Montani and colleagues found that ATM-depletion can sensitize breast cancer cells to PARP inhibition thus PARP inhibition could be of benefit in patients with low ATM protein expression/activity such as would be seen in heterozygous carriers of germline *ATM* mutation.[14]

The MRN complex is a protein complex comprised of Mre11, Rad50, and Nbs1. This complex plays an important role in the initial processing of double-strand DNA breaks prior to repair by homologous recombination or non-homologous end joining. The *Mre11* gene encodes a nuclear protein involved in homologous recombination, telomere length maintenance, and DNA double-strand break repair. *Mre11* is required for repair

of DNA double-strand breaks by a process that likely involves homologous recombination.[15] The proteins encoded by the *RAD50* gene and *Nbs1* (also known as Nibrin) are involved in DNA double-strand break repair as well. Aberrations of the MRN complex have been found to sensitize cancer cells to PARP inhibition.[16,17]

The partner and localizer of *BRCA2* (*PALB2*) gene encodes for a protein that functions in repair of double stranded DNA breaks. Patients with variants of the *PALB2* gene have an increased risk of developing breast cancer.[18] Further, cells deficient in *PALB2* have been found to be sensitive to PARP inhibitors.[19]

Other genes may be relevant to enhanced sensitivity to PARP inhibition. Overexpression of *AURKA* was found by Sourisseau and colleagues to increase sensitivity to PARP inhibition.[20] It has also be suggested that amplification of EMSY, which can act by silencing *BRCA2*, may also lead to enhanced sensitivity to PARP inhibitors.[21] Finally deficiency in ATR can induce sensitivity to PARP inhibition.[22] Interestingly, a recent study found that ARID1A deficiency sensitizes cancer cells to PARP inhibitors.[23] Next-generation sequencing has identified *ARID1A* (AT-rich interactive domain 1A) as one of the most frequently mutated genes in human cancers.[24-26] ARID1A (also known as BAF250A) is a SWI/SNF chromatin remodeling complex subunit.[27,28] The SWI/SNF complex repositions, ejects or exchanges nucleosomes which modulates chromatin structure and DNA accessibility.[29-31] Under DNA damage, ARID1A is recruited to double stranded breaks (DSBs) via ATR interactions and facilitates DSB end processing and sustains ATR activation in response to DSBs.[23] ARID1A deficiency impairs ATR activation and repair of DNA DSBs, which sensitizes PARP inhibitor induced DSBs and cell death. [23]

Additional genes such as Fanconi Anemia (FA) genes may also be of interest.[32] The identification of the *FANCD1* gene as *BRCA2* provided the first direct link between FA proteins and DNA repair.[32,33] As BRCA2 deficient cells are sensitive to PARP inhibition, it is of interest to determine whether or not tumors that have deleterious alterations of the FA genes would share this trait.

3.1.1.2 RNA and Protein Biomarkers of Response to PARP Inhibitors

Several groups have identified additional potential predictors of response to PARP inhibitors including a *BRCA*-ness transcriptional signature (Lin lab, manuscript under review), a seven gene transcriptome reported to be: *BRCA1*, *MRE11A*, *NBS1*, *TDG*, and *XPA* mRNA conferring resistance and whose transcript levels were associated with resistance and *CHEK2* and MK2 mRNA conferring sensitivity,[40] as well as proteomic markers FOXO3 and pS209 eIF4E expression and "DNA repair score" on reverse phase protein arrays (RPPA).[41,42]

We propose to test these predictive markers as an exploratory endpoint. We hypothesize that tumors with decreased levels of *BRCA1, MRE11A, NBS1, TDG* and *XPA* will be less sensitive to PARP inhibition. We also hypothesize that tumors with increased levels of *CHEK2* and *MK2* will be sensitive to PARP inhibition. We hypothesize that baseline proteomic signature and pharmacodynamic response to talazoparib as assessed by RPPA may correlate with talazoparib growth inhibitory effects.

3.1.2 Pre-clinical and Clinical Trials

3.1.2.1 Preclinical

Talazoparib has been shown to be a highly selective and potent cytotoxic agent in human cancer cell lines harboring mutations that compromise DNA repair pathways. In the *BRCA1*-deficient breast cancer MX-1 xenograft tumor model, daily talazoparib administration resulted in significant tumor growth delay and or regression. There were no talazoparib-related effects on respiratory or CNS parameters after a single oral administration to rats or cardiovascular parameters after a repeat dose oral administration to dogs.

For more details, please refer to the IB.

3.1.2.2 Clinical Trial Introduction

Data from three studies as of 30 November 2016 are summarized below:

- PRP-001, initiated on 3 January 2011, is a single-arm, open-label study to assess the safety, pharmacokinetics (PK), pharmacodynamics (PD), and preliminary efficacy of talazoparib in patients with advanced tumors with DNA-repair pathway abnormalities, particularly those associated with *BRCA*- and *PTEN*-dysfunction. The initial cohort of patients was treated with 25 µg talazoparib once daily. As of 30 November 2016, the study has been completed in full with a total of 110 patients enrolled in the study (7 subjects remain on study drug).

- 673-201, initiated 13 December 2013, is a two-arm, open-label study o evaluate the safety and efficacy of talazoparib (also known as BMN 673) in subjects with locally advanced or metastatic breast cancer with a deleterious germline BRCA 1 or BRCA 2 mutation. Subjects will be assigned to either Cohort 1 or 2 based on prior chemotherapy for metastatic disease: Cohort 1) Subjects with a documented PR or CR to a prior platinum-containing regimen for metastatic disease with disease progression > 8 weeks following the last dose of platinum; or Cohort 2) Subjects who have received > 2 prior chemotherapy regimens for metastatic disease and who have had no prior platinum therapy for metastatic disease. All patients were treated with 1 mg talazoparib once daily. As of 30 November 2016, a total of 83 patients have been rolled in the study (which has been closed to enrollment).

- MDV3800-14, initiated on 13 October 2016, is a single-arm, open-label study to evaluate the effects of talazoparib on cardiac repolarization in patients with advanced solid tumors with no available standard treatment options. All patients

were treated with 1 mg talazoparib once daily. As of 30 November, 2016, a total of 4 patients have been enrolled in the study.

3.1.3 Summary of Interim Safety Results

3.1.3.1 Adverse Event Summary

Approximately 439 patients and 18 healthy volunteers have received talazoparib in company-sponsored studies as of the data cutoff date of the July 2017 investigator's brochure (30 November 2016). Aggregate safety data from 3 open-label, company-sponsored clinical studies evaluating talazoparib monotherapy at the proposed dose of 1 mg/day (PRP-001, 673-201, and MDV3800-14; N = 164 patients) as of 30 Nov 2016, provide the basis for the reported treatment-emergent adverse events (TEAEs).

TEAEs in ≥20% of the 164 patients who received 1 mg/day talazoparib were related to myelosuppression (anemia, thrombocytopenia, neutropenia), GI toxicity (nausea, diarrhea, constipation), fatigue, alopecia, and headache. Grade ≥3 TEAEs in ≥5% of patients were associated with myelosuppression.

Study drug-related TEAEs occurring in ≥20% of patients who received 1 mg/day talazoparib were anemia (42.1%), fatigue (36.6%), nausea (29.3%), thrombocytopenia (25.6%), neutropenia (20.7%), and alopecia (20.1%). Grade ≥3 drug-related TEAEs occurring in ≥5% of patients were anemia (28.0%), thrombocytopenia (16.5%), and neutropenia (12.2%).

Five of 164 patients (3.0%) who received 1 mg/day talazoparib discontinued study drug due to a TEAE. The events that led to study drug discontinuation were anemia, increased alanine aminotransferase (ALT), increased AST, metastatic breast cancer, and dyspnea.

3.1.3.2 Serious Adverse Events (SAE)

Serious adverse events (SAEs) occurred in 52 of 164 patients (31.7%) who received 1 mg/day talazoparib. SAEs occurring in ≥2% of patients were pleural effusion (4.3%), anemia and dyspnea (3.7% each), and neoplasm progression and thrombocytopenia (2.4% each). Fourteen patients had SAEs considered related to study drug, which included anemia (3.0%); thrombocytopenia (2.4%); platelet count decreased (1.2%); and increased transaminases, neutropenic sepsis, and vomiting (0.6% each).

Most SAEs occurred in single patients, except that pain and pain in extremity each occurred in 2 patients. One patient discontinued study drug due to an adverse event of chronic kidney disease.

A total of 12 of 164 patients who received 1 mg/day talazoparib had a TEAE that led to death (6 associated with malignancies including 1 also associated with bronchopneumonia; 2 dyspnea; and 1 each disease progression, lung infection, hypoxia, and respiratory failure). Of these, none was assessed as related to study drug by the investigator.

3.2 Rationale

3.2.1 Rationale for the Trial and Selected Subject Population

Details regarding specific benefits and risks for subjects participating in this clinical trial may be found in the accompanying Investigators Brochure (IB) and Informed Consent documents.

This is a single center, nonrandomized, multi-cohort trial of talazoparib in subjects with advanced solid tumors with no curative therapeutic options. Subjects will be enrolled into 1 of 5 solid tumor indications as outlined in Section 1.1. Given that cells deficient in *BRCA1/2* have a high level of chromosomal instability, PARP inhibition may be relevant in a variety of solid tumors in addition to the ones previously studied. Exploration as to which of these tumors might be responsive to PARP inhibition is being pursued in this protocol. Five indications were chosen for study. This indication discovery effort may

lead to a better understanding of which genomic alterations may be more responsive to talazoparib.

A patient must have one of the genetic alterations necessary for enrollment (per inclusion criteria) pre-identified by a CLIA certified laboratory prior to consideration for participation in this trial.

3.2.2 Rationale for Dose Selection/Regimen/Modification

An open label Phase I trial was conducted to evaluate the safety and clinical activity of single agent talazoparib .[11] In this trial, a two stage dose escalation study was used with a standard 3+3 design. In the dose escalation portion of the trial (stage 1), cycle 1 was six weeks in duration with drug taken on day 1 and days 8-35 of the cycle for pharmacokinetic and pharmacodynamics assays, followed by daily and continuous dosing in 4 week cycles. Stage 2 was an expansion at the maximum tolerated dose (MTD) in patients with tumors defective in DNA repair.

The dose escalation portion of this trial enrolled 39 patients in nine dose levels from 25 to 1100 µg/d. An MTD of 1000 µg/d was determined as dose-limiting thrombocytopenia occurred in 1/6 and 2/5 patients at 900 and 1100 µg/d, respectively.

Pharmacodynamic data showed inhibition of PARP activity in PBMCs was observed at doses ≥ 100 µg/d. Pharmacokinetic data analysis showed talazoparib plasma concentrations peaked at 1-2 hours post-dose and exposure increased proportionally to the dose. Steady state plasma concentrations were reached by the end of the 2nd week of daily dosing. The mean C_{max} ranged from 0.30 - 25.4 ng/mL and AUC_{0-24} ranged from 3.96 - 203 ng-hr/mL across the 25 to 1100 µg/d dose range after 28 days of daily dosing.

3.2.3 Rationale for Endpoints

3.2.3.1 **Efficacy Endpoints**

The primary efficacy objective of this study is to determine whether the PARP inhibitor talazoparib achieves clinical benefit (CR, PR or SD>24 weeks) in metastatic or inoperable locally advanced or locally recurrent cancer patients who have somatic mutations or deletions of *BRCA1* or *BRCA2*, mutations or homozygous deletions in other *BRCA* pathway genes, and germline *BRCA* mutations with cancers other than breast or ovarian cancer.

RECIST 1.1[38] as assessed by the investigator will be used as the primary response rate efficacy endpoint. RECIST 1.1 will also be used by the local site to determine eligibility and make treatment decisions.

3.2.3.2 **Safety Endpoints**

The secondary safety objective of this study is to characterize the safety and tolerability of talazoparib in subjects with advanced solid tumors. The safety analysis will be based on subjects who experienced toxicities as defined by CTCAE criteria. Safety will be assessed by quantifying the toxicities and grades experienced by subjects who have received talazoparib, including serious adverse events (SAEs).

Safety will be assessed by reported adverse experiences using CTCAE, Version 4.0. The attribution to drug, time-of-onset, duration of the event, its resolution, and any concomitant medications administered will be recorded. AEs will be analyzed including but not limited to all AEs, SAEs, fatal AEs, and laboratory changes.

3.2.3.3 **Biomarker Research**

Additional biomarker research to identify factors important for talazoparib therapy will also be pursued. For example, tumor and blood samples from this study will undergo genomic, transcriptomic and proteomic analyses. Additional research may evaluate factors important for predicting responsiveness or resistance to talazoparib therapy.

4. METHODOLOGY

4.1 Entry Criteria

4.1.1 Diagnosis/Condition for Entry into the Trial

Male/female subjects of at least 18 years of age and with advanced solid tumors who have undergone CLIA certified testing revealing results fitting one of the following cohorts will be enrolled in this trial.

1) Somatic mutations or deletions of *BRCA1* or *BRCA2*
2) Mutations or homozygous deletions in Other *BRCA* Pathway Genes
    a. *ATM*
    b. *PALB2*
    c. other genes, e.g. *Fanconi Anemia genes, ARID1A, MER11, RAD50, NBS1, ATR*
3) Germline *BRCA1* or *BRCA2* mutations (not breast or ovarian cancer)

4.1.2 Subject Inclusion Criteria

In order to be eligible for participating in this trial, the subject must meet the following criteria:

1) Patients with advanced or metastatic cancer that is refractory to standard therapy or has relapsed after standard therapy.

2) Patients must have one of the following (see Table 1):
   - somatic mutations or deletions in *BRCA1* or *BRCA2*,

- genomic alterations in other *BRCA* pathway genes (subcohorts: a. *ATM*, b. *PALB2, and c.* other genes, e.g. *Fanconi Anemia genes, ARID1A, MER11, RAD50, NBS1, ATR*)
- germline mutation in *BRCA1* or *BRCA 2* (not breast or ovarian cancer)

Table 1: Cohort Enrollment Algorithm

Cohort	Mutation/ Alteration*	Included	Excluded (if known)	Other Exclusions (if applicable)
1	Somatic Mutations or Deletions of *BRCA 1/2*	N/A	N/A	Not Breast or Ovarian Cancer with Germline *BRCA* Mutations
2	Alterations in Other *BRCA* Pathway Genes	Mutations or Deletions in:<br>*a. ATM*<br>*b. PALB2*<br>c. Other Genes (e.g. Fanconi Anemia Genes, ARID1A, MER11, RAD50, NBS1, ATR)	N/A	Not Breast or Ovarian Cancer with Germline *BRCA* Mutations
3	Germline Mutations of *BRCA 1/2*	N/A	N/A	Not Breast or Ovarian Cancer
*mutations or alterations predicted to be deleterious				

- **Somatic Analysis of *BRCA* Pathway Genes:**
  Somatic mutation analysis can be done by a CLIA validated approach. This approach expected to be next generation sequencing by targeted exome sequencing (Ion Proton, or Illumina platforms) and may be through commercial multigene assays such as Foundation One (Foundation Medicine), Oncomine (Life technologies), Myriad Genetics, Inc, etc. Patients with single somatic mutation testing through Sanger sequencing, pyrosequencing, or alternate platform in the CLIA environment will also be eligible.

  Copy number changes orhomozygous deletion of *BRCA1/2, ATM; PALB2; MER11, RAD50; NBS1, ATR,* and *Fanconi Anemia genes* can be determined by a CLIA test including targeted exome sequencing, FISH a PCR-based copy number assay or a copy number array.

- **Cohort Assignment of Patients with Germline Alterations:**
  Patients with breast or ovarian cancer will be eligible if they do not have germline *BRCA1/2* mutations. Patients with other tumor types and mutations in *BRCA1/2* can be initiated on treatment prior to germline testing, but germline testing will be performed for cohort stratification (i.e. to determine if the *BRCA1/2* mutation is somatic or germline).

  Germline testing will have been done locally as part of standard-of-care for patients on the germline cohort (cohort 3). All other cohorts will have germline testing in the research environment after informed consent.

  Patients with somatic as well as deleterious germline alterations in *ATM; PALB2; MER11, RAD50; NBS1, ATR* will be eligible for cohort 2.

- **Cohort Assignment of Patients with Multiple Alterations:**
  Patients with more than one alteration will be assigned as follows:
  - Patients with germline *BRCA1/2* alterations (not breast or ovarian cancer) will be assigned to cohort 3, regardless of other alterations.
  - Patients with somatic *BRCA1/2* mutations will be assigned to cohort 1, regardless of other somatic alterations.
  - Patients with somatic *BRCA1/2* deletions will be assigned to cohort 1, in the absence of *BRCA* somatic or germline mutations.
  - Patients with mutations or deletions in *ATM; PALB2; Fanconi Anemia genes; ARID1A; MER11; RAD50; NBS1;* and *ATR*; or amplification of EMSY will be assigned to cohort 2, in the absence of other *BRCA* alterations.

3) Patients must be ≥18 years of age.

4) Patients must have measurable disease by RECIST 1.1.

5) Eastern Cooperative Oncology Group (ECOG) performance status (PS) 0-1.

6) Adequate organ function as defined below:

- **Hematological**
  - Absolute neutrophil count (ANC) ≥1,500 /mcL
  - Platelets ≥100,000 / mcL
  - Hemoglobin ≥9 g/dL or ≥5.6 mmol/L
- **Renal**
  - Serum creatinine ≤1.5xULN OR Measured or calculated creatinine clearance (GFR can also be used in place of creatinine or CrCl) ≥60 mL/min for subject with creatinine levels >1.5x institutional ULN [Creatinine clearance should be calculated per institutional standard]
- **Hepatic**
  - Serum total bilirubin ≤1.5xULN OR Direct bilirubin ≤ULN for subjects with total bilirubin levels >1.5xULN
  - AST (SGOT) and ALT (SGPT) ≤2.5xULN OR ≤5xULN for subjects with liver metastases
- **Coagulation**
  - International Normalized Ratio (INR) or Prothrombin Time (PT) ≤1.5xULN unless subject is receiving anticoagulant therapy as long as PT or PTT is within therapeutic range of intended use of anticoagulants
  - Activated Partial Thromboplastin Time (aPTT) ≤1.5xULN unless subject is receiving anticoagulant therapy as long as PT or PTT is within therapeutic range of intended use of anticoagulants

7) Patients must be ≥4 weeks beyond treatment with any chemotherapy or other investigational therapy to include hormonal, biological, or targeted agents; or at least 5 half-lives from hormonal, biological, or targeted agents, whichever is shorter at the time of treatment initiation.

8) Women of child-bearing potential MUST have a negative serum or urine HCG test unless prior tubal ligation (>/= 1 year before screening), total hysterectomy or menopause (defined as 12 consecutive months of amenorrhea). Patients should not become pregnant or breastfeed while on this study. Sexually active patients must

agree to use dual contraception for the duration of study participation and for 120 days after the last dose of talazoparib.

9) Ability to understand and willingness to sign informed consent form prior to initiation of the study and any study procedures;

10) Patients need to have biopsiable disease to enroll on cohort 1-2. Patients eligible for Cohort 3 with a germline *BRCA* alteration can be enrolled even if they do not have biopsiable disease.

4.1.3 **Subject Exclusion Criteria**

The subject must be excluded from participating in the trial if the subject meets one of the following criteria:

1) Patients who are pregnant or breastfeeding;

2) Prior treatment with a PARP inhibitor;

3) Known Hepatitis B, Hepatitis C or HIV infection;

4) Inability or unwillingness to swallow pills.

5) Active infection requiring intravenous (IV) antibiotics or other uncontrolled intercurrent illness requiring hospitalization.

6) Any medical condition or diagnosis that would likely impair absorption of an orally administered drug (e.g. gastrectomy, ileal bypass, chronic diarrhea, gastroparesis).

7) Inability to comply with the study and follow-up procedures.

8) History of CVA, myocardial infarction or unstable angina within the previous 6 months before starting therapy.

9) Has a known additional malignancy that is progressing or requires active treatment. Exceptions include basal cell carcinoma of the skin, squamous cell carcinoma of the skin that has undergone potentially curative therapy or in situ cervical cancer.

10) Has a known psychiatric or substance abuse disorders that would interfere with cooperation with the requirements of the trial.

11) Has known active central nervous system (CNS) metastases and/or carcinomatous meningitis. Subjects with previously treated brain metastases may participate provided they are stable (without evidence of progression by imaging for at least four weeks prior to the first dose of trial treatment and any neurologic symptoms have returned to baseline), have no evidence of new or enlarging brain metastases, and are not using steroids for at least 7 days prior to trial treatment. This exception does not include carcinomatous meningitis which is excluded regardless or clinical stability.

4.2 Trial Treatment

The study drug dose and schedule to be used in this trial are outlined below in Table 2.

Table 2: Regimen Description

Agent	Route	Schedule	Cycle Length
Talazoparib<br>(PF-0644076; MDV3800; BMN 673)	oral	1 mg po daily	28 days

Talazoparib will be provided to the investigator by Pfizer in capsule form in 0.25 mg strengths. Talazoparib should be swallowed whole and taken at approximately the

same time each day. Talazoparib can be administered regardless of food intake. Talazoparib should be stored at room temperature (15-30°C or 59-89°F). Unused, expired, or returned talazoparib will be disposed of as per MDACC policy.

4.2.1 Dose Selection

The rationale for selection of doses to be used in this trial is provided in Section 3.0 – Background and Rationale.

4.2.2 Dose Modification

Talazoparib will be withheld for drug-related grade 3 and greater hematologic and non-hematologic toxicities including laboratory abnormalities and severe or life-threatening AEs as per Table 3 below.

Table 3: Dose Modification Guidelines for Drug-Related Adverse Events

**Toxicity**	**Management of Adverse Events (Except Liver Abnormalities [1])**
Grade 1 or 2	No requirement for dose interruption or dose reduction.
Selected hematologic grade 3 or 4 events	
Anemia (hemoglobin < 8.0 g/dL)	Hold talazoparib and monitor weekly until hemoglobin returns to baseline grade or better. Implement supportive care per local guidelines.<br>• Talazoparib may be reduced by 1 dose level as described in Table 4.<br>If anemia persists for > 4 weeks without recovery to baseline grade, discontinue talazoparib and refer to a hematologist for evaluation, including assessment for possible MDS/AML.
Neutropenia (ANC < 1000/µL)	Hold talazoparib and monitor weekly until ANC ≥ 1500/µL. Implement supportive care per local guidelines. Resume talazoparib based on the following recovery times:<br>• ≤ 1 week: No change.<br>• > 1 week: Reduce talazoparib by 1 dose level as described in Table 4.<br>If neutropenia persists for > 4 weeks without recovery to ≥ 1500/µL, discontinue talazoparib and refer to a hematologist for evaluation, including assessment for possible MDS/AML.

Thrombocytopenia (platelets < 50,000/µL)	Hold talazoparib until platelets ≥ 75,000/µL. Implement supportive care per local guidelines. Resume talazoparib based on the following recovery times:<br>• ≤ 1 week: No change.<br>• > 1 week: Reduce talazoparib by 1 dose level as described in Table 4.<br>If thrombocytopenia persists for > 4 weeks without recovery to ≥ 75,000/µL, discontinue talazoparib and refer to a hematologist for evaluation, including assessment for possible MDS/AML.
Other grade 3 or 4 events, except abnormal liver tests [1]	Hold talazoparib as follows:<br>• For clinically significant grade 3 or 4 laboratory abnormalities, talazoparib may be held. Resume talazoparib when the laboratory abnormality resolves to grade ≤ 2 (baseline grade for creatinine increases).<br>• For clinically significant grade 3 or 4 adverse events, hold talazoparib until the adverse event resolves to grade ≤ 2. Resume talazoparib at the same dose or reduce by 1 dose level as described in Table 4 if the event resolves or improves within 4 weeks of holding talazoparib, and can be monitored if it recurs.<br>Implement supportive care per local guidelines. Contact medical monitor to discuss potential dose modification.<br>Talazoparib should be permanently discontinued for unresolved grade 3 or 4 toxicity per investigator decision that continued talazoparib treatment is not in the patient's best interest.

[1] Dose modifications for liver abnormalities are discussed in Table 5.

AML, acute myeloid leukemia; ANC, absolute neutrophil count; MDS, myelodysplastic syndrome.

The dose of talazoparib may be reduced incrementally as shown in Table 4.

Table 4: Talazoparib Dose Reduction for Toxicity

Talazoparib Dose Level	Talazoparib Dose (mg/day)
Initial dose	1.0
First dose reduction	0.75
Second dose reduction	0.50
Third dose reduction	0.25

In case toxicity does not resolve to grade 0-1 or baseline within 21 days after last dose, trial treatment should be discontinued. With principal investigator agreement, subjects with laboratory adverse event still at grade 2 after 21 days may continue treatment in the trial only if asymptomatic and controlled.

With principal investigator agreement, subjects who derive benefit from the treatment, but who have long term tolerability issues, including but not limited to the need for frequent transfusions, may switch to a 3 weeks on/1 week off schedule on the current dose level.

Dose re-escalation: Talazoparib dose re-escalation may be allowed after toxicities resolve and the reduced dose is tolerated at the discretion of the investigator.

Patients may remain in the study until the development of progressive neoplastic disease, the development of unacceptable toxicity, or fulfillment of any of the criteria for withdrawal from study as described in Section 4.6.

4.2.2.1 **Assessment of Abnormal Liver Tests**

Patients who develop abnormal liver tests (AST, ALT, total bilirubin), abnormal international normalized ratio (INR) values, or signs or symptoms of hepatitis during the study treatment period may meet the criteria for temporarily withholding or permanently discontinuing talazoparib as specified in United States (US) Food and Drug Administration (FDA) Guidance for Industry - Drug-Induced Liver Injury: Premarketing Clinical Evaluation (2009). Patients who meet criteria for permanent discontinuation or temporary withholding of talazoparib or who do not meet the criteria but who have abnormal liver tests are to be followed up according to the recommendations in this section.

Talazoparib should be withheld for any liver test abnormality listed in Table 5.

Table 5: Criteria for Temporary Withholding of Talazoparib in Association With Liver Test Abnormalities

Baseline AST or ALT Value	Elevation
≤ 3 × ULN	> 5 × ULN (ALT or AST ≥ 3 × ULN with the presence of signs and symptoms consistent with acute hepatitis and/or eosinophilia [≥ 500 eosinophils/µL])
> 3 × ULN	> 8 × ULN

Baseline Total Bilirubin Value	Elevation
≤ 1.5 × ULN	> 3 × ULN (> 5 × ULN in patients with a baseline total bilirubin value of > 1.5 × ULN and ≤ 3 × ULN [patients with Gilbert syndrome or for whom indirect bilirubin concentrations suggest an extrahepatic source of elevation])

For rechallenge, dose modification may be required per Table 3.

ALT, alanine aminotransferase; AST, aspartate aminotransferase; ULN, upper limit of normal.

Talazoparib should be withheld pending investigation of alternative causes of liver injury (Table 6). When withholding talazoparib, follow-up should continue for possible drug-induced liver injury until the liver test abnormalities resolve to baseline grade. Rechallenge may be considered if an alternative cause for the abnormal liver tests (ALT, AST, total bilirubin) is discovered and the laboratory abnormalities resolve to normal or baseline values.

Table 6: Investigations of Alternative Causes for Abnormal Liver Tests

**Recommended tests**
Complete blood count with differential to assess for eosinophilia
Serum total immunoglobulin G (IgG), antinuclear antibody (ANA), antismooth muscle antibody, liver kidney microsomal antibody 1 (LKM1), and liver cytosol type 1 antibodies (L-C-1) to assess for autoimmune hepatitis
Serum acetaminophen (paracetamol) concentration
**Obtain a more detailed history**
Prior and concurrent diseases or illness
Exposure to environmental and/or industrial chemical agents
Symptoms (if applicable) including right upper quadrant pain, hypersensitivity-type reactions, fatigue, nausea, vomiting, and fever
Prior and concurrent use of alcohol, recreational drugs, and special diets
Concomitant use of medications (including nonprescription medicines and herbal and dietary supplements), plants, and mushrooms
Obtain viral serologies for hepatitis A, B, C, and E (D if positive for hepatitis B), cytomegalovirus, Epstein-Barr virus, herpes simplex virus
**Recommended tests as clinically indicated**
Echocardiogram (ECHO)
Serum and urine copper and serum ceruloplasmin
Iron studies (serum iron and ferritin) and transferrin saturation
Serology for celiac disease
Serum alpha-1 antitrypsin

Creatine phosphokinase (CPK), haptoglobin, lactate dehydrogenase (LDH), and peripheral blood smear
Appropriate liver imaging
Hepatology consult (liver biopsy may be considered in consultation with a hepatologist)

The principal investigator and medical monitor should discuss and agree with any decision to rechallenge. Following rechallenge, patients should be closely monitored for signs and symptoms of hepatitis and/or abnormal liver test results. If signs or symptoms recur with rechallenge, talazoparib should be permanently discontinued. Rechallenge should never occur if the criteria for permanent discontinuation are clearly met.

4.2.2.2 Criteria for Permanent Discontinuation of Talazoparib in Association With Liver Test Abnormalities

Talazoparib should be discontinued permanently if all of the following 4 criteria are met (ie, potential severe drug-induced liver injury/Hy's law case):

1. AST or ALT increases to ≥ 3 times ULN (> 5 ×ULN if baseline ALT/AST is > 3 ×ULN)
2. Total bilirubin increases to > 2 times ULN or INR > 1.5
3. Alkaline phosphatase value does not reach 2 times ULN (note: in the presence of elevated alkaline phosphatase associated with bone metastases, gamma glutamyl transferase should be tested and the results should be within the reference range)
4. No alternative cause explains the combination of the above laboratory abnormalities; important alternative causes include, but are not limited to the following:
    - Hepatobiliary tract disease
    - Viral hepatitis (eg, hepatitis A/B/C/D/E, Epstein-Barr virus, cytomegalovirus, herpes simplex virus, varicella, toxoplasmosis, and parvovirus)
    - Congestive heart failure, hypotension, or any cause of hypoxia to the liver causing ischemia

- Exposure to hepatotoxic agents/drugs or hepatotoxins, including herbal and dietary supplements, plants, and mushrooms
- Alcoholic hepatitis
- Nonalcoholic steatohepatitis (NASH)
- Autoimmune hepatitis
- Wilson disease and hemochromatosis
- Alpha-1 antitrypsin deficiency

If an alternative cause for hepatotoxicity is identified or less stringent conditions developed than those noted above, then it should be determined (based on the patient population and/or severity of the hepatotoxicity or event) whether talazoparib should be withheld or permanently discontinued as appropriate for the safety of the patient. When talazoparib is temporarily withheld or permanently discontinued due to a potential drug-induced liver injury, a period of close observation is to commence until the liver test abnormalities return to baseline or normal values. The evaluations listed in Table 7 should be performed.

Table 7: Monitoring of Liver Tests for Potential Drug-Induced Liver Injury

**Results**	**Frequency for Repeating Liver (AST, ALT, Bilirubin [Total and Direct]) and INR Tests**
After the initial liver test abnormality	Within 24 hours
If AST or ALT ≥ 3 × ULN (> 5 × ULN if baseline ALT/AST is > 3 × ULN), and total bilirubin > 2 × ULN or INR > 1.5	Every 24 hours until laboratory abnormalities improve
If ALT or AST ≥ 3 × ULN (> 5 × ULN if baseline ALT/AST is > 3 × ULN) and total bilirubin or INR are normal	Every 48 to 72 hours until laboratory abnormalities improve
If the liver test abnormalities improve AND the patient is asymptomatic	Frequency may decrease

ALT, alanine aminotransferase; AST, aspartate aminotransferase; INR, international normalized ratio; ULN, upper limit of normal.

As drug-induced liver injury is a diagnosis of exclusion, it is important to initiate investigation of alternative causes for abnormal liver tests, which may include consultation with a hepatologist.

4.2.3 Timing of Dose Administration

Study drug should be administered starting on Day 1 of each cycle after all procedures/assessments have been completed as detailed on the Trial Flow Chart (Section 5). Starting from Cycle 13, the study drug should be administered on Day 1 of every 3 cycles after all procedures/assessments have been completed.

Study drug will be administered on an outpatient basis. The patient should take the medication at the same time every day. If a patient vomits after dosing, the dose will not be made up. If a patient misses their dose and remembers with </=6 hours of the scheduled time of their dose, they may take the missed dose. If however, it is >6 hours, the patient should skip that dose and restart with the next scheduled dose.

4.3 Concomitant Medications

4.3.1 Acceptable Concomitant Medications

All treatments that the investigator considers necessary for a subject's welfare may be administered at the discretion of the investigator in keeping with the community standards of medical care. All concomitant medication will be recorded in the electronic medical record including all prescription, over-the-counter (OTC), herbal supplements, and IV medications and fluids. If changes occur during the trial period, documentation of drug dosage, frequency, route, and date may also be included in the electronic medical record.

All concomitant medications received within 28 days before the first dose of trial treatment and 30 days after the last dose of trial treatment should be recorded.

Concomitant medications administered after 30 days after the last dose of trial treatment should be recorded as well.

4.3.2 Prohibited Concomitant Medications

Subjects are prohibited from receiving the following therapies during the Screening and Treatment Phase of this trial:

- Anti-cancer systemic chemotherapy or biological therapy
- Chemotherapy not specified in this protocol
- Investigational agents other than talazoparib
- Radiation therapy

Note: Radiation therapy to a symptomatic solitary lesion may be allowed.

Subjects who, in the assessment by the investigator, require the use of any of the aforementioned treatments for clinical management should be removed from the trial. Subjects may receive other medications that the investigator deems to be medically necessary.

There are no prohibited therapies during the Post-Treatment Follow-up Phase.

4.4 Supportive Care

4.4.1 Supportive Care Guidelines

Subjects should receive appropriate supportive care measures as deemed necessary by the treating investigator including but not limited to the items outlined below:

- Diarrhea: All subjects who experience diarrhea should be advised to drink liberal quantities of clear fluids. If sufficient oral fluid intake is not feasible, fluid and electrolytes should be substituted via IV infusion. Consideration should be given

for the administration of prophylactic antiemetic therapy according to standard institutional practice.

- Nausea/vomiting: Nausea and vomiting should be treated aggressively, and consideration should be given for the administration of prophylactic antiemetic therapy according to standard institutional practice. Subjects should be strongly encouraged to maintain liberal oral fluid intake.
- Anti-infectives: Subjects with a documented infectious complication should receive oral or IV antibiotics or other anti-infective agents as considered appropriate by the treating investigator for a given infectious condition, according to standard institutional practice.

4.5 Diet/Activity/Other Considerations

4.5.1 Diet

Subjects should maintain a normal diet unless modifications are required to manage an AE such as diarrhea, nausea or vomiting.

4.5.2 Contraception

Talazoparib may have adverse effects on a fetus in utero. Furthermore, it is not known if talazoparib has transient adverse effects on the composition of sperm. Non -pregnant, non-breast- feeding women may be enrolled if they are willing to use 2 methods of birth control or are considered highly unlikely to conceive. Highly unlikely to conceive is defined as 1) surgically sterilized, or 2) postmenopausal (a woman who is ≥45 years of age and has not had menses for greater than 1 year will be considered postmenopausal) , or 3) not heterosexually active for the duration of the study.

The two birth control methods can be either two barrier methods or a barrier method plus a hormonal method to prevent pregnancy. Subjects should start using birth control from study Visit 1 throughout the study period up to 120 days after the last dose of study therapy.

The following are considered adequate barrier methods of contraception: diaphragm, condom (by the partner), copper intrauterine device, sponge, or spermicide, as per local regulations or guidelines. Appropriate hormonal contraceptives will include any registered and marketed contraceptive agent that contains an estrogen and/or a progestational agent (including oral, subcutaneous, intrauterine, or intramuscular agents).

Subjects should be informed that taking the study medication may involve unknown risks to the fetus (unborn baby) if pregnancy were to occur during the study. In order to participate in the study they must adhere to the contraception requirement (described above) for the duration of the study and during the follow-up period. If there is any question that a subject will not reliably comply with the requirements for contraception, that subject should not be entered into the study.

4.5.3 Use in Pregnancy

If a subject inadvertently becomes pregnant while on treatment with talazoparib, the subject will immediately be removed from the study. The site will contact the subject at least monthly and document the subject’s status until the pregnancy has been completed or terminated. The outcome of the pregnancy will be reported to Pfizer without delay and within 24 hours if the outcome is a serious adverse experience (e.g., death, abortion, congenital anomaly, or other disabling or life-threatening complication to the mother or newborn). The study investigator will make every effort to obtain permission to follow the outcome of the pregnancy and report the condition of the fetus or newborn to Pfizer. If a male subject impregnates his female partner the study personnel at the site must be informed immediately and the pregnancy reported to Pfizer and followed.

4.5.4 Use in Nursing Women

It is unknown whether talazoparib is excreted in human milk. Since many drugs are excreted in human milk, and because of the potential for serious adverse reactions in the nursing infant, subjects who are breast-feeding are not eligible for enrollment.

4.6 Subject Withdrawal/Discontinuation Criteria

Subjects may withdraw consent at any time for any reason or be dropped from the trial at the discretion of the investigator should any untoward effect occur. In addition, a subject may be withdrawn by the investigator if enrollment into the trial is inappropriate, the trial plan is violated, or for administrative and/or other safety reasons.

A subject must be discontinued from the trial for any of the following reasons:

- The subject or legal representative (such as a parent or legal guardian) withdraws consent.

A subject must be discontinued from treatment (but may continue to be monitored in the trial) for any of the following reasons:

- The subject or legal representative (such as a parent or legal guardian) withdraws consent for treatment
- Radiographic disease progression
- Unacceptable adverse experiences
- Intercurrent illness that prevents further administration of treatment
- Investigator's decision to withdraw the subject
- The subject has a confirmed positive serum pregnancy test
- Noncompliance with trial treatment or procedure requirements
- The subject is lost to follow-up
- Administrative reasons

Follow-up visit procedures are listed in Section 5 (Protocol Flow Chart). After the end of treatment, each subject will be followed for 30 days for adverse event monitoring

(serious adverse events will be collected for 90 days after the end of treatment). Subjects who discontinue for reasons other than progressive disease will have post-treatment follow- up for disease status until disease progression, initiating a non-study cancer treatment, withdrawing consent or becoming lost to follow-up. After documented disease progression each subject will be followed by telephone for survival status for a maximum of 12 months after their last dose of study medication.

4.7 Cohort Management

Additional subjects may be enrolled in a given cohort to ensure that the required number of evaluable subjects in each cohort is achieved. An evaluable subject is any subject that is deemed eligible for the study and has received study drug. A subject who discontinues without completing the first tumor assessment (radiographic evaluation approximately 8 weeks after baseline) will be replaced. However, a subject that discontinues the trial for progressive disease or a drug-related AE will not be replaced and will be counted in the evaluable population of subjects for the respective cohort.

4.8 Beginning and End of Trial

The study begins when the first subject signs the informed consent. The end of the study may be designated as the time point when all subjects have discontinued the study or are a minimum of 6 months post initial study medication administration. If, by the end of the study, there remains at least 1 subject still on study treatment for at least 6 months, the subject(s) may enter additional treatment cycles. At this point a database lock of the trial may occur to allow the analysis of the study data. Any remaining subjects may continue to receive study medication and be seen by the investigator per usual standard of care for this subject population. In addition, the investigator will be expected to monitor for and report any serious adverse events, and pregnancies, as detailed in Section 6.4.1.1 (Serious Adverse Events). The subject is considered on study until such time that he/she meets any of the discontinuation criteria.

5. TRIAL FLOW CHART

Dosing, laboratory tests, imaging studies and office visits will occur per protocol (within ± 4 days) unless patients' medical or logistical issues necessitate adjustment.

Assessment Tool	Baseline (within 4 weeks of C1D1)	Cycle 1				Cycle 2 to Cycle 12				Cycle 13 and Beyond[J]		End of Treatment Visit	Safety Follow-Up Visit [C]	Survival Follow-Up
		Week				Week				Q4W	Q12W	At time of Discontinuation	30 (± 5) Days Post Discontinuation	Every 12 weeks for 1 year
		1	2	3	4	1	2	3	4					
Review Adverse Events	X	X	X	X	X	X					X	X	X	
History and Physical Exam	X	X	X	X	X	X					X	X		
ECOG Assessment	X	X				X					X	X		
Vital Signs	X	X				X					X	X		
CBC with differential*	X	X	X	X	X	X	X	X	X	X		X	X	
Sodium, Potassium, Chloride, Bicarbonate, BUN, Creatinine, Glucose, Calcium, Magnesium	X	X	X	X	X	X				X		X	X	
Albumin, Alkaline Phosphatase, Total Bilirubin, SGOT [AST], SGPT [ALT]	X	X	X	X	X	X				X		X	X	
Urinalysis	X	X				X				X		X	X	
PT/PTT	X^{H}											X		
Serum Pregnancy Test (in women with childbearing potential)	X^{H}													
Urine Pregnancy Test (in women with childbearing potential)[I]		X				X					X			
12-lead EKG	X^{H}													
Appropriate Radiographic Evaluation	X								X^{B}		X^{B}			
Other tumor biomarkers as appropriate (CA-125, PSA, CEA)	X^{D}								X^{D}		X^{D}	X^{D}		
Archival Tumor Block	X													
Plasma for cfDNA/PBMCs^	X^{E}	X^{E}	X^{E}	X^{E}	X^{E}				X^{E}		X^{E}	X^{E}	X^{E}	
Blood for germline DNA	X													
Tumor Biopsies	X^{F}			X^{G}								X^{A}		
Survival Status														X

Additional analyses of blood and/or tissue may be performed as new technology and/or information becomes available.
*CBC with differential will be obtained weekly given that talazoparib can cause grade 3/4 hematologic toxicity even after cycle 1. These labs can be obtained at the patient's local physician's office or lab and faxed to the study coordinator.
^Blood for PBMCs/cfDNA is drawn in the same tube. Two separate blood draws are not required.
[A] At the time of progression, an optional biopsy may be obtained from patients who have had stable disease for ≥3 months or who have had an objective response with subsequent progression.

Assessment Tool	Baseline (within 4 weeks of C1D1)	Cycle 1				Cycle 2 to Cycle 12				Cycle 13 and Beyond[J]		End of Treatment Visit	Safety Follow-Up Visit[C]	Survival Follow-Up
		Week				Week				Q4W	Q12W	At time of Discontinuation	30 (± 5) Days Post Discontinuation	Every 12 weeks for 1 year
		1	2	3	4	1	2	3	4					

[B] Per RECIST 1.1, assessments will be performed by the Investigator using CT or MRI scan every 8 weeks. After 6 months, radiographic assessment may occur every 12 weeks per investigator’s decision. Response should be confirmed by a repeat radiographic assessment not less than 4 weeks from the date the response was first documented.

[C.] The safety follow-up will take place 30 days after being taken off protocol to evaluate any lingering AEs.

[D] Tumor makers are optional and if done, should be repeated with restaging radiographic studies.

[E] Blood for PBMC/cfDNA will be drawn at the same time of routine blood draws for protocol as indicated. Ideally, the baseline blood draw will happen the day of baseline tumor biopsy, prior to said biopsy. Further blood will be drawn prior to the start of each new cycle with the labs drawn for clearance of the new cycle. Starting from Cycle 13, patients may have this blood collection every 12 weeks ± 4 days.

[F] Baseline tumor biopsy should be scheduled within 15 days of cycle 1, day 1.

[G] On-treatment biopsy will ideally be scheduled 3-6 hours after dose.

[H] These procedures should be performed within 72 hours of cycle 1, day 1.

[I] A positive urine pregnancy test will be confirmed by a serum pregnancy test.

[J] Starting from Cycle 13, patients with clinical benefit (CR, PR or SD >24 weeks) may return to the clinic every 12 weeks (Q12W) ± 4 days for study assessments and may have local laboratory assessments every 4 weeks (Q4W) ± 4 days.

6. TRIAL INFORMATION AND PROCEDURES

6.1 Trial Design

This is an open-label, single-center, phase II study exploring the efficacy and safety of talazoparib monotherapy in a diverse population of patients with cancers found to harbor somatic mutations or deletions of *BRCA1* or *BRCA2*, mutations or homozygous deletions in other *BRCA* pathway genes, and germline mutations in *BRCA1* or *BRCA2* with cancers other than breast or ovarian and for whom talazoparib is deemed the best treatment option in the opinion of the Investigator.

Patients with *BRCA1 / BRCA2* germline mutations will be identified as part of standard-of-care on a Clinical Laboratory Improvement Amendments (CLIA) laboratory platform. Patients with *BRCA1/BRCA2* somatic mutations, who do not have breast or ovarian cancer, will be identified by whole exome sequencing at a research laboratory, such as the MD Anderson Genomics Laboratory, an outside academic, or a commercial site. Homozygous deletions may be determined by next generation sequencing, copy number array or fluorescence in situ hybridization (FISH) in the CLIA environment.

The trial will consist of a screening period (Day –28 to −1), a treatment period, an end-of-treatment visit occurring when study medication is discontinued for any reason, a safety follow-up visit occurring 30 days (±5 days) after the last dose of study medication and a survival follow-up period lasting for a maximum of 12 months for each patient after their last dose of study medication to monitor survival status. Day 1 of the study (baseline) will be defined as the first day a patient receives study medication. One cycle of therapy will be defined as 28 days of treatment. Patients will be asked to attend clinic visits at regular intervals during the study for safety and efficacy assessments.

6.2 Treatment Plan

The study will include 5 cohorts of patients with the following:

1) Somatic mutations or deletions of *BRCA1* or *BRCA2*

2) Mutations or homozygous deletions in Other *BRCA* Pathway Genes
    a. *ATM*
    b. *PALB2*
    c. other genes, e.g. *Fanconi Anemia genes, ARID1A, MER11, RAD50, NBS1, ATR*; amplification of EMSY
3) Germline *BRCA1* or *BRCA2* mutations (not breast or ovarian cancer)

Enrolled patients will receive continuous oral dosing of talazoparib at 1mg po daily.

6.3 Trial Procedures

The Trial Flow Chart - Section 5 summarizes the trial procedures to be performed at each visit. Individual trial procedures are described in detail below. It may be necessary to perform these procedures at unscheduled time points if deemed clinically necessary by the investigator. Dosing, laboratory tests, imaging studies and office visits will occur per protocol (within ± 4 days) unless patients' medical or logistical issues necessitate adjustment.

Furthermore, additional evaluations/testing may be deemed necessary by the investigator for reasons related to subject safety.

6.3.1 Administrative Procedures

6.3.1.1 Informed Consent

The investigator or qualified designee must obtain documented consent from each potential subject or each subject's legally acceptable representative prior to participating in the clinical trial.

6.3.1.1.1 General Informed Consent

Consent must be documented by the subject's dated signature or by the subject's legally acceptable representative's dated signature on a consent form along with the dated signature of the person conducting the consent discussion.

A copy of the signed and dated consent form should be given to the subject before participation in the trial.

The initial informed consent form, any subsequent revised written informed consent form and any written information provided to the subject must receive the IRB/ERC's approval/favorable opinion in advance of use. The subject or his/her legally acceptable representative should be informed in a timely manner if new information becomes available that may be relevant to the subject's willingness to continue participation in the trial. The communication of this information will be provided and documented via a revised consent form or addendum to the original consent form that captures the subject's dated signature or by the subject's legally acceptable representative's dated signature.

Specifics about a trial and the trial population will be added to the consent form template at the protocol level.

The informed consent will adhere to IRB/ERC requirements, applicable laws and regulations and the University of Texas MD Anderson Cancer Center requirements.

6.3.1.2 **Inclusion/Exclusion Criteria**

All inclusion and exclusion criteria will be reviewed by the investigator or qualified designee to ensure that the subject qualifies for the trial.

6.3.1.3 **Medical History**

A medical history will be obtained by the investigator or qualified designee. Medical history will include all active conditions, and any condition diagnosed within the prior 10

years that are considered to be clinically significant by the Investigator. Details regarding the disease for which the subject has enrolled in this study will be recorded separately and not listed as medical history.

6.3.1.4 Prior and Concomitant Medications Review

6.3.1.4.1 Prior Medications

The investigator or qualified designee will review prior medication use, including any protocol-specified washout requirement, and record prior medication taken by the subject within 28 days before starting the trial. Treatment for the disease for which the subject has enrolled in the study will be recorded separately and not listed as a prior medication.

6.3.1.4.2 Concomitant Medications

The investigator or qualified designee will record medication, if any, taken by the subject during the trial. All medications related to reportable SAEs should be recorded.

6.3.1.5 Disease Details and Treatments

6.3.1.5.1 Disease Details

The investigator or qualified designee will obtain prior and current details regarding disease status.

6.3.1.5.2 Prior Treatment Details

The investigator or qualified designee will review all prior cancer treatments including systemic treatments, radiation and surgeries.

6.3.1.5.3 Subsequent Anti-Cancer Therapy Status

The investigator or qualified designee will review all new anti-cancer therapy initiated after the last dose of trial treatment. If a subject initiates a new anti-cancer therapy within 30 days after the last dose of trial treatment, the 30 day Safety Follow-up visit must occur before the first dose of the new therapy. Once new anti-cancer therapy has been initiated the subject will move into survival follow-up.

6.3.1.6 Trial Compliance (Medication/Diet/Activity/Other)

Interruptions from the protocol specified treatment plan for greater than 3 weeks between talazoparib doses due to toxicity require consultation between the sub-investigator and the principal investigator (PI) and written documentation of the collaborative decision on subject management.

6.3.2 Clinical Procedures/Assessments

6.3.2.1 Adverse Event (AE) Monitoring

The investigator or qualified designee will assess each subject to evaluate for potential new or worsening AEs as specified in the Trial Flow Chart and more frequently if clinically indicated. Adverse experiences will be graded and recorded throughout the study and during the follow-up period according to NCI CTCAE Version 4.0. Toxicities will be characterized in terms regarding seriousness, causality, toxicity grading, and action taken with regard to trial treatment.

Please refer to Section 6.4 for detailed information regarding the assessment and recording of AEs.

6.3.2.2 Physical Exam

6.3.2.2.1 Full Physical Exam

The investigator or qualified designee will perform a complete physical exam during the screening period. The initial (screening/baseline) complete physical examination should include the evaluation of the head, eyes, ears, nose, and throat (HEENT) and the cardiovascular, dermatological, musculoskeletal, respiratory, gastrointestinal, and neurological systems. Clinically significant abnormal findings should be recorded as medical history.

As part of the full physical exam, a medical history will also be collected, including demographics, relevant medical history, previous and current diseases, prior therapies including surgeries and relative responses, prior skin cancer history, therapies and procedures.

6.3.2.2.2 Directed Physical Exam

Subsequent physical examinations during the study for safety assessment may be restricted to evaluation of specific systems or areas of interest, including those with previously abnormal findings or associated with symptomatic or laboratory evidence of toxicity.

After the first dose of trial treatment new clinically significant abnormal findings should be recorded as AEs.

6.3.2.3 Vital Signs

The investigator or qualified designee will take vital signs at screening, prior to the full physical exam or directed physical exam and at treatment discontinuation.

Vital signs should include temperature, pulse, respiratory rate, weight and blood pressure. Height will be measured at screening only.

6.3.2.4 Eastern Cooperative Oncology Group (ECOG) Performance Scale

The investigator or qualified designee will assess ECOG status at screening, prior to the full physical exam or directed physical exam and discontinuation of trial treatment.

6.3.2.5 Tumor Imaging and Assessment of Disease

Tumor response will be assessed according to RECIST, v1.1, criteria.[38] Assessments will be performed by the Investigator using computed tomography (CT) or magnetic resonance imaging (MRI) scan every 8 weeks.

Patients may be allowed to continue the treatment after 2 cycles if there is continued clinical response or disease stabilization, and patients do not have significant toxicities. Patients will be treated until evidence of progressive disease. There is no maximum to the number of cycles a patient may receive if the patient is benefiting clinically.

6.3.2.5.1 Initial Tumor Imaging

Initial tumor imaging must be performed within 28 days prior to the first dose of trial treatment. The site study team must review pre-trial images to confirm the subject has measurable disease per RECIST 1.1.

Scans performed as part of routine clinical management are acceptable for use as the screening scan if they are of diagnostic quality and performed within 28 days prior to the first dose of trial treatment. The same imaging technique should be used in a subject throughout the study.

6.3.2.5.2 Tumor Imaging During Trial

Tumor imaging may be performed by CT or magnetic resonance imaging (MRI), but the same imaging technique should be used in a subject throughout the trial. Imaging should be performed every 8 weeks (56 days ± 7 days) from the first dose of trial treatment or more frequently if clinically indicated.

Per RECIST 1.1, response should be confirmed by a repeat radiographic assessment not less than 4 weeks from the date the response was first documented. The scan for confirmation of response may be performed at the earliest 4 weeks after the first indication of response, or at the next scheduled scan, whichever is clinically indicated.

After 6 months, radiographic assessment may occur every 12 weeks per investigator's decision. Imaging should continue to be performed until documented disease progression, the start of new anti-cancer treatment, withdrawal of consent, death, or the end of the study, whichever occurs first.

6.3.2.6 **Tumor Tissue Collection and Correlative Studies Blood Sampling**

Enrollment in this study is limited to those subjects with tumors that are characterized prior to enrollment as having:

1) Somatic mutations or deletions of *BRCA1* or *BRCA2*
2) Mutations or homozygous deletions in Other *BRCA* Pathway Genes
    a. *ATM*
    b. *PALB2*
    c. other genes, e.g. *Fanconi Anemia genes, ARID1A, MER11, RAD50, NBS1, ATR*; amplification of EMSY
3) Germline *BRCA1* or *BRCA2* mutations (not breast or ovarian cancer)

Archival Tumor Assessment:

Archival formalin fixed paraffin embedded (FFPE) tissue from primary tumors and/or if available biopsy of recurrence or metastases will be used for initial tumor genomic analysis. Patients who are scheduled to undergo a biopsy (for research) will undergo two additional core/punch biopsies, to allow for *PTEN* testing (as well as *PIK3CA*

testing), as well as research biopsies described below (1-2 punches or 3 cores and 4 FNAs).

Tumor Biopsies:

Patients will undergo pre-treatment tumor biopsies (within 15 days prior to Cycle 1 Day 1) and between Cycle 1 Day 15 through Day 18. On-treatment biopsy should ideally occur within 3-6 hours of dosing if possible. Biopsy type, site and number will be chosen based on sites of disease involvement and relative risk of biopsies. Patients will undergo 1-2 punch or 3 core biopsies and 4 FNAs. Core biopsies will be FFPE. One FNA will be immediately frozen in liquid nitrogen and then stored in -80 for transcriptional profiling. Three FNAs will be immediately frozen in liquid nitrogen and then stored in -80 for use in proteomic analysis (RPPA, and if adequate protein, multiplex proteomics) and DNA analysis as described for archival samples. Samples will be stored in the Department of Investigational Cancer Therapeutics (ICT) Laboratory directed by Dr. Meric-Bernstam.

Patients need to have biopsiable disease to enroll on cohort 1-2. If it is determined that the patient cannot safely be biopsied, then the patient may still be considered for enrollment after discussion with the principal investigator of the study. Patients eligible for Cohort 5 with a germline *BRCA* alteration can be enrolled even if they do not have biopsiable disease.

Pretreatment biopsies will be mandatory. On-treatment biopsy will be optional for the first 10 patients. If there is a signal for response and we begin to expand enrollment, biopsies will become mandatory for patients 11-30.

At the time of progression, an optional biopsy may be obtained from patients who have had stable disease for >3 months or who have had an objective response with subsequent progression.

Tumor Analysis:

Pretreatment biopsy and post-treatment biopsy (week 3) will be obtained for exploratory analysis to include:

1) Targeted exome sequencing for *BRCA* pathway mutations and other somatic and germline alterations
2) *BRCA* pathway copy number changes
3) *PTEN* and gamma-H2A.X by immunohistochemistry (IHC)
4) Transcriptional profile to assess *BRCA*-ness signature and putative PARP sensitivity predictors
5) Functional proteomics with reverse phase protein array

6.3.2.6.1 Somatic DNA Analysis:

Targeted exome sequencing will be performed to assess mutations in cancer-related genes. In selected patients (such as unusual responders) whole exome or whole genome sequencing may be performed. Copy number assessment may also be performed through the copy number array such as SNP array or MIP arrays (Affymetrics). DNA analysis may be performed at the MD Anderson Genomics Laboratory or in an outside academic or commercial site through a formal collaboration or contract service arrangement (e.g. Broad Institute, Foundation medicine). Collaborators will be blinded to patient identifiers for non-CLIA research assays.

6.3.2.6.2 Germline Testing:

BRCA 1/2 germline mutations will be performed as part of standard-of-care on a CLIA laboratory platform on all patients that have been tested for *BRCA1/2* somatic mutations in accordance with cohort 1 of Section 1.1.

6.3.2.6.3 Research Germline Testing

Germline DNA will be collected as a control targeted or whole exome sequencing. Given the rapid advances in genomic technology, it is possible that by the completion of this trial, full genomic sequencing will be affordable. In that case, we will use the state of the art technology for genomic sequencing.

Patients enrolled on this study will be approached for co-enrolment on PA12-1099; Germline testing for the MD Anderson Cancer Center Personalized Cancer Therapy Program as well as PA11-0852. These protocols will seek patient preferences on return of additional deleterious germline results.

6.3.2.6.4 IHC for Correlative Studies

We will determine the effect on DNA damage, cell proliferation by comparing gamma-H2A.X and Ki-67 by immunohistochemistry (IHC) at baseline and at 2 weeks. Core/punch biopsies will be formalin-fixed and paraffin embedded, and stained for Ki-67 in the clinical labs (MIB-1 antibody, Dako). Gamma-H2A.X and Ki-67 will be scored as % positive cells. We will determine whether there is a change % gamma-H2A.X+ and Ki-67+ cells before vs. 2 wks. Apoptosis will be assessed by cleaved caspase 3. Growth index will be calculated as Ki-67/apoptosis.

We will determine the effect of treatment on cell signaling by comparing pre-treatment and 2 week biopsies by IHC. Markers assessed will include PTEN, p-AktSer473, p-S6Ser235/236, Ser240/244, and p4E-BP1Thr70, ERKThr202/Tyr204, p-MET, INPP4B (by prioritization). Additional markers will be added as tissue availability permits. Positive and negative controls will be run with each assay. An H score will be determined by estimation of the percentage of tumor cells positively stained with low, medium, or high staining intensity. Markers will be assessed by a breast pathologist blinded to outcomes and treatment. We will compare the H-score at baseline to the H-score at 2 weeks, and we will compare the archival IHC staining to that on baseline biopsies.

6.3.2.6.5 Reverse Phase Proteomic Arrays (RPPA)

RPPA will be used to assess effect of talazoparib on cell signaling and DNA damage in the tumors, and in PBMCs. Briefly, lysis buffer will be used to lyse FNAs and PBMCS. Tumor lysates will be normalized to 1 µg/µL concentration as assessed by bicinchoninic acid assay (BCA) and then boiled with 1% SDS. The supernatants will be subsequently manually diluted in five-fold serial dilutions with lysis buffer. An Aushon Biosystems (Burlington, MA) 2470 arrayer created 1,056 sample arrays on nitrocellulose-coated FAST slides (Schleicher & Schuell BioScience, Inc.) from the serial dilutions. Slides will be probed with validated primary antibodies for RPPA (currently about over 150 antibodies) and the signal will be amplified using a DakoCytomation–catalyzed system. Secondary antibodies will be used as a starting point for amplification. The slides will be scanned, analyzed, and quantitated using Microvigene software (VigeneTech Inc., Carlisle, MA) to generate serial dilution–signal intensity curves for each sample. The RPPA spot signal intensity data will be quantified using MicroVigene (VigeneTech, Inc., Carlisle, MA) and processed by the R package SuperCurve (version 1.01), available at "http://bioinformatics.mdanderson.org/OOMPA". A fitted curve (called "supercurve") will be plotted with the signal intensities on the Y-axis and the relative log2 concentration of each protein on the X-axis using the non-parametric, monotone increasing B-spline model. The protein concentrations will be derived from the supercurve for each sample lysate on the slide by curve-fitting and then normalized by median polish. Each protein measurement will be subsequently corrected for loading.

6.3.2.6. Transcriptional Profiling

We will perform microarray profiling as an exploratory analysis to identify novel genes regulated at the RNA level in patient samples. RNA will be extracted from FNA samples using the RNAeasy Kit (Qiagen, Valencia, CA). The amount and quality of RNA will be considered adequate for further analysis if the optical density $_{260/280}$ ratio is ≥1.8 and the total RNA yield is ≥1µg. Adequate RNA for microarray profiling, without amplification, from a single FNA pass can be obtained in 90% of cases.

The technology for transcriptional profiling is rapidly evolving. RNA-seq may be utilized as an alternate platform if adequate amount of RNA can be obtained.

6.3.2.7 Circulating Biomarkers

Peripheral blood mononuclear cells (PBMCs) will be collected at baseline, pretreatment on Day 1 of cycle 1, weekly during cycle 1, and during week 4 (prior to cycle 2, day 1) for assessment of PARP levels and PAR-producing activity. Thereafter prior to each subsequent cycle, the patient will have a tube of blood drawn with the labs drawn for clearance of that cycle. Starting from Cycle 13, patients may have this blood collection every 12 weeks ± 4 days.

The protocol for PBMC and plasma processing is in Appendix E. PBMCs and plasma will be stored in -80 in the ICT lab. PBMCs and platelets will be analyzed with ELISA and/or western blotting for PARP levels and PAR activity, as well as RPPA, multiplex proteomics as exploratory endpoints.

Plasma for cell-free DNA (cfDNA) will be collected at baseline, on Day 1 of cycle 1, weekly during cycle 1, and during week 4 (prior to cycle 2, day 1. Thereafter prior to each subsequent cycle, the patient will have a tube of blood drawn with the labs drawn for clearance of that cycle. Starting from Cycle 13, patients may have this blood collection every 12 weeks ± 4 days.

Please note that blood for PBMC and cfDNA are drawn into the same tube and do not constitute two separate blood draws. Further when possible, these samples will be drawn during the routine labs drawn during treatment on this protocol.

Plasma at these time points will also be collected for exploratory proteomic analysis. Plasma will be kept at -80, and processed and free DNA will be extracted and kept at -80 prior to mutation analysis. Plasma and plasma DNA will be stored in the ICT lab.

Plasma may be sent to academic or commercial collaborators. Collaborators will be blinded to patient identifiers. Collaborating sites may include, but are not limited to: Foundation Medicine, Boreal, Illumina, Beaming, Sysmex, Genomic Health, Broad Institute, and Johns Hopkins.

6.3.2.8 Tumor Marker Assessment (Optional)

Tumor marker assessment is not an additional study-related laboratory evaluation. The purpose is to collect information that may be a part of standard clinical assessment for certain tumor types. Data collection (if applicable and if available) will occur at the Screening visit and at restaging every 8 weeks. Upon discontinuation, tumor marker data collection will occur every 8 weeks coinciding with post-treatment imaging follow-up visits.

Table 4 shows tumor types that are commonly evaluated using markers.

Table 4 Tumor Markers and Associated Tumor Types

Tumor Type	Tumor Marker
Carcinoid Tumors	Chromogranin A (CgA)
	5-Hydroxyindoleacetic acid (5-HIAA) (24 hours in urine)
Colon or Rectal Adenocarcinoma	Carcinoembryonic Antigen (CEA)
Neuroendocrine Carcinoma	Chromogranin A (CgA)
	5-Hydroxyindoleacetic acid (5-HIAA) (24 hours in urine)
Pancreas Adenocarcinoma	Carbohydrate Antigen 19-9 (CA 19-9)
Prostate Adenocarcinoma	Prostate-Specific Antigen (PSA)
Thyroid Cancer	Thyroglobulin (Tg)

6.3.3 Other Procedures

6.3.3.1 Withdrawal/Discontinuation

When a subject discontinues/withdraws from participation in the trial, all applicable activities scheduled for the final trial visit should be performed at the time of discontinuation. Any adverse events which are present at the time of

discontinuation/withdrawal should be followed in accordance with the safety requirements outlined in Section 6.4 - Assessing and Recording Adverse Events.

6.3.3.2 Visit Requirements

Visit requirements are outlined in Section 5 - Trial Flow Chart.

6.3.3.2.1 Screening Period

Approximately 28 days prior to enrollment, potential subjects will be evaluated to determine that they fulfill the entry requirements as set forth in Section 4.1. Visit requirements are outlined in Section 5 – Trial Flow Chart.

6.3.3.3 Post –Treatment Visits

6.3.3.3.1 Safety Follow-Up Visit

The mandatory Safety Follow-Up Visit should be conducted approximately 30 days after the last dose of trial treatment or before the initiation of a new anti-cancer treatment, whichever comes first. All AEs that occur prior to the Safety Follow-Up Visit should be recorded. Subjects with an AE of Grade >1 will be followed until the resolution of the AE to Grade 0-1 or until the beginning of a new anti-cancer therapy, whichever occurs first. SAEs that occur within 90 days of the end of treatment or before initiation of a new anti-cancer treatment should also be followed and recorded.

6.3.3.3.2 Survival Follow-up

Once a subject experiences confirmed disease progression or starts a new anti –cancer therapy, the subject moves into the survival follow-up phase and should be contacted by telephone every 12 weeks for a maximum of 12 months to assess for survival status until death, withdrawal of consent, or the end of the study, whichever occurs first.

6.4 Assessing, Recording, and Reporting Adverse Events

6.4.1 Adverse Events

According to the ICH definition, an adverse event (AE) is any untoward medical occurrence in a patient or clinical investigation subject administered a pharmaceutical product, and that does not necessarily have a causal relationship with this treatment. An AE can therefore be any unfavorable and unintended sign (including an abnormal laboratory finding), symptom, or disease temporally associated with the use of a medicinal (investigational) product, whether or not considered related to the investigational product.

This definition includes intercurrent illnesses or injuries that represent an exacerbation (increase in frequency, severity, or specificity) of pre-existing conditions. Whenever possible, it is preferable to record a diagnosis as the AE term rather than a series of terms relating to a diagnosis.

Adverse event information will be collected in an ongoing fashion through patient reporting AEs to their physician or health care provider. Seriousness and relatedness will be assessed by the treating physician, with appropriate reporting. A designated primary contact person based at the treatment center will be responsible for the collection and reporting of AEs for patients participating in the program.

The Investigator or physician designee is responsible for verifying and providing source documentation for all adverse events and assigning the attribution for each event for all subjects enrolled on the trial.

This study will utilize the recommended adverse event recording guidelines for phase II trials as per Appendix F.

6.4.2 Serious Adverse Events (SAE) Reporting

An adverse event or suspected adverse reaction is considered "serious" if, in the view of either the investigator or the sponsor, it results in any of the following outcomes:

- Death
- A life-threatening adverse drug experience – any adverse experience that places the patient, in the view of the initial reporter, at immediate risk of death from the adverse experience as it occurred. It does not include an adverse experience that, had it occurred in a more severe form, might have caused death.
- Inpatient hospitalization or prolongation of existing hospitalization
- A persistent or significant incapacity or substantial disruption of the ability to conduct normal life functions.
- A congenital anomaly/birth defect.

Important medical events that may not result in death, be life threatening, or require hospitalization may be considered a serious adverse drug experience when, based upon appropriate medical judgment, they may jeopardize the patient or subject and may require medical or surgical intervention to prevent one of the outcomes listed in this definition. Examples of such medical events include allergic bronchospasm requiring intensive treatment in an emergency room or at home, blood dyscrasias or convulsions that do not result in inpatient hospitalization, or the development of drug dependency or drug abuse (21 CFR 312.32).

- **Important medical events as defined above, may also be considered serious adverse events. Any important medical event can and should be reported as an SAE if deemed appropriate by the Principal Investigator or the IND Sponsor, IND Office.**
- All events occurring during the conduct of a protocol and meeting the definition of a SAE must be reported to the IRB in accordance with the timeframes and

procedures outlined in "The University of Texas M. D. Anderson Cancer Center Institutional Review Board Policy for Investigators on Reporting Unanticipated Adverse Events for Drugs and Devices". Unless stated otherwise in the protocol, all SAEs, expected or unexpected, must be reported to the IND Office, regardless of attribution (within 5 working days of knowledge of the event).

- **All life-threatening or fatal events,** that are unexpected, and related to the study drug, must have a written report submitted within **24 hours** (next working day) of knowledge of the event to the Safety Project Manager in the IND Office.
- **Unless otherwise noted, the electronic SAE application (eSAE) will be utilized for safety reporting to the IND Office and MDACC IRB.**
- **Serious adverse events will be captured from the time of the first protocol-specific intervention, until 30 days after the last study treatment/intervention, unless the participant withdraws consent. Serious adverse events must be followed until clinical recovery is complete and laboratory tests have returned to baseline, progression of the event has stabilized, or there has been acceptable resolution of the event.**
- **Additionally, any serious adverse events that occur after the 30 day time period that are related to the study treatment must be reported to the IND Office. This may include the development of a secondary malignancy.**

Reporting to FDA:

- Serious adverse events will be forwarded to FDA by the IND Sponsor (Safety Project Manager IND Office) according to 21 CFR 312.32.

It is the responsibility of the PI and the research team to ensure that serious adverse events are reported according to the Code of Federal Regulations, Good Clinical Practices, the protocol guidelines, the sponsor's guidelines, and Institutional Review Board policy.

6.4.3 Pregnancy

If a study patient or study patient's partner becomes or is found to be pregnant during the study patient's treatment with the investigational product, the investigator must submit this information to the IRB per institutional standard policy on a Serious Adverse Event (SAE) report form and Exposure in Utero (EIU) Supplemental Form, regardless of whether an SAE has occurred. In addition, the investigator must submit information regarding environmental exposure to the products in a pregnant woman (e.g., a patient reports that she is pregnant and has been exposed to a cytotoxic product by inhalation or spillage) using the EIU Form. This must be done irrespective of whether an adverse event has occurred and within 24 hours of awareness of the exposure. The information submitted should include the anticipated date of delivery (see below for information related to termination of pregnancy).

Follow-up is conducted to obtain general information on the pregnancy and its outcome for all EIU reports with an unknown outcome. The investigator will follow the pregnancy until completion (or until pregnancy termination) and notify the IRB of the outcomes a follow up to the initial EIU Supplemental Form. In the case of a live birth, the structural integrity of the neonate can be assessed at the time of birth. In the event of a termination, the reason(s) for the termination should be specified and, if clinically possible, the structural integrity of the terminated fetus should be assessed by gross visual inspection (unless pre-procedure test findings are conclusive for a congenital anomaly and the findings are reported).

If the outcome of the pregnancy meets the criteria for a serious adverse event (ie, ectopic pregnancy, spontaneous abortion, intrauterine fetal demise, neonatal death, or congenital anomaly [in a live born baby, a terminated fetus, an intrauterine fetal demise or a neonatal death]), the investigator should follow the procedures for reporting serious adverse events.

Additional information about pregnancy outcomes that are reported as serious adverse events follows:

- Spontaneous abortion includes miscarriage and missed abortion.
- Neonatal deaths that occur within 1 month of birth should be reported, without regard to causality, as serious adverse events.

• In addition, infant deaths after 1month should be reported as SAEs when the investigator assesses the infant death as related or possibly related to exposure to the investigational product.

Additional information regarding the exposure during pregnancy may be requested by the investigator. Further follow-up of birth outcomes will be handled on a case-by-case basis (e.g., follow-up on preterm infants to identify developmental delays).

6.4.4 SAE and Pregnancy Reporting to Pfizer

For subjects who have received talazoparib, SAEs must be reported to Pfizer U.S. clinical Trial Department immediately upon awareness of a death or life-threatening event, and within 24 hours of first awareness of all other reportable SAEs. The SAEs must be reported by completing the Pfizer-provided Investigator-Initiated Research Serious Adverse Event (IIR SAE) Report Form and submitting the Pfizer-provided Reportable Events Fax Cover Sheet to:

1-866-997-8322

The SAE report must contain at least a description of an SAE (diagnosis or signs and symptoms) or an otherwise reportable event (i.e. exposure during pregnancy, exposure during breastfeeding and occupational exposure) and a causality assessment. Causality assessment is the determination of whether there is a reasonable possibility that talazoparib caused or contributed to the SAE. For cases where talazoparib is determined NOT to be casually related to the SAE, provide information in the Narrative section of the reporting form on **other possible causes** of the SAE, including:

- Concomitant medications, including any protocol-specified background treatment
- Other illnesses
- Study procedures

Follow-up information (new, updated, or corrected information) should be forwarded to Pfizer U.S. clinical Trial Department using a new IIR SAE Report Form that includes the data that are new or revised from the previous report. Follow-up information should never be added to a previously submitted report form. Ensure that any new events included on a follow-up report are marked as serious and a causality assessment is provided for each of them.

6.5 Data Collection

All patients who meet eligibility criteria and are enrolled in this trial will be registered in Clinical Oncology Research Database at the University of Texas MD Anderson Cancer Center.

Data Protection and Confidentiality

All patients who meet eligibility criteria and are enrolled in this trial will be registered in Clinical Oncology Research e-Database (CORe) at the University of Texas MD Anderson Cancer Center at Houston. All protocol participants must be registered in the CORe. The date in the current informed consent document is displayed to ensure only the most current IRB approved version is used. Consent date, registration date, off study date, and evaluability data are required for all registrants.

The principal investigator agrees to keep all information and results concerning the study confidential. The confidentiality obligation applies to all personnel involved with this clinical trial. The Investigator must ensure that each participant’s anonymity will be maintained in accordance with applicable laws. The principal investigator should keep a separate log of ID numbers, names and addresses. Documents that contain the names associated with these ID numbers (e.g., written consent/assent forms) should be maintained by the Investigator in strict confidence except to the extent necessary to allow auditing by regulatory authorities, auditing or monitoring by the IRB.

The Principal Investigator shall obtain all such permissions and authorizations as may be necessary or desirable to allow the collection and use of information protected under federal privacy laws and state privacy laws, including permission/authorization for

monitoring and analysis (including re-analysis in combination with results of other studies), for regulatory submission purposes and for applicable reporting (if any).

Protocol specific data will be entered into PDMS/CORe/MOCLIA (or other database); PDMS/CORe/MOCLIA (or other database) will be used as the electronic case report form.

7. STATISTICAL CONSIDERATIONS

This section outlines the statistical analysis strategy and procedures for the study. If, after the study has begun, changes are made to primary and/or key secondary hypotheses, or the statistical methods related to those hypotheses, then the protocol will be amended. Changes to exploratory or other non-confirmatory analyses made after the protocol has been finalized, along with an explanation as to when and why they occurred, will be listed in the Clinical Study Report (CSR) for the study.

7.1 Statistical Analysis Plan Summary

This section contains a brief summary of the statistical analyses for this trial.

7.1.1 Efficacy Analyses

The primary and key secondary endpoints, primary analysis population, and statistical methods that will be employed for the efficacy analyses are presented in Table 5 below.

The primary hypothesis will be evaluated separately in each cohort by determining clinical benefit by RECIST 1.1. A sequential monitoring approach will be used following the time that a minimum of 10 subjects are enrolled in each cohort.

Statistical considerations are based on a parallel, multi-arm, phase II design with early stopping rules for futility. Early stopping rules will be applied separately to each of the 5

marker arms if >25% clinical benefit rate is unlikely. Each arm will be assessed for efficacy separately.

Table 5: Summary of Analysis Strategy for Key Efficacy Endpoints

Endpoint/Variable (Description, Timepoint)	Statistical Method	Analysis Population	Missing Data Approach
Primary Hypothesis #1:			
Clinical Benefit Rate by RECIST 1.1 in each cohort	Clinical benefit rates using posterior probabilities along with corresponding credible intervals.	Full Analysis set (FAS)	Missing observation counted as non- responder
Secondary Objectives – Within Indication			
PFS	Summary statistics using Kaplan-Meier method	FAS	Censored at last assessment
OS	Kaplan-Meier method	FAS	Censored at last assessment
Duration of Response (DOR)	Summary statistics using Kaplan-Meier method	All responders	Non-responders are excluded in analysis

We assume that the targeted level of activity is 25% clinical benefit or greater. Clinical benefit (CB) is defined as any of the following, complete response, partial response, or stable disease for $\geq$ 24 weeks.

We used the program MultcLeanDesktop Version 2.1.0 to generate an appropriate Bayesian design targeting a clinical benefit rate of 25%. We suppose that: θ_E = probability of clinical benefit on the experimental arm ~ beta (a_E, b_E) and θ_S = probability of clinical benefit for standard treatment in the historical data ~ beta (a_S, b_S) where a_S = 250, b_S = 750, a_E = 0.5, and b_E = 1.5. This specifies an informative prior for the standard with mean 0.25 and a non-informative prior for the experimental arm also with mean 0.25. We stop the trial early if Prob($\theta_S > \theta_E$ | data) > 0.95. The maximum sample size is set to 30 and we choose to monitor in cohorts of size 5. The stopping boundaries are 0/10, 1/15, 2/20, and 3/25 (where m/n indicates that we stop early if

after n patients we see m or fewer successes). If the true success probability is 5% then the probability of stopping early with these stopping boundaries is 0.97; for 10% it is 0.81; for 15%: 0.55; for 20%: 0.32; for 25%: 0.16; for 30%: 0.07; and for 35%: 0.03.

We will set the maximum sample size to 30 for each marker arm, except for cohort 2 which will enroll up to 30 patients for each subcohort. By grouping specific BRCA pathway gene mutations or deletions as subcohorts in cohort 2, we are able to analyze patient data with greater statistical confidence. If the observed CB rate is 25% with a maximum sample size of N=30, the true CB rate from the drug is between 14% and 38%, 90% of the time. Biomarker-drug combinations that meet these criteria will be recommended for further comparative evaluation in a larger trial. If the lower bound of the 90% posterior credible interval is ≥ 14%, the drug will be recommended for subsequent independent confirmatory study.

Five marker cohorts will be assessed simultaneously in this study, the minimum overall sample size to be treated is 50 (5 x 10) patients, if each arm is terminated early for futility. The maximum sample size for this study is 150 (30 X 5) patients. As of September 27, 2019, cohort 2 has been closed to enrollment as no significant responses were seen in this cohort. A total of 31 patients were enrolled in cohort 2: 10 patients in cohort 2a (mutations or deletions in ATM), 4 patients in cohort 2b (mutations or deletions in PALB2), and 17 patients in cohort 2c (mutations or deletions in other genes). A PTEN mutation/deletion arm, to which 14 patients were enrolled, was previously closed to enrollment due to lack of response to treatment. Because of this, the maximum sample size for this study has been reduced to 105 patients.

7.1.2 **Safety Analyses**

The All-Patients-as-Treated population will be employed for safety analyses. Safety will be monitored across all cohorts. A safety monitoring committee will meet monthly to evaluate the aggregate toxicity probability.

The Investigator is responsible for completing efficacy/safety summary reports, and submitting them to the IND Office Medical Affairs and Safety Group, for review and approval. These should be submitted after the first 10 evaluable patients per arm, complete 24 weeks of study treatment, and every 5 evaluable patients per arm, thereafter.

A copy of the cohort summary should be placed in the Investigator's Regulatory Binder under "sponsor correspondence".

7.1.3 **Statistical Considerations for Correlative Studies**

To determine baseline molecular markers (DNA, RNA and protein) that may predict clinical benefit.

Marker values will be compared between patients with and without clinical benefit using chi-squared or Fisher exact tests for categorically-scaled markers and Wilcoxon rank sum tests for interval- and ordinal-scaled markers. Due to the large number of candidate markers, only those significant on univariate analysis will be combined into a single logistic regression model to assess their independent effects on clinical benefit. Analyses will be performed both within and across the five cohorts. Results will be considered exploratory and thus no corrections will be made for multiple testing.

PARP signature and DNA repair activity will be performed as a planned analysis. Baseline expression of functional proteomics profile will be performed as exploratory analysis (false discovery rate, FDR of 0.3 as being significant).

To determine pharmacodynamic markers in blood and plasma that may predict outcome.

Regulation of PAR activity in PBMCs will be correlated with response. Effect of treatment on proteomic profile and cfDNA will be assessed as exploratory endpoints.

To determine concordance of BRCA1/2 alterations in archival tissue and pre-treatment biopsies and to determine concordance of genomic alterations in tumor and circulating free DNA.

The alterations are measured as either present or absent and thus their concordance between archival tissue and pre-treatment biopsies and between tumor and circulating free DNA will be assessed using kappa statistics.

Assessing effects of treatment on various markers

The markers described yield interval-scaled values that may not be normally distributed, so differences between baseline and 2-week values will be assessed using the Wilcoxon signed-rank test.

Baseline proteomic signature and pharmacodynamic response to talazoparib as assessed by RPPA may correlate with talazoparib growth inhibitory effects.

Pharmacodynamic effect of talazoparib on proteomic signature will be assessed by RPPA and correlated with response, clinical benefit, as well as best response as % tumor change.

Comparison of archival IHC staining to that on baseline biopsies

IHC staining will be assessed via the H-score which can be treated as an interval-scaled variable. We will use the concordance correlation coefficient as well as the Altman-Bland approach to assess the concordance between archival IHC staining and baseline biopsy IHC staining. In addition, PTEN will be assessed (PTEN loss, no loss).

Concordance between archival tissue and pre-treatment biopsies will be assessed using kappa statistics.

REFERENCES

1. Brochure Is: BMN 673 Version 5.0, in Inc. BP (ed), 2014

2. Birkelbach M, Ferraiolo N, Gheorghiu L, et al: Detection of impaired homologous recombination repair in NSCLC cells and tissues. J Thorac Oncol 8:279-86, 2013

3. Hennessy BT, Timms KM, Carey MS, et al: Somatic mutations in BRCA1 and BRCA2 could expand the number of patients that benefit from poly (ADP ribose) polymerase inhibitors in ovarian cancer. J Clin Oncol 28:3570-6, 2010

4. George J, Alsop K, Etemadmoghadam D, et al: Nonequivalent Gene Expression and Copy Number Alterations in High-Grade Serous Ovarian Cancers with BRCA1 and BRCA2 Mutations. Clin Cancer Res, 2013

5. Gorringe KL, George J, Anglesio MS, et al: Copy number analysis identifies novel interactions between genomic loci in ovarian cancer. PLoS One 5, 2010

6. Konishi H, Mohseni M, Tamaki A, et al: Mutation of a single allele of the cancer susceptibility gene BRCA1 leads to genomic instability in human breast epithelial cells. Proc Natl Acad Sci U S A 108:17773-8, 2011

7. Fong PC, Boss DS, Yap TA, et al: Inhibition of poly(ADP-ribose) polymerase in tumors from BRCA mutation carriers. N Engl J Med 361:123-34, 2009

8. Kummar S, Ji J, Morgan R, et al: A phase I study of veliparib in combination with metronomic cyclophosphamide in adults with refractory solid tumors and lymphomas. Clin Cancer Res 18:1726-34, 2012

9. Stefansson OA, Jonasson JG, Johannsson OT, et al: Genomic profiling of breast tumours in relation to BRCA abnormalities and phenotypes. Breast Cancer Res 11:R47, 2009

10. Shen Y, Rehman FL, Feng Y, et al: BMN 673, a novel and highly potent PARP1/2 inhibitor for the treatment of human cancers with DNA repair deficiency. Clin Cancer Res, 2013

11. Johann Sebastian De Bono LAM, Michael Gonzalez, Nicola J. Curtin, Evelyn Wang, Joshua W. Henshaw, Manpreet Chadha, Jasgit C. Sachdev, Daniela Matei, Gayle S. Jameson, Michael Ong, Bristi Basu, Zev A. Wainberg, Lauren Averett Byers, Rashmi Chugh, Andrew Dorr, Stanley B. Kaye, Ramesh K. Ramanathan;: First-in-human trial of novel oral PARP inhibitor BMN 673 in patients with solid tumors. J Clin Oncol 31, 2013 (suppl; abstr 2580) 2013

12. Chen Y, Zhang L, Hao Q: Olaparib: a promising PARP inhibitor in ovarian cancer therapy. Arch Gynecol Obstet, 2013

13. Gelmon KA, Tischkowitz M, Mackay H, et al: Olaparib in patients with recurrent high-grade serous or poorly differentiated ovarian carcinoma or triple-negative breast cancer: a phase 2, multicentre, open-label, non-randomised study. Lancet Oncol 12:852-61, 2011

14. Gilardini Montani MS, Prodosmo A, Stagni V, et al: ATM-depletion in breast cancer cells confers sensitivity to PARP inhibition. J Exp Clin Cancer Res 32:95, 2013

15. Lukaszewicz A, Howard-Till RA, Novatchkova M, et al: MRE11 and COM1/SAE2 are required for double-strand break repair and efficient chromosome pairing during meiosis of the protist Tetrahymena. Chromosoma 119:505-18, 2010

16. Oplustilova L, Wolanin K, Mistrik M, et al: Evaluation of candidate biomarkers to predict cancer cell sensitivity or resistance to PARP-1 inhibitor treatment. Cell Cycle 11:3837-50, 2012

17. Dedes KJ, Wilkerson PM, Wetterskog D, et al: Synthetic lethality of PARP inhibition in cancers lacking BRCA1 and BRCA2 mutations. Cell Cycle 10:1192-9, 2011

18. Chen P, Liang J, Wang Z, et al: Association of common PALB2 polymorphisms with breast cancer risk: a case-control study. Clin Cancer Res 14:5931-7, 2008

19. Buisson R, Dion-Cote AM, Coulombe Y, et al: Cooperation of breast cancer proteins PALB2 and piccolo BRCA2 in stimulating homologous recombination. Nat Struct Mol Biol 17:1247-54, 2010

20. Sourisseau T, Maniotis D, McCarthy A, et al: Aurora-A expressing tumour cells are deficient for homology-directed DNA double strand-break repair and sensitive to PARP inhibition. EMBO Mol Med 2:130-42, 2010

21. Hughes-Davies L, Huntsman D, Ruas M, et al: EMSY links the BRCA2 pathway to sporadic breast and ovarian cancer. Cell 115:523-35, 2003

22. McCabe N, Turner NC, Lord CJ, et al: Deficiency in the repair of DNA damage by homologous recombination and sensitivity to poly(ADP-ribose) polymerase inhibition. Cancer Res 66:8109-15, 2006

23. Shen J, Peng Y, Wei L, et al: ARID1A Deficiency Impairs the DNA Damage Checkpoint and Sensitizes Cells to PARP Inhibitors. Cancer Discov, 2015

24. Wu JN, Roberts CW: ARID1A mutations in cancer: another epigenetic tumor suppressor? Cancer Discov 3:35-43, 2013

25. Wilson BG, Roberts CW: SWI/SNF nucleosome remodellers and cancer. Nat Rev Cancer 11:481-92, 2011

26. Wu RC, Wang TL, Shih Ie M: The emerging roles of ARID1A in tumor suppression. Cancer Biol Ther 15:655-64, 2014

27. Wang X, Nagl NG, Jr., Flowers S, et al: Expression of p270 (ARID1A), a component of human SWI/SNF complexes, in human tumors. Int J Cancer 112:636, 2004

28. Wang X, Nagl NG, Wilsker D, et al: Two related ARID family proteins are alternative subunits of human SWI/SNF complexes. Biochem J 383:319-25, 2004

29. Imbalzano AN, Kwon H, Green MR, et al: Facilitated binding of TATA-binding protein to nucleosomal DNA. Nature 370:481-5, 1994

30. Imbalzano AN, Zaret KS, Kingston RE: Transcription factor (TF) IIB and TFIIA can independently increase the affinity of the TATA-binding protein for DNA. J Biol Chem 269:8280-6, 1994

31. Wang GG, Allis CD, Chi P: Chromatin remodeling and cancer, Part II: ATP-dependent chromatin remodeling. Trends Mol Med 13:373-80, 2007

32. Mathew CG: Fanconi anaemia genes and susceptibility to cancer. Oncogene 25:5875-84, 2006

33. Howlett NG, Taniguchi T, Olson S, et al: Biallelic inactivation of BRCA2 in Fanconi anemia. Science 297:606-9, 2002

34. Chu EC, Tarnawski AS: PTEN regulatory functions in tumor suppression and cell biology. Med Sci Monit 10:RA235-41, 2004

35. Mendes-Pereira AM, Martin SA, Brough R, et al: Synthetic lethal targeting of PTEN mutant cells with PARP inhibitors. EMBO Mol Med 1:315-22, 2009

36. McEllin B, Camacho CV, Mukherjee B, et al: PTEN loss compromises homologous recombination repair in astrocytes: implications for glioblastoma therapy with temozolomide or poly(ADP-ribose) polymerase inhibitors. Cancer Res 70:5457-64, 2010

37. Shen WH, Balajee AS, Wang J, et al: Essential role for nuclear PTEN in maintaining chromosomal integrity. Cell 128:157-70, 2007

38. Minami D, Takigawa N, Takeda H, et al: Synergistic effect of olaparib with combination of cisplatin on PTEN-deficient lung cancer cells. Mol Cancer Res 11:140-8, 2013

39. Gupta A, Yang Q, Pandita RK, et al: Cell cycle checkpoint defects contribute to genomic instability in PTEN deficient cells independent of DNA DSB repair. Cell Cycle 8:2198-210, 2009

40. Daemen A, Wolf DM, Korkola JE, et al: Cross-platform pathway-based analysis identifies markers of response to the PARP inhibitor olaparib. Breast Cancer Res Treat 135:505-17, 2012

41. Jung-min Lee CMA, John L. Hays, Anne M. Noonan, Lori M. Minasian, JoAnne Zujewski, Minshu Yu, Jiuping Jay Ji, Tristan Sissung, Nicole D. Houston, Elise C. Kohn: Phase I/Ib study of the PARP inhibitor olaparib (O) with carboplatin (C) in BRCA1/2 mutation carriers with breast or ovarian cancer (Br/OvCa) (NCT00647062).
. 2013

42. Cardnell RJ, Feng Y, Diao L, et al: Proteomic markers of DNA repair and PI3K pathway activation predict response to the PARP inhibitor BMN 673 in small cell lung cancer. Clin Cancer Res 19:6322-8, 2013
